# Structural basis of bis-quinolinium ligands binding to quadruplex–duplex hybrids from *PIM1* oncogene

**DOI:** 10.1093/nar/gkaf894

**Published:** 2025-09-12

**Authors:** Anirban Ghosh, Jakub Harnos, Petr Stadlbauer, Jiri Sponer, Martina Lenarcic Zivkovic, Lukas Trantirek

**Affiliations:** Central European Institute of Technology (CEITEC), Masaryk University, Brno 62500, Czech Republic; Department of Experimental Biology, Faculty of Science, Masaryk University, Brno 62500, Czech Republic; Institute of Biophysics, Czech Academy of Sciences, Brno 61200, Czech Republic; Department of Physical Chemistry, Faculty of Science, Palacký University Olomouc, Olomouc 77900, Czech Republic; Institute of Biophysics, Czech Academy of Sciences, Brno 61200, Czech Republic; Regional Center of Advanced Technologies and Materials, The Czech Advanced Technology and Research Institute (CATRIN), Palacký University Olomouc, Olomouc 78371, Czech Republic; Slovenian NMR Centre, National Institute of Chemistry, Ljubljana 1000, Slovenia; Central European Institute of Technology (CEITEC), Masaryk University, Brno 62500, Czech Republic

## Abstract

Our study investigates the interaction of two bis-quinolinium ligands, Phen-DC3 and 360A, with the quadruplex–duplex hybrid (QDH) derived from the promoter region of the *PIM1* oncogene. While the QDH is polymorphic *in vitro*, with a hybrid and antiparallel conformation, we demonstrate that it predominantly adopts the antiparallel conformation within the intracellular environment of *Xenopus laevis* oocytes (eukaryotic model system). Notably, both ligands selectively bind to the hybrid QDH conformation *in vitro* and in a cellular context. High-resolution nuclear magnetic resonance (NMR) structures of the complexes between the hybrid QDH and the ligands reveal distinct binding modes at the quadruplex–duplex (Q-D) junction. Specifically, Phen-DC3 binds rigidly, while 360A dynamically reorients between two positions. Our findings provide a crucial paradigm highlighting the differences in structural equilibria involving QDH *in vitro* compared to its behavior in the intracellular space. They also underscore the potential to modulate these equilibria under native-like conditions through ligand interactions. The observed differences in the binding of Phen-DC3 and 360A lay the groundwork for designing next-generation bis-quinolinium compounds with enhanced selectivity for the Q-D junction. Methodologically, our study illustrates the potential of ^19^F-detected in-cell NMR methodology for screening interactions between DNA targets and drug-like molecules under physiological conditions.

## Graphical Abstract

**Graphical Abstract:**
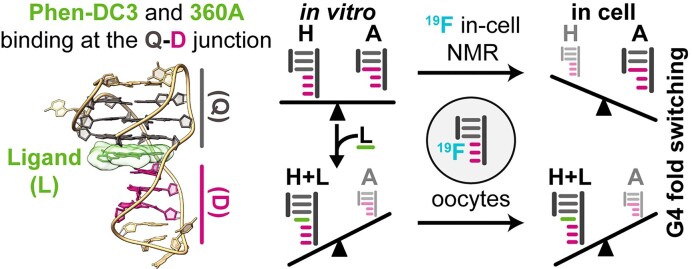

## Introduction

Transient melting of duplex DNA during transcription and replication enables intragenic G-rich sequences to fold into intramolecular G-quadruplexes (G4) [1]. The G4-forming sites are abundant in the human genome and overrepresented in evolutionarily conserved and functionally important regions, including the telomeres, centromeres, and promoter regions of (onco)genes [2, 3]. G4 structures can impede the progression of DNA and RNA polymerases and act as recognition sites for regulatory proteins [1, 4]. This dual functionality makes G4s critical regulators of essential biological processes such as DNA replication, DNA damage response, and gene expression [4, 5]. Due to their biological significance, targeting G4 structures with low-molecular-weight ligands to modulate their formation has emerged as a potential therapeutic strategy for various pathological conditions, including cancer and neurodegenerative diseases [6–8].

Although G4s share a common core motif of two or more stacked G•G•G•G tetrads, they adopt diverse folding topologies based on relative strand orientation and loop arrangement [9, 10]. These topological differences have guided efforts over the past two decades to develop G4 ligands that selectively target specific intragenic G4 structures [11, 12]. Despite some ligands showing preferential binding to particular G4 topologies, none have achieved the specificity to target individual G4 sites [11]. This limitation remains a significant barrier to translating G4-targeting strategies into effective therapies.

One promising approach is to improve the selectivity to target a unique subclass of G4 structures known as G-quadruplex–duplex hybrids (QDH) [13]. QDHs form when a G4 directly connects to a duplex stem-loop stabilized by Watson-Crick base pairs [14, 15]. This connection creates a G4-duplex (Q-D) junction, where the duplex is stacked on the terminal quartet through lateral or diagonal loops (coaxial arrangements) or caps the G4 groove *via* a propeller loop (orthogonal arrangements) [14]. Bioinformatics analyses have identified over 80 000 putative QDH-forming sequences across the human genome, inferring their essential roles in various (patho)biological and regulatory processes [13].

Efforts to specifically target QDHs often involve dual-function ligands that combine a G4-specific ligand with a duplex-binding moiety, typically a groove binder [16–23]. While the duplex-binding component has minimal design constraints beyond druggability, the G4-specific subunit must meet stringent requirements, including strong affinity and selectivity for the QDH interface and specificity toward the G4 topology within the QDH context. Among promising scaffolds for QDH-targeting ligands, Phen-DC3—a bis-quinolinium derivative—has shown high selectivity for G4 over double- and single-stranded DNA and preferentially binds Q-D junctions rather than exposed G-tetrads [24–26].

Interestingly, topological preferences of Phen-DC3 diverge on diverse G4 conformations containing non-duplex-forming (unstructured) loops. For example, Phen-DC3 forms a complex with the G4 of the c-Myc oncogene in a parallel topology [27]. On the contrary, it induces a structural transition from a hybrid-1 to an antiparallel chair-type G4 structure of a sequence derived from human telomeric repeats [28, 29]. These findings underscore the limitations of using simplified G4 models to predict ligand behavior in the QDH context and emphasize the need for a deeper understanding of ligand selectivity at the QDH interface.

**Figure 1.**
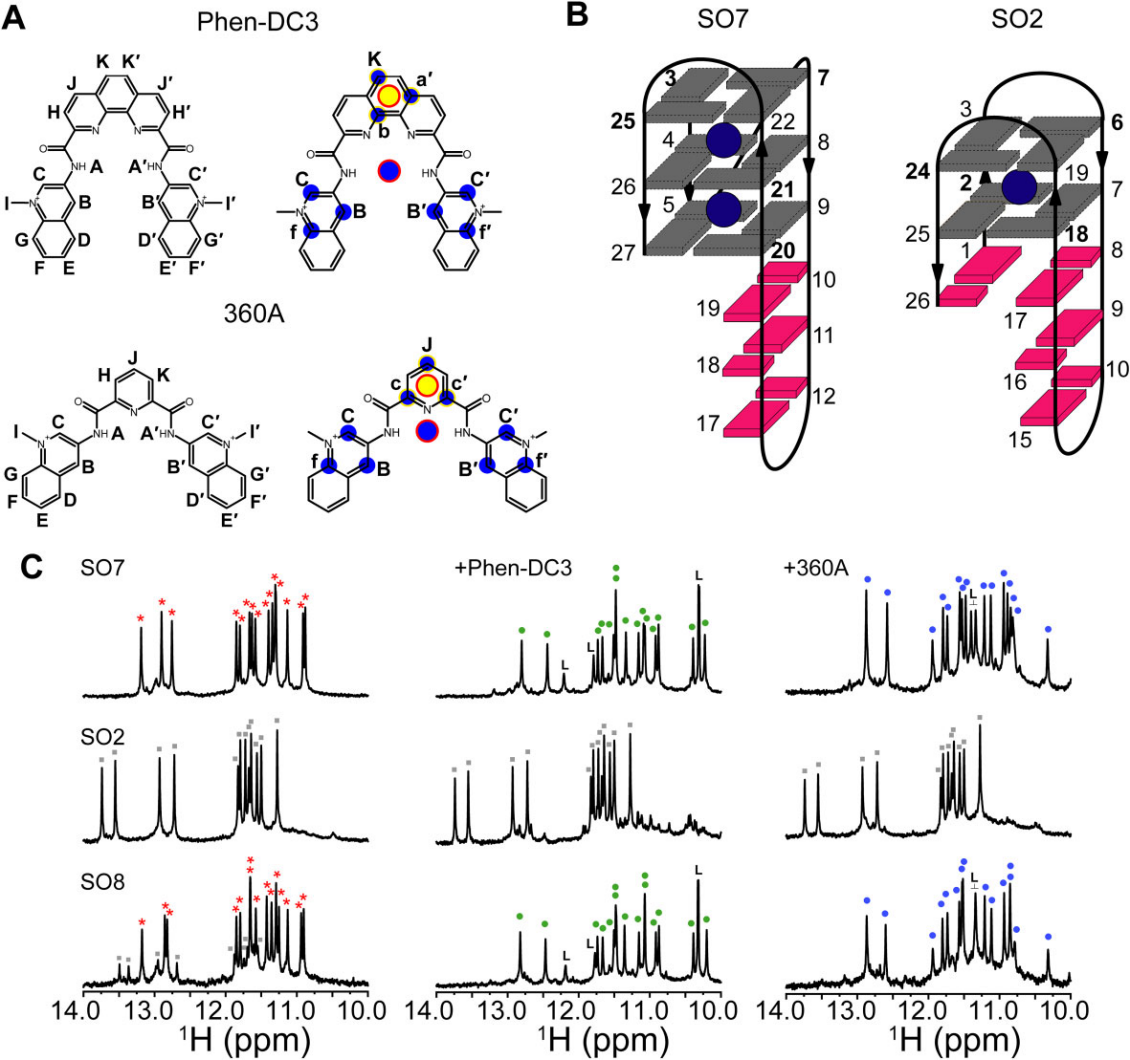
(**A**) Chemical structures of bis-quinolinium-based G4 ligands, Phen-DC3 and 360A, with the atom numbering on the left and with highlighted atoms used for the definition of collective coordinate (i.e. angular phase) in the well-tempered metadynamics simulation on the right; two points, marked by the red edge, used for the definition of the angular phase are related to the ligand atoms as follows: the blue circle represents the geometric center of all the highlighted atoms, while the yellow circle is the geometric center of three nearby atoms of the six-membered ring. (**B**) Hybrid-1 and antiparallel QDH topologies formed by sequences SO7 and SO2, derived from the *PIM1* gene promoter, respectively. Oligonucleotide SO8 is polymorphic and simultaneously forms hybrid (SO7-like) and antiparallel (SO2-like) QDHs in a ∼75%:20% ratio [36]. Sequences of SO7, SO2, and SO8 are shown in Table 1. G4 and duplex stem-loop units are colored gray and magenta, respectively. Guanines in *syn* glycosidic conformation are marked in bold. (**C**) Imino regions of the ^1^H NMR spectra of free SO7, SO2, and SO8 (left), and in the presence of 1:1 molar equivalents of Phen-DC3 (middle) and 360A (right), respectively. Signals belonging to free hybrid and antiparallel QDHs are marked with red asterisks and gray rectangles, while DNA–Phen-DC3 and DNA–360A complexes are denoted by green and blue dots, respectively. Signals belonging to ligand atoms are marked with L.

In this study, we examined the binding of two bis-quinolinium-based ligands, Phen-DC3 and 360A (Fig. 1A), to QDHs using solution nuclear magnetic resonance (NMR) and explicit solvent molecular dynamics (MD) simulation. Both ligands are widely used as benchmarks for evaluating new G4 ligands or developing novel ligand-screening methods [24, 28, 30–33]. While 360A stabilizes antiparallel G4 structures and intercalates into AGCGA-quadruplexes [28, 34, 35], its ability to bind G4 or QDH-forming sequences in a topology-specific manner was not explored. Although Phen-DC3 and 360A share a common scaffold of two quinolinium rings, they differ in their central moiety: Phen-DC3 features a phenanthroline unit, while 360A incorporates a smaller pyridine group. The structural differences allowed us to examine their distinct binding epitopes and topological preferences toward a model QDH system. For this purpose, we used sequences derived from the promoter region of the *PIM1* (Proviral Integration Moloney virus) oncogene, which is overexpressed in triple-negative breast cancer and forms two distinct QDH topologies in solution [36]. One of them is a three-layered hybrid G4 with three G-C Watson-Crick base pairs in the stem-loop, while the other forms an antiparallel-stranded QDH structure with two G-quartets, a G-C-G-C-quartet, and two G-C Watson-Crick base pairs in the middle stem-loop (Fig. 1B) [36].

Both ligands selectively bound to the hybrid folding topology of polymorphic *PIM1* QDH. High-resolution NMR structures revealed that Phen-DC3 and 360A reside in the Q-D junction, each adopting a unique arrangement in the binding pocket. Interestingly, polymorphic *PIM1* QDH under *in vitro* conditions predominantly adopted an antiparallel conformation in the crowded intracellular environment of *Xenopus laevis* oocytes (eukaryotic model system) [37]. However, upon adding Phen-DC3 and 360A, the selective binding to the hybrid QDH conformation was observed for both ligands with in-cell ^19^F NMR.

Our findings provide atomistic insights into the QDH–ligand interactions, offering valuable guidance for designing more selective QDH-targeting ligands. These results also expand the potential applications of QDH-based stimuli-responsive elements in nanotechnology [38–40].

## Materials and methods

The DNA sequences used in this study [isotopically unlabeled, residue-specific ^13^C/^15^N isotopically labeled, and modified with 2′-deoxy-2′-fluoro-arabino nucleic acid (FANA)] are listed in Table 1. Isotopically unlabeled DNA oligonucleotides were purchased from Generi Biotech (Czech Republic) and purified by n-butanol precipitation. Residue-specific ^13^C/^15^N isotopically labeled (∼4%–6% enrichment) and FANA-modified oligonucleotides were synthesized in-house using phosphoramidite chemistry on an H-8 DNA/RNA Synthesizer (K&A Labs GmbH) in DMT-on mode [41]. DNA phosphoramidites and synthesis reagents were purchased from Glen Research (Sterling, USA) and the Cambridge Isotope Laboratory (CIL, UK). Unless otherwise specified, all other chemicals were purchased from Merck Life Science (Czech Republic) and Sigma–Aldrich (USA). Oligonucleotide samples were dissolved in nuclease-free grade water (Ambion, Life Technologies S.A.S., Saint-Aubin, France) and stored at −20°C in aliquots.

**Table 1.** The list of isotopically unlabeled and FANA (^F^G) modified DNA sequences used in this study

	Name of the construct	Sequence (5′–3′)
QDH sequences derived from the *PIM1* gene promoter	SO8	GC**GGG**A**GGG**CGCGCCAGCG**GGG**TC**GGG**C
	SO7	GC**GGG**A**GGG**CGCGCCAGCG**GGG**TC**GGG**
	SO2	G**GG**AG**GG**CGCGCCAGCG**GG**GTCG**GG**C
Mutations in the duplex stem-loop of SO7	SO7_2d_	GC**GGG**A**GGG**GCGCCAGC**G** **GG**TC**GGG**
	SO7_1d_	GC**GGG**A**GGG**CGCCAG**GGG**TC**GGG**
SO7 without duplex stem-loop	SO7_nd_	GC**GGG**A**GGG**GCCA**GGG**TC**G** **GG**
SO7 with a disrupted base pair at the Q-D junction	SO7_TT_	GC**GGG**A**GGG**TGCGCCAGCT**GGG**TC**GGG**
Mutations in the Q-D junction of SO7	SO7_G10–C19_	GC**GGG**A**GGG**GGCGCCAGCC**GGG**TC**GGG**
	SO7_A10–T19_	GC**GGG**A**GGG**AGCGCCAGCT**GGG**TC**GGG**
	SO7_T10–A19_	GC**GGG**A**GGG**TGCGCCAGCA**GGG**TC**GGG**
FANA (^F^G) modified QDH sequences	SO8-F	GC**GGG**A**GGG**CGCGCCAGCG**GGG**TC**GG^F^G**C
	SO2-F	G**GG**AG**GG**CGCGCCAGCG**GG**GTCG**G^F^G**C

Guanines involved in G-quartet formation are in boldface.

Phen-DC3 (CAS Registry Number: 929895-45-4) and 360A (CAS Registry Number: 737763-37-0) ligands were purchased in powder form from Merck Life Science (Czech Republic) and GlpBio Technology, Inc. (USA), respectively. Stock solutions were prepared in DMSO-d6 (d-99.9%) (CIL, UK) at a concentration of 20 mM. The concentrations of stock solutions were confirmed spectrophotometrically using a BioSpectrometer (Eppendorf, Czech Republic) and extinction coefficient (ϵ) values from previous studies [24, 30]. Stock solutions were aliquoted and stored at −20°C.

The DNA samples were folded by annealing at 95°C for 2–3 min, followed by adding the appropriate amount of concentrated buffer (10×) to make a final 20 mM potassium chloride (KCl) and 20 mM potassium phosphate (KPi), pH 7.1 (NMR, CD buffer). Samples were gradually cooled from 95°C to 25°C over 72 h. NMR and circular dichroism (CD) samples with different DNA:ligand ratios were prepared by titrating 20 mM Phen-DC3 or 360A stock solution in DMSO-d6 into the aqueous solution of the QDH oligonucleotides. Samples were equilibrated at room temperature (∼25°C) for 24 h before NMR and CD measurements.

### Circular dichroism spectroscopy

The CD experiments were conducted on a J-815 spectrometer (Jasco, Japan) equipped with a Peltier temperature controller (Model no. PFD-425S) using a rectangular quartz cuvette with a 0.1 cm path length. CD spectra were recorded at 25°C in the 220–350 nm wavelength range, with a scanning speed of 50 nm/min, 2 nm bandwidth, 0.2 nm step resolution, and digital integration time of 1 s. Each CD spectrum represents the average of four scans and was baseline-corrected using the corresponding buffer. The DNA concentration in samples used for CD measurements was 20 μM, while the ligand (Phen-DC3, 360A) concentrations ranged from 0 to 60 μM. CD melting experiments were performed over a temperature range of 5–95°C, at a ramping rate of 1°C/ min, for the free DNA and DNA–ligand complexes at a 1:1, 1:2, and 1:3 molar ratios. Molar ellipticity at ∼290 nm was used to calculate the folded fractions (f) as a function of temperature. The melting temperature (*T*_m_) was calculated at a folded fraction of 0.5, assuming a two-state model with pre- and post-transitional baseline slopes (Table 2 and Supplementary Tables S1 and S2) [42].

**Table 2.** Changes in thermal stabilities of *PIM1*-derived sequences in the presence of Phen-DC3 and 360A at a 1:1 DNA:ligand molar ratio were evaluated from CD melting experiments

	*T*_m_ (°C)	Δ*T*_m_ (°C)^a^
SO7	61.6 ± 1.3	
SO7–Phen-DC3	75.1 ± 1.4	13.5 ± 1.9
SO7–360A	70.2 ± 1.1	8.6 ± 1.7
SO2	59.6 ± 0.8	
SO2–Phen-DC3	61.3 ± 2.2	1.7 ± 2.3
SO2-360A	60.6 ± 0.8	1.0 ± 1.1
SO8^b^	58.0 ± 1.0	
SO8–Phen-DC3	72.2 ± 1.2	14.2 ± 1.6
SO8–360A	69.4 ± 1.2	11.4 ± 1.6

The reported values are the average ± s.d. of three repeats.

^a^Δ*T*_m_ represents the difference in thermal melting temperature, Δ*T*_m_= |*T*_m(DNA–ligand)_ − *T*_m(DNA)_|.

^b^ *T*_m_ represents the global *T*_m_ owing to the polymorphic nature of SO8.

### Nuclear magnetic resonance spectroscopy

NMR data were acquired on 600, 850, and 950 MHz Bruker Avance NEO spectrometers (Bruker Corporation, Billerica, USA) equipped with a quadruple (600 MHz) or triple-resonance (850 and 950 MHz) inverse cryogenic probe. Measurements were conducted at 298.2 K (25°C) in either 95% H_2_O/5% ^2^H_2_O (v/v) or 99.94% ^2^H_2_O (v/v). DNA sample concentrations ranged from 0.1 to 0.5 mM. Water suppression was achieved using the jump-and-return, excitation sculpting, or gradient-tailored excitation (WATERGATE) techniques [43–45]. The imino and aromatic resonances of guanines in each complex were unambiguously assigned using ^15^N- and ^13^C-edited HSQC NMR spectra [46]. For further assignment of exchangeable and non-exchangeable protons, 2D NOESY (τ_m_= 100, 200, 300, 400, 500, and 600 ms) and TOCSY NMR experiments (with DIPSI-2 isotropic mixing block and τ_m_= 40, 60, and 80 ms) were performed on both complexes. Additionally, 2D ^1^H-^13^C HSQC, EASY-ROESY (τ_m_ = 200 ms, spinlock angle = 50°), and DQF-COSY NMR spectra with excitation sculpting solvent suppression were acquired for isotopically unlabeled DNA–ligand complexes in 99.94% ^2^H_2_O [47]. Spectral referencing in the proton dimension was performed using 3-(trimethylsilyl)-1-propane sulfonic acid-d6 sodium salt (TMSP) (CAS Registry Number: 24493-21-8) (MERCK, Czech Republic) with its methyl proton set at δ = 0.0 ppm. Indirect referencing was applied for the ^13^C, ^15^N, and ^19^F dimensions [48]. All spectra were processed using Topspin 4.13 (Bruker Biospin, Germany) and analyzed with NMRFAM-SPARKY 1.414 (UW Madison, USA) [49].

### Structure calculations and restraints

Intra- and intermolecular NOE distance restraints between QDH (SO7) and individual ligands were derived from the 2D NOESY spectra (τ_m_ = 100, 200, and 300 ms) recorded in 95% H_2_O/5% ^2^H_2_O (v/v). The averaged volume of the H5–H6 NOE correlation of cytosine was used as a reference and assigned to a distance of 2.45 Å. Based on the reference, the remaining NOE cross-peaks were classified as strong (1.8−3.6 Å), medium (2.6−5.0 Å), and weak (3.5−6.5 Å). Hoogsteen hydrogen-bonded guanines, forming three G-quartet planes in each complex, were restrained using 24 distance restraints (1.9–2.1 Å for N7-H21 and 1.7–1.9 Å for O6-H1 acceptor–donor pairs, respectively). In the duplex stem-loop, Watson-Crick hydrogen-bonded guanines and cytosines were restrained using nine distance restraints (1.8–2.0 Å for O2-H22, N3-H1, and O6-H41 acceptor–donor pairs, respectively). Thirty-six and nine planarity restraints were used to keep the alignment of three G-quartets and three G-C base pairs in each complex, respectively. Torsion angles χ along the glycosidic bond of QDH were restrained to 60° ± 35° for residues in *syn* glycosidic conformation (namely G1, G3, G7, G20, G21, and G25) based on their strong intra-residual H8–H1' NOE correlations. Other guanines, adenines, and thymine residues were assigned to occupy *anti* conformations, with torsion angles restrained to 240° ± 40° for guanines and adenines and 240° ± 70° for thymines. For each complex, 52 chirality restraints were applied using AMBER tools to prevent chirality flips during simulated annealing (SA) cycles. Structural calculations were performed using Amber 22 software with the Born implicit solvent model [50, 51]. Force field parameters were derived from OL15 [52–56] and the Generalized Amber force field (GAFF2) [57], with partial charges and force field parameters for Phen-DC3 and 360A obtained as described elsewhere [25, 29, 35]. The lowest-energy structure of SO7 (PDB accession code 7CV3, model 1, [36]) was used as an initial template for NMR-guided docking of Phen-DC3 and 360A in the HADDOCK 2.4 web server [58, 59]. The ligand-binding pocket was defined by the Q-D interface (G5, G9, C10, G19, G20, and G27) of SO7, as evident from the chemical shift perturbation and DNA-ligand NOE connectivities. Output files from Cluster 1 of the HADDOCK were used as initial input structure files in the first round of SA protocol, guided by NMR data. SA was conducted using *pmemd.CUDA* module in AMBER 22 [50], with non-bonded interactions cutoffs set to 999 Å. Simulation employed 0.5 fs integration steps, while the SHAKE algorithm for hydrogen atoms with a tolerance of 0.0005 Å for bond lengths and angles was used [60]. The 1000-ps long SA protocol was as follows: in the first 200 ps, the temperature was raised from 300 to 1000 K, held at 1000 K for 300 ps, reduced to 0 K in the next 400 ps, and maintained at 0 K for the final 100 ps. Planarity restraints were excluded during the last 200 ps. The weights of the force constants for restraints increased linearly from 0.1 to 1.0 in the first 500 ps and remained constant thereafter. Restraint force constants were set at 20 kcal × mol^−1^× Å^−2^ for distance and planarity, 50 kcal × mol^−1^× Å^−2^ for hydrogen bonding, and 200 kcal × mol^−1^× rad^−2^ and 100 kcal × mol^−1^× rad^−2^ for torsion angles (χ) and chirality, respectively. In each SA cycle, 200 structures were generated using random initial velocities with the Generalized Born implicit solvent model and Langevin thermostat with an offset of 0.13 for non-bonded interactions [51]. Ten representative structures for each ligand were selected based on the lowest energy and minimal restraint violations from the final SA round. These structures were refined using 10 000 steps of steepest descent energy minimization, yielding the lowest energy conformations for the SO7–Phen-DC3 and SO7–360A complexes. Final structures were analyzed and visualized using UCSF ChimeraX 1.8 and WEB 3DNA 2.0 suite [61, 62]. The NMR-derived coordinates for the SO7–Phen-DC3 and SO7–360A complexes were deposited in the Protein Data Bank (PDB) under accession codes 9GVI and 9GVV, respectively. Associated chemical shift data were deposited in the Biological Magnetic Resonance Data Bank (BMRB) under accession codes 34960 and 34961, respectively (Supplementary Tables S3 and S4).

### Molecular dynamics

#### System preparation

The first five NMR models of both DNA–ligand complexes were subjected to standard explicit solvent MD simulations. The models were solvated in a truncated octahedral box of SPC/E waters with a distance of at least 10 Å between the solute and box border [63]. The systems were neutralized by K^+^, two of which were placed in the G-quadruplex channel between the G-quartets [64]. An additional 50 mM KCl was added to mimic the ionic conditions of the experiment. DNA was described by the OL21 force field [52–55, 65], whereas the ligands used the GAFF2 force field [57].

#### Standard MD simulations

Electrostatics were treated using the particle-mesh Ewald method with a non-bonded 9 Å cutoff [66–68]. We applied the SHAKE and SETTLE algorithms to the solute and solvent, respectively, in addition to the hydrogen mass-repartitioning approach, which allowed us to use an integration time step of 4 fs [69–71]. The systems were first heated to 300 K and equilibrated in an alternating sequence of minimization and restrained MD simulations as previously described [72]. The equilibrated structures are available in the Supplementary Data. Standard production dynamics were then initiated. The temperature and pressure were constant at 300 K and 1 atm, respectively, using a Langevin thermostat and Monte Carlo barostat. The trajectories were calculated using the CUDA-accelerated pmemd module of AMBER18 [73, 74]. Each independent simulation was run for 5 μs, thus obtaining a total of 25 μs for each DNA–ligand complex.

#### Metadynamics

Because standard MD simulations showed that the significant ligand dynamics in the system were related to its axial rotation, we decided to utilize well-tempered metadynamics (wt-metaD) to accelerate sampling in this direction [75]. The collective coordinate, hereafter called angular position, used to enhance the rotation sampling, was defined as a pseudotorsion angle with two defining points related to the ligand and two points to the adjacent G-quartet. These four points are geometric centers of the following groups of atoms: (i) C2′ and O4′ atoms of G9 and G20 (two guanines of the G-quartet adjacent to the duplex); (ii) four O6 atoms of guanines constituting the G-quartet; (iii) three atoms from each of the aromatic moieties I (atoms a′, b, K and c, c′, J in Phen-DC3 and 360A, respectively), II (B, C, f), and III (B′, C′, f′) of the ligands; and (iv) three atoms of moiety I (atoms a′, b, K and c, c′, J) (Fig. 1A). The four points were selected such that the angular position of 0° corresponds to the ligand’s middle ring (moiety I) positioned between the stem-loop’s G-quartet and adjacent base pair. As starting points for the wt-metaD simulations, we used the five equilibrated structures described previously and ran them under the same conditions as in the standard simulations. The hydrogen-mass repartitioning scheme could not be used; therefore, the time step was set to 2 fs. The exact settings of the parameters governing the wt-metaD can be found in the Supplementary Data. The calculations were performed using PLUMED 2.7.4 integrated into AMBER20 [76, 77]. Each independent wt-metaD simulation was 2 μs long; therefore, we accumulated 10 μs for each DNA–ligand complex.

#### Simulation analyses

The obtained trajectories were post-processed in the cpptraj module of AMBER and were visually inspected using VMD. The wt-metaD data were analyzed using PLUMED 2.7.4. The obtained trajectories were post-processed using the cpptraj module of AMBER and visually inspected using VMD [78, 79]. Wt-metaD data were analyzed using PLUMED 2.7.4 [76].

### In-cell NMR methodology in *Xenopus laevis* oocytes

Stock solutions for in-cell NMR were prepared by mixing prefolded DNA (SO8-F) without/with Phen-DC3 or 360A at a 1:1 molar ratio and ∼1.5–2.0 mM concentration of the complex in NMR buffer (without ^2^H_2_O and TMSP). The formation of an appropriate DNA–ligand complex was validated by monitoring 1D ^1^H and ^19^F NMR spectra of stock samples diluted to ∼100 μM concentration *in vitro*. The 1D ^19^F NMR experiments were performed with a 100 ppm spectral width, carrier frequency set to −195 ppm, and 2 s relaxation delay while using composite pulses to suppress the background signal from the probe. Depending on the signal-to-noise (S/N) ratio, the number of transients varied between 256 and 2048, with 32 dummy scans for each NMR spectrum. All ^19^F NMR spectra were processed with an exponential window function using line broadening of 20 and 100 Hz for *in vitro* and in-cell conditions, respectively. The presence of DMSO at <8%, alone or in combination with the ligand, did not elicit any discernible effect *in vitro* and control sets of *X. laevis* oocytes, compared to untreated oocytes (data not shown). In each experiment, ∼300 *X. laevis* oocytes of stage VI (first-grade quality, min. diameter 1.1 mm; purchased from EcoCyte Bioscience, Dortmund, Germany) were briefly incubated at room temperature in a Petri dish containing Ori-Ca^2+^ buffer (5 mM HEPES, pH 7.6, 110 mM NaCl, 5 mM KCl, 2 mM CaCl_2_, and 1 mM MgCl_2_) supplemented with 10% Ficoll (Cytiva). Subsequently, all the oocytes were individually placed in three 96-well plates (Thermo Scientific) in Ori-Ca^2+^ buffer before sample injection. For each in-cell NMR experiment, ∼70 nl aliquots of the stock solution (2.0 mM SO8-F or 1.5 mM SO8-F-Phen-DC3 and 360A 1:1 complexes) were injected into the oocytes for ∼1 h using the automated injector Robocyte2 (MultiChannel Systems, Reutlingen, Germany) according to the manufacturer’s instructions. The injected oocytes were then recovered for 1 h, and ∼250 oocytes were collected and transferred to a Shigemi NMR tube without a plunger (Shigemi Co., Tokyo, Japan) (intracellular concentration ∼100–140 μM). The NMR tube was filled with ∼2 ml Ori-Ca^2+^ buffer containing 10% Ficoll and 10% ^2^H_2_O. These oocytes were subsequently investigated by ^19^F NMR spectroscopy. In addition, we utilized 10–15 injected oocytes to perform viability and maturation tests to verify oocyte integrity. This was achieved by adding 1 μM progesterone hormone (Merck) dissolved in DMSO to the Ori-Ca^2+^ buffer with 10% Ficoll overnight. As a negative control, 5% (v/v) pure DMSO was added to the buffer for 10–15 injected oocytes. Finally, these oocytes were incubated overnight at 16–18°C and observed using the Leica S9i stereoscope (Leica, Germany) and LAX software (version 1.4.5).

## Results

### Phen-DC3 and 360A selectively bind to the hybrid topology of QDH derived from the *PIM1* gene promoter

To investigate the topology-specific binding of Phen-DC3 and 360A to QDHs, three sequences derived from the *PIM1* gene promoter were used in this study: hybrid QDH-forming SO7, SO2 with antiparallel G-strand orientation, and polymorphic SO8, which can simultaneously adopt both conformations at the same time (Fig. 1B and Table 1) [36]. It needs to be stressed that SO8, SO7, and SO2 are distinct oligonucleotides, differing in length and sequence, and therefore exhibit different conformational properties. Despite sequence and length differences, the two conformations (hybrid and antiparallel) adopted by SO8 closely resemble the single conformations formed by SO7 and SO2, respectively (Fig. 1B and Table 1). Consequently, the moderate differences in *T*_m_ values observed for free SO8 can be attributed to sequence differences when compared to free SO7 and SO2, respectively (Table 1). Specifically, in the antiparallel conformation, SO8 contains a 5′-GC overhang that may reduce its thermal stability relative to SO2, in which the 5′-G forms a more stable GC base pair with the 3′-C. Similarly, in hybrid conformation, SO8 contains an extra 3′-terminal cytosine, which could affect its stability compared to SO7.

The ^1^H NMR spectrum of free SO7 displayed signals consistent with its known folding topology, including 12 well-resolved imino signals between 10.0−12.0 ppm and three signals between 12.5−14.0 ppm corresponding to guanines engaged in G-quartet and stem-loop formation, respectively (Fig. 1C) [36]. Upon titration of SO7 with Phen-DC3, signals corresponding to free SO7 gradually decreased and completely disappeared at the 1:1 SO7:Phen-DC3 ratio. At the same time, a new set of signals appeared, indicating the formation of a well-defined complex (Fig. 1C and Supplementary Fig. S1). Excess Phen-DC3 caused line broadening, suggesting non-specific binding (Supplementary Fig. S1). Similarly, titration of SO7 with 360A led to the formation of a well-defined 1:1 SO7–360A complex with signal broadening and decreased intensities at higher ligand concentrations (Fig. 1C and Supplementary Fig. S2). CD spectra revealed that the hybrid conformation of SO7 was retained upon ligand binding, with minimal shifts at ∼265 and ∼290 nm, respectively (Supplementary Fig. S3A). A decrease in the negative peak at ∼245 nm upon ligand binding indicates affected interactions between the G-tetrads and the duplex stem-loop. Ligand binding was also reflected in increased melting temperatures by ∼13.5°C (Phen-DC3) and ∼8.6°C (360A) for the 1:1 complexes, respectively (Supplementary Fig. S3B and Table 2).

In contrast, NMR and CD data indicated that Phen-DC3 and 360A showed minimal binding to SO2. Titration of Phen-DC3 and 360A to SO2 caused no significant shifts in the imino resonances up to a 1:1 DNA: ligand ratio (Figs 1C and Supplementary Fig. S4). At a 1:1 SO2:Phen-DC3 ratio, several minor signals (<10% population) emerged between 10.0 and 14.0 ppm, which became more pronounced at higher ratios (1:2 and 1:3), suggesting the formation of different, low-population ligand-bound species (Supplementary Fig. S4A). Overlapping, broad signals emerged at a 1:2 SO2:360A ratio, which intensified at a 1:3 ratio, consistent with the formation of various non-specific complexes (Supplementary Fig. S4B). Even after extended incubation (up to 14 days) of SO2 with Phen-DC3 or 360A at equimolar ratios, NMR spectra revealed only a minor population of the ligand-bound form, with the free antiparallel QDH state remaining dominant (Supplementary Fig. S5), demonstrating that the antiparallel *PIM1* QDH is relatively resistant to refolding induced by Phen-DC3 and 360A.

The CD profiles of SO2 in the presence of the equimolar amount of Phen-DC3 and 360A were identical to those of free SO2, suggesting no or minimal binding at this titration point (Supplementary Fig. S3A). Following CD and NMR data, the addition of Phen-DC3 and 360A to SO2 showed a negligible effect on the *T*_m_ of SO2 (Supplementary Fig. S3B and Table 2).

The imino region of the ^1^H NMR spectrum of SO8, which adopts both hybrid and antiparallel QDH structures in solution, showed two distinct sets of signals highly reminiscent of free SO7 and SO2 constructs (Fig. 1C). Upon titration with Phen-DC3, signals from hybrid QDH gradually decreased and disappeared at a 1:1 SO8:Phen-DC3 ratio, while a new set of signals closely resembling the SO7–Phen-DC3 complex emerged (Fig. 1C and Supplementary Fig. S6). Signals from antiparallel QDH diminished upon adding Phen-DC3 without chemical shift perturbations and disappeared completely at the 1:1 SO8:Phen-DC3 ratio (Supplementary Fig. S6). At 1:2 SO8:Phen-DC3, additional low-population complex(es) formed, indicated by new, less intense imino signals. Non-specific binding and signal broadening were observed at a 1:3 SO8:Phen-DC3 ratio (Supplementary Fig. S6). Similarly, titration of SO8 with 360A to a 1:1 ratio resulted in the disappearance of free QDH imino signals and the emergence of signals identical to SO7–360A complex (Figs 1C and Supplementary Fig. S7). With increasing SO8:360A ratios, the signals broadened progressively and coalesced into broad humps at a 1:3 ratio (Supplementary Fig. S7). CD spectra of 1:1 SO8-Phen-DC3 and SO8-360A matched those of SO7–Phen-DC3 and SO7–360A, respectively (Supplementary Fig. S3A). *T*_m_ values increased by 14.2°C and 11.4°C for SO8 upon equimolar addition of Phen-DC3 and 360A, respectively (Supplementary Fig. S3B). Additionally, the 2D NOESY spectra of SO8-Phen-DC3/360A 1:1 complexes closely resembled those of the SO7–Phen-DC3/360A 1:1 complexes (Supplementary Fig. S8). Their similar NOESY fingerprints indicate that the global folding of the SO8-ligand complexes is consistent with that of the SO7-ligand complexes.

To further explore the binding preference of Phen-DC3 and 360A for the co-existing QDH populations, a ^1^H NMR competition experiment was conducted using a 1:1 mixture of SO7 and SO2, which showed distinct and well-resolved imino peaks corresponding to the two QDH topologies (Supplementary Fig. S9). Upon ligand addition, the imino peaks of free SO7 disappeared, with new peaks emerging that corresponded to the ligand-bound state. In contrast, the imino peaks of SO2 remained unperturbed and overlapped with those of the free SO2. The ligand-bound state observed in the mixed oligonucleotide sample matched the peaks of the SO7–ligand complexes. These findings demonstrate a strong conformational preference of Phen-DC3 and 360A for the hybrid QDH, forming SO7–Phen-DC3 and SO7–360A 1:1 complexes, respectively.

We performed additional CD melting experiments to evaluate ligand binding at 1:2 and 1:3 DNA:ligand ratios (Supplementary Figs S10 and S11; Supplementary Tables S1 and S2). At a 1:2 DNA:ligand ratio, all constructs showed increased thermal stability compared to the 1:1, suggesting optimal occupancy of high-affinity sites and cooperative binding at additional sites, reinforcing native folding without disrupting the QDH structure. However, at a 1:3 DNA:ligand ratio, *T*_m_ values for SO7 and SO8 decreased noticeably, indicating that excess ligand may interfere with stabilizing interactions, bind nonspecifically, or even induce partial unfolding of QDH. This is supported by ^1^H NMR titrations of SO7 and SO8 constructs with Phen-DC3 and 360A, where ligand excess (≥2 equivalents) led to multiple DNA–complexes, consistent with non-specific binding and potential QDH unfolding (Supplementary Figs S1, S2, S6, and S7). In contrast, SO2 showed increased *T*_m_ values even at a 1:3 ratio, likely due to non-specific surface binding of both ligands or their engagement at multiple low-affinity sites that modestly stabilize the structure without forming well-defined complexes. Our findings highlight that higher ligand concentrations can enhance thermal stability *via* multivalent binding. However, they may also destabilize the structure through non-specific interactions or disruption of the native QDH fold. Therefore, Δ*T*_m_ values under excess ligand conditions should be interpreted cautiously, as they may not necessarily reflect specific or structurally defined binding events.

NMR and CD melting data acquired at a 1:1 (DNA: ligand) ratio showed that Phen-DC3 and 360A form stable complexes with SO7, while minimal ligand binding and non-specific complexes were observed for SO2. For SO8, which adopts both hybrid and antiparallel QDH topologies, both ligands selectively stabilized the hybrid form, with NMR patterns resembling SO7–ligand complexes. ^1^H NMR competition experiment revealed that Phen-DC3 and 360A directly bind to the hybrid conformation (SO7), shifting the equilibrium toward the hybrid QDH–ligand complex. These findings highlight the ligands’ specificity for hybrid QDHs and the need for further structural insights into SO7–ligand complexes to better understand ligand selectivity.

### Intermolecular NOE contacts localize ligand binding to the Q-D junction

**Figure 2.**
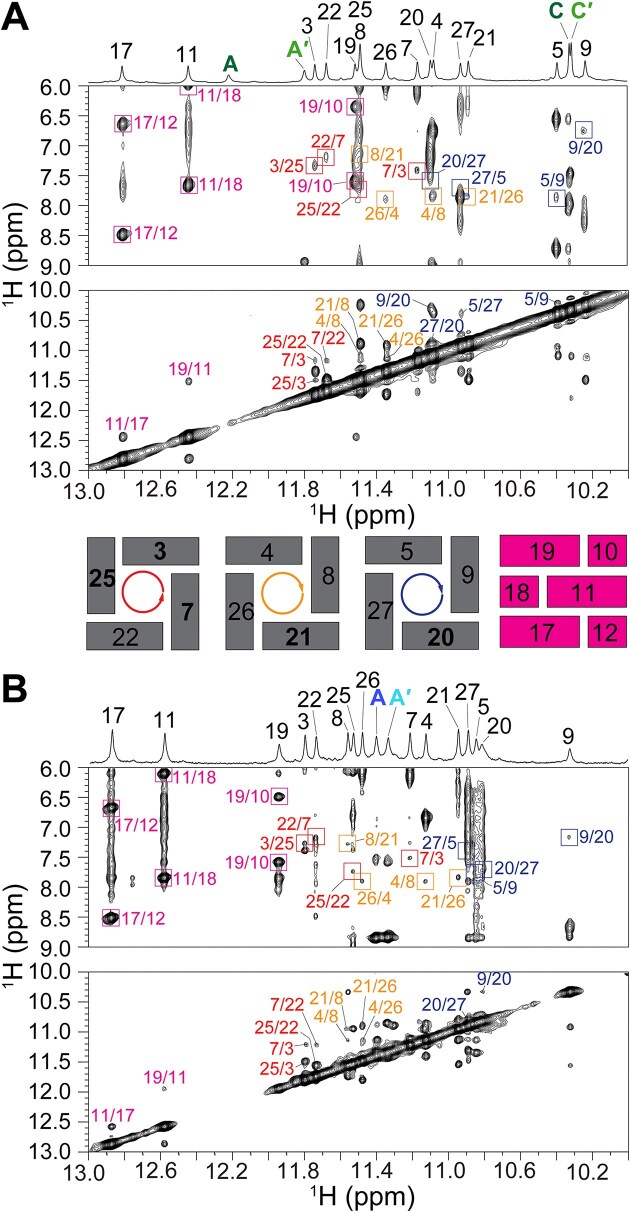
The imino regions of 1D ^1^H NMR spectra of (**A**) SO7–Phen-DC3 and (**B**) SO7–360A 1:1 complexes with corresponding imino-aromatic and imino-imino regions of 2D NOESY spectra (τ_m_ = 300 ms). Cross peaks labeled with red, orange, and dark blue rectangles correspond to G3•G25•G22•G7, G4•G8•G21•G26, and G5•G9•G20•G27 quartets, respectively. Cross-peaks labeled with magenta correspond to NOE connections between G-C base pair-forming residues. Ligand imino signals are marked according to the labels in Fig. 1A.

The 2D NOESY spectra of SO7–Phen-DC3 and SO7–360A 1:1 complexes confirmed that SO7 retains its three-quartet hybrid topology, with G-quartets formed by residues G3•G25•G22•G7, G4•G8•G21•G26, and G5•G9•G20•G27 (Fig. 2; for unambiguous assignment of imino and aromatic protons see Supplementary Fig. S12; for detailed description of assignment see Supplementary Information text, Supplementary Figs S12–S15 and Supplementary Tables S3 and S4). Intense intraresidual NOE cross-peaks in the anomeric-aromatic region of NOESY spectra and downfield chemical shift of C8 carbon atoms indicated *syn* glycosidic conformations for G1, G3, G7, G20, G21, and G27 (Supplementary Figs S13 and S14) [80]. Watson-Crick base pairs G19–C10, G11–C18, and G17–C12 in the duplex stem-loop were preserved in both complexes (Fig. 2), although the NOE network connecting the 3′ G-quartet (specifically G9 H8/H1 and G20 H8/H1) to the stem-loop (C10 H5/H6 and G19 H1/H8) was disrupted. Significant upfield shifts of G5, G9, G19, and G27 H1 protons, in comparison to free SO7, suggest that the binding pocket of Phen-DC3 and 360A is located in close proximity to these residues (Supplementary Fig. S16 and Supplementary Tables S3 and S4).

**Figure 3.**
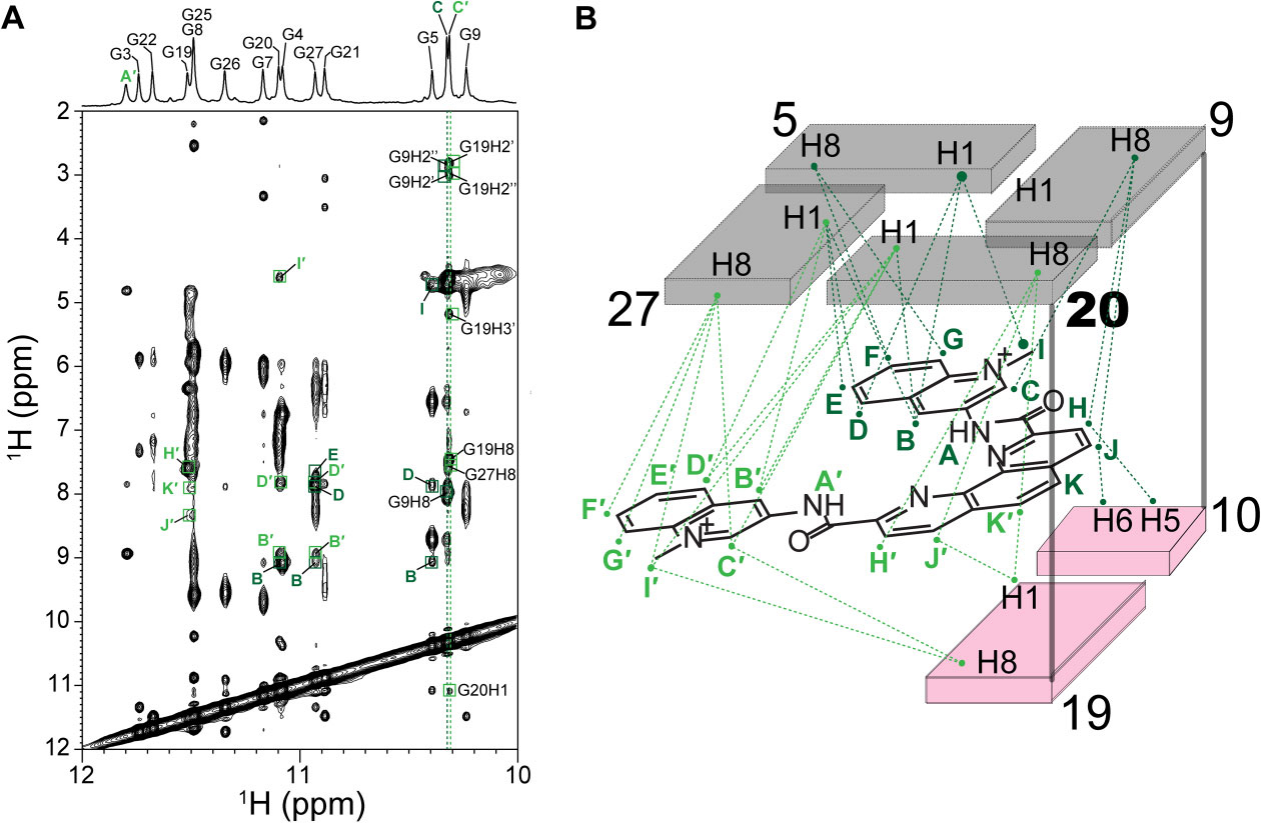
Intermolecular NOE contacts positioning Phen-DC3 between G5•G9•G20•G27 quartet, and G19–C10 base pair at the Q-D junction. (**A**) The region of the NOESY spectrum (τ_m_= 300 ms) shows intermolecular NOE cross-peaks between the imino protons of SO7 and Phen-DC3. NOESY spectral region showing intermolecular NOE interactions between other protons of SO7 and Phen-DC3 is presented in Supplementary Fig. S19A. (**B**) Schematic depiction of key intermolecular interactions within the SO7–Phen-DC3 1:1 complex. NOESY spectrum was obtained in 20 mM potassium phosphate buffer, pH 7.1, 20 mM KCl at 298.2 K with 0.5 mM SO7 and Phen-DC3 concentrations.

**Figure 4.**
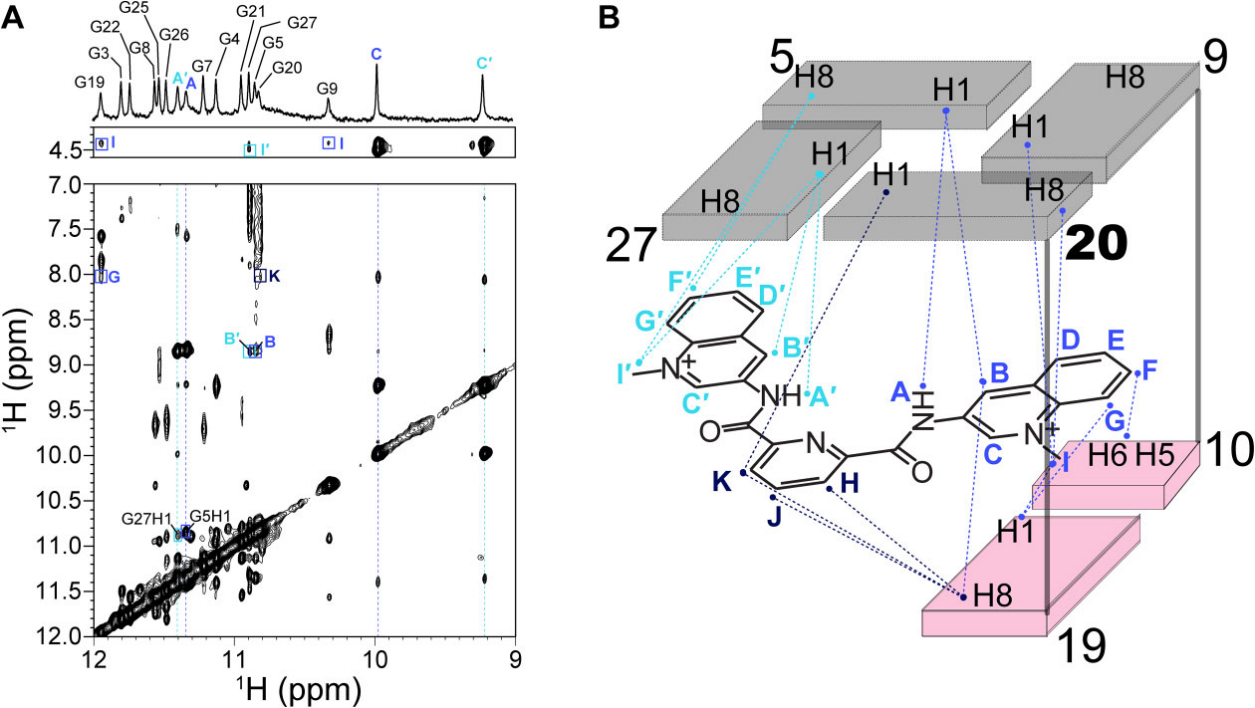
Intermolecular NOE contacts positioning 360A between G5•G9•G20•G27 quartet and G19–C10 base pair at the Q-D junction. (**A**) The region of the NOESY spectrum (τ_m_= 300 ms) shows intermolecular NOE cross-peaks between the imino protons of SO7 and 360A. NOESY spectral region showing intermolecular NOE interactions between other protons of SO7 and 360A is presented in Supplementary Fig. S19B. (**B**) Schematic presentation of key intermolecular interactions within the SO7–360A 1:1 complex. NOESY spectrum was obtained in 20 mM potassium phosphate buffer, pH 7.1, 20 mM KCl at 298.2 K with 0.5 mM SO7 and 360A concentrations.

The imino, aromatic, and methyl protons of the Phen-DC3 and 360A in the complex with SO7 were assigned using a set of 2D NMR spectra, as described previously (Supplementary Figs S17 and S18; Supplementary Table S5) [27, 29, 35]. The precise positions of both ligands in the binding pocket of SO7 were supported by 73 and 40 intermolecular NOE cross-peaks in the SO7–Phen-DC3 and SO7–360A complexes, respectively (Figs 3 and 4, Supplementary Fig. S19, and Supplementary Tables S6 and S7). These NOEs show connections between the G5•G9•G20•G27 quartet, G19–C10 base pair, and different parts of Phen-DC3 and 360A molecules, respectively, suggesting that the binding pocket of both ligands resides at the Q-D junction of SO7 (Figs 3 and 4). Additionally, no intermolecular NOE contacts were observed between the ligand (Phen-DC3 or 360A) and the 5′ or central G-quartet, thus excluding potential intercalation of the ligands between G-quartets or 5′-quartet stacking binding modes.

Several NOE connections between residues G9, C10, G19, and G20 of SO7, and H, J, K, H′, J′, and K′ atoms of Phen-DC3, indicate that the ligand in SO7–Phen-DC3 1:1 complex is with its phenanthroline moiety positioned between G19–C10 base pair and G9–G20 edge of the G-quartet (Fig. 3, Supplementary Fig. S19A, and Supplementary Table S6). Positions of both quinolinium rings of Phen-DC3 are well-defined inside the binding pocket: while B, C, D, E, F, G, and I of the first quinolinium group show NOE cross-peaks to all four guanines from the G5•G9•G20•G27 quartet, NOEs between G20, G27 and G19 and atoms B′, C′, D′, E′, F′, G′ and I′ from the second ring were observed (Fig. 3, Supplementary Fig. S19A, and Supplementary Table S6). Additionally, Phen-DC3 demonstrates no exchange cross-peaks in the EASY-ROESY (τ_m_= 200 ms) in the complex, indicating that the position of Phen-DC3 inside the binding pocket is rigid without fluxional dynamics (Supplementary Fig. S20A).

On the other hand, intermolecular NOE connections between SO7 and 360A show that one of the quinolinium rings of 360A is stacked below the G9–G20 edge of the G5•G9•G20•G27 quartet and covered by the G19–C10 base pair. In contrast, the other quinolinium ring shows NOE cross-peaks to residues G5 and G27 across the same G-quartet (Fig. 4, Supplementary Fig. S19B, and Supplementary Table S7). This places the pyridine moiety between G19 and G20, as confirmed by NOEs between these two residues and H, J, and K atoms of 360A. Compared to the position of Phen-DC3 inside the binding pocket, 360A is less defined, as indicated by the lower number of intermolecular NOEs. In line with this indication, we observed exchange cross-peaks in EASY-ROESY NMR spectra between symmetry-related protons (C and C′) of 360A, suggesting chemical exchange that can arise from the flipping motion of the quinolinium rings inside the binding pocket (Supplementary Fig. S20B) [47]. This contrasting behavior is explained later in the molecular dynamics simulations section. Altogether, intermolecular NOE contacts and chemical shift perturbation data unequivocally demonstrate the binding of Phen-DC3 and 360A at the Q-D junction of SO7.

### Three-dimensional high-resolution structures of the SO7–Phen-DC3 and SO7–360A 1:1 complexes

**Figure 5.**
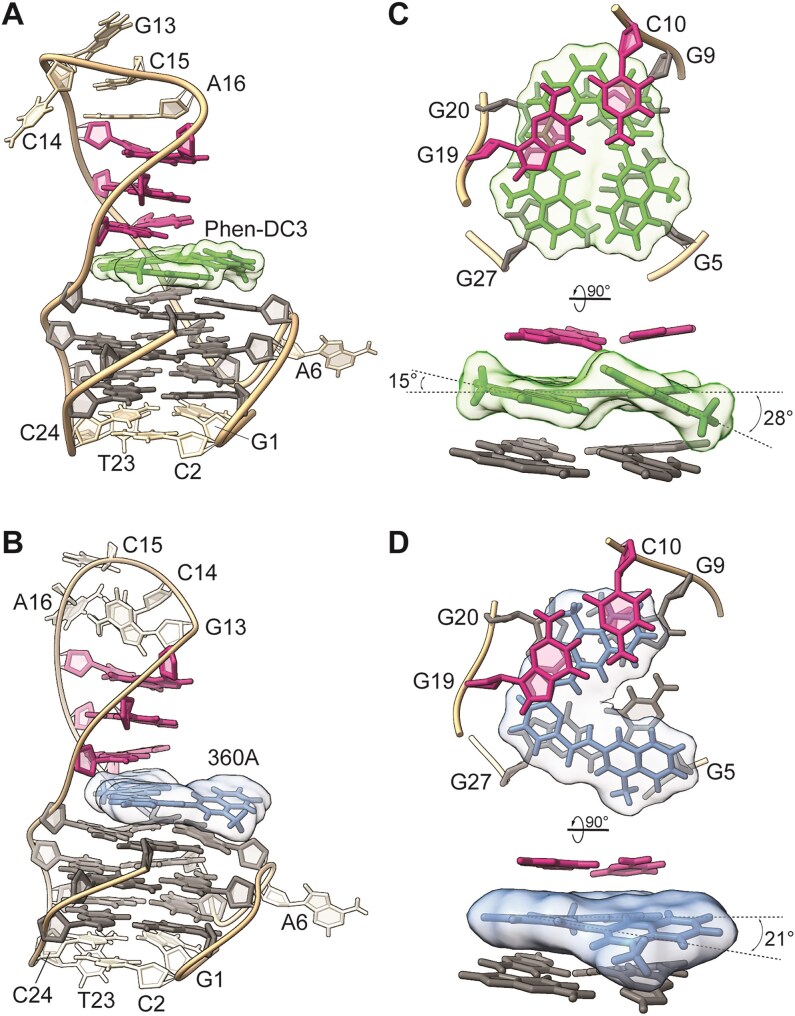
Lowest-energy NMR solution structures of (**A**) SO7–Phen-DC3 (PDB: 9GVI) and (**B**) SO7–360A (PDB: 9GVV) 1:1 complexes. Bird’s- and side-view into the binding pocket of (**C**) SO7–Phen-DC3 and (**D**) SO7–360A complexes at the Q-D junctions. G-quartet-forming guanines and residues forming G–C base pairs in the stem-loop are marked gray and magenta, respectively. Phen-DC3 is colored green, while 360A is blue. All the other residues are depicted in light brown.

The NMR structures of the SO7–Phen-DC3 and SO7–360A 1:1 complexes were determined by incorporating 527 (Phen-DC3) and 475 (360A) NOE-based distance restraints. Additionally, 27 torsion angles, 33 hydrogen bonds, and 42 planarity restraints were used for structure calculations of the complexes (Supplementary Tables S8 and S9). The convergence of the 10 lowest-energy structures was characterized by average pairwise RMSD values (all heavy atoms) of 1.51 ± 0.57 and 1.82 ± 0.71 Å for SO7–Phen-DC3 and SO7–360A, respectively. High-resolution structures of SO7–Phen-DC3 and SO7–360A 1:1 complexes revealed that the binding of Phen-DC3 and 360A did not influence the overall fold of SO7 (Fig. 5 and Supplementary Fig. S21). A single-nucleotide propeller loop, a two-nucleotide lateral loop, and a 10-nucleotide middle duplex stem-loop connect the four G-columns. The coaxial duplex stem-loop spans the wide groove and is stabilized by G19–C10, G11–C18, and G17–C12 Watson-Crick base pairs. While G–C base pairs are well-defined, residues G13–C14–C15–A16, which cap the stem-loop, are flexible and exposed to the solvent (Fig. 5 and Supplementary Figs S21-S22). Of these residues, only A16 exhibits a somewhat limited degree of motion, appearing above the G17–C12 base pair in all models and, in some cases, even stacking directly on the base pair supported by long-range NOE contacts between C12 and A16. A6 from the single-nucleotide propeller loop that bridges the medium groove is exposed to the solvent in both complexes, while the residues T23 and C24 from the third, narrow groove-spanning lateral loop reside below the 5′-quartet. At the same time, residues G1 and C2 from 5′-overhang are stacked below the same G-quartet (Supplementary Fig. S22). This spatial arrangement brings G1, C2, and C24 into close proximity, and some calculated models suggest the formation of a GCC triad in both complexes. However, experimental data do not provide direct evidence supporting hydrogen bonding within the proposed GCC triad.

Overall, the structures of SO7 in both complexes are highly similar, sharing key structural features and hydrogen-bonding patterns. This consistency suggests that the binding pocket at the Q-D junction is well-defined and rigid, requiring the ligand to orient according to the available binding space. The most substantial structural perturbations of the SO7 in both complexes compared to the free SO7 occur near the ligand binding site and include changes in the rise between successive planes of the Q-D junction and widths of the narrow groove defined by the G20–G22 and G25–G27 G-strands. The binding of the Phen-DC3 and 360A in the Q-D junction increases the rise between the G9–G20 and C10–G19 steps compared to the free SO7 by 2.4 and 2.8 Å, respectively (Supplementary Fig. S22). Additionally, the width of the narrow groove at the G20–G27 G-quartet edge of the Q-D junction increases by 3.3 Å and 4.4 Å for SO7–Phen-DC3 and SO7–360A complexes in comparison to free SO7, respectively (8.9 ± 0.2 Å and 10.0 ± 0.7 Å for SO7–Phen-DC3 and SO7–360A, respectively, in comparison to 5.6 ± 0.2 Å for SO7) (Supplementary Fig. S23A–C). On average, narrow groove width, defined by G20–G22 and G25–G27 G-strands, increases by 3.6 Å for Phen-DC3 and 360A. Notably, no significant change in the widths of other grooves was observed upon ligand binding.

Ligand binding in the Q-D junction also affects more distant regions of the SO7. In contrast to SO7 in complex with Phen-DC3 or 360A, where C24 was stacked below 5′ G-quartet and in close proximity to G1 and C2, C24 is oriented away from the G3•G25•G22•G7 quartet in free SO7, giving space to G1 and C2 (Supplementary Fig. S22). Additionally, the P–P distance between base pair-forming C12 and G17 decreased by almost 4 Å in both complexes compared to free SO7, suggesting a more compact arrangement of the residues at the end of the stem-loop (Supplementary Fig. S23D). This might be a consequence of the more rigid position of A16 above the G17–C12 base pair observed in SO7–Phen-DC3 and SO7–360A complexes, which was absent in the free SO7 (Supplementary Fig. S22).

### The binding pocket architecture of the SO7–Phen-DC3 and SO7–360A 1:1 complexes

The position of Phen-DC3 in the binding pocket of SO7 is well-defined (Fig. 5C). The ligand’s rigid planar phenanthroline ring is intercalated into the Q-D junction. The two quinolinium rings are oriented away from the Q-D junction and point towards the G5–G27 edge of the G-quartet. The quinolinium rings are not entirely coplanar; instead, they are tilted for ∼28° and 15° with respect to the phenanthroline moiety (Fig. 5C). The inclination of the quinolinium rings enables optimal stacking of Phen-DC3 on the G5•G9•G20•G27 quartet. The N-methyl groups of Phen-DC3 (I and I′) point towards the medium (G5–G9) and narrow (G20–G27) grooves, facilitating electrostatic interactions with the phosphate backbone of G5–A6 and G19–G20, respectively.

360A also binds to the Q-D junction of SO7, but its relative orientation in the pocket differs (Fig. 5D). While one of the quinolinium rings of 360A occupies the space in the Q-D junction, the pyridine moiety resides in the proximity of G27. The second quinolinium group is more exposed and steered towards G5 on the other side of the Q-D-forming G-quartet. For optimal aromatic stacking, the quinolinium ring sandwiched between the two planes of the Q-D junction shows an almost coplanar orientation with a pyridine ring. On the other hand, the more exposed quinolinium moiety, stacked to G5, is tilted by ∼21° compared to the pyridine ring.

### MD simulations reveal that 360A can occupy two distinct positions within the binding pocket

NMR data suggested that while Phen-DC3 adopts a single binding mode, the position of 360A within the binding pocket can be exchanged between different positions. To rationalize the NMR data, we performed standard unrestrained and enhanced-sampling MD simulations on SO7–Phen-DC3 and SO7–306A complexes (Supplementary Figs S24–S27). Both DNA–ligand complexes remained stable in all simulations. G-quartets and the duplex-stem base pairs remained intact under all conditions, while the G13-C14-C15-A16 tetraloop exhibited significant structural fluctuations, consistent with the increased flexibility observed in NMR-derived conformations. Two K^+^ ions were consistently retained in the ion-binding channels throughout the simulations, and no additional K^+^-binding sites of a significant population were detected.

The ligands intercalated between the G5•G9•G20•G27 quartet and duplex stem-loop but exhibited dynamic behavior. Two types of motion were observed: (i) horizontal sliding, i.e. a mild lateral displacement of the ligand (∼2 Å) on the G-quartet surface, and (ii) axial rotation, i.e. a twisting motion of the ligand. The horizontal movement was similar for both ligands and minimal enough to be disregarded in wt-metaD calculations. In contrast, axial rotation was the primary motion distinguishing Phen-DC3 from 360A.

In the MD simulations, Phen-DC3 predominantly intercalated its phenanthroline moiety into the Q-D junction, exposing its quinolinium rings to water (Supplementary Fig. S25A and C). In contrast, 360A intercalated its quinolinium rings into the same pocket. Due to two quinolinium rings, 360A occupied two positions with roughly similar populations (Supplementary Fig. S25B and D). Wt-metaD enhanced sampling simulations revealed single free-energy minima for Phen-DC3 and two significant free energy minima for 360A, with interconversion between these states observed even in standard MD simulations (Supplementary Figs S26 and S27). We postulate that both ligands seek to bury their largest hydrophobic ring systems within the pocket formed by the G-quartet and adjacent duplex stem-loop base pair (phenanthroline moiety for Phen-DC3 and quinoline rings for 360A, respectively).

### Effect of Q-D junction mutations on ligand binding

The intercalation of the ligand at the Q-D junction disrupts the stacking interactions between the G5•G9•G20•G27 quartet and the adjacent G19–C10 base pair (Fig. 5). This disruption is energetically compensated by the two-side stacking interactions between the ligand and nucleotides at the Q-D junction. To assess how the Q-D junction composition affects the Phen-DC3 and 360A binding specificity at the Q-D interface, we designed a series of SO7-based constructs with varying stem-loop lengths and Q-D junction-facing base pair arrangements. These constructs included variants with two (SO7_2d_) or one (SO7_1d_) base pairs in the stem-loop, a T-T mismatch replacing the G19–C10 base pair (SO7_TT_) or alternative base pairs at the Q-D junction: G10–C19 (SO7_G10-C19_), A10-T19 (SO7_A10-T19_), and T10-A19 (SO7_T10-A19_) (Table 1).

All constructs formed unimolecular QDHs *in vitro*, as evidenced by the sharp and well-resolved imino proton signals characteristic of Hoogsteen and Watson-Crick hydrogen bonding in the ^1^H NMR spectra (Supplementary Figs S28 and S29). Ligand-induced chemical shift perturbations of imino signals from Watson-Crick base pairs in the duplex domain (12.5–14.0 ppm) were observed for all constructs except SO7_TT_ in the presence of the equimolar Phen-DC3 or 360A, indicating ligand binding at the Q-D junction. For SO7_TT_, the imino signals from Watson-Crick base pairs were unaffected by ligand addition. Still, a substantial shift and an increased number of imino signals between 10.0–12.5 ppm suggested the formation of multiple Hoogsteen base-paired complexes (Supplementary Fig. S28C).

The broad NMR signal patterns (10.0–12.5 ppm) of SO7_TT_ and SO7_nd_ (a construct lacking the duplex stem-loop) upon Phen-DC3 or 360A addition indicate that these changes may result from non-specific ligand binding to the G4 domain (Supplementary Fig. S28C and D). These findings demonstrate that the duplex stem-loop, with at least one Watson-Crick base pair directly stacked on the G4 unit, is essential for specific 1:1 binding of Phen-DC3 and 360A at the Q-D junction, while the nature of the base pair is not a determining factor (Supplementary Fig. S29).

### Intracellular effects on *PIM1* QDH conformations and ligand binding

Environmental factors such as ionic strength, molecular crowding, osmolytes, metabolytes, and protein interaction can influence DNA folding, conformational polymorphism, and interactions between DNA and low molecular weight ligands [81–85]. To investigate the conformational dynamics of *PIM1* QDH sequences in cells, we used *X. laevis* oocytes as a eukaryotic model system and employed in-cell NMR spectroscopy [37, 85, 86]. Our goal was to evaluate the populations of polymorphic SO8 in cells and how they are affected by ligand binding. Using established protocols for microinjecting exogenous nucleic acids into oocytes, we injected SO8 and its pre-formed complexes with Phen-DC3 or 360A into stage VI *X. laevis* oocytes and acquired 1D ^1^H NMR spectra [85, 86]. Initial ^1^H NMR spectra of SO8 were unresolved due to significant signal broadening (data not shown). To address this, we designed chemically modified QDH constructs, SO8-F and SO2-F, by replacing G27 with a 2′-deoxy-2′-fluoro-arabinoguanosine (2′-FANA; ^F^G), a sensitive ^19^F NMR probe known to report on DNA structure and ligand binding both *in vitro* and *in vivo* (Table 1) [41, 85].

**Figure 6.**
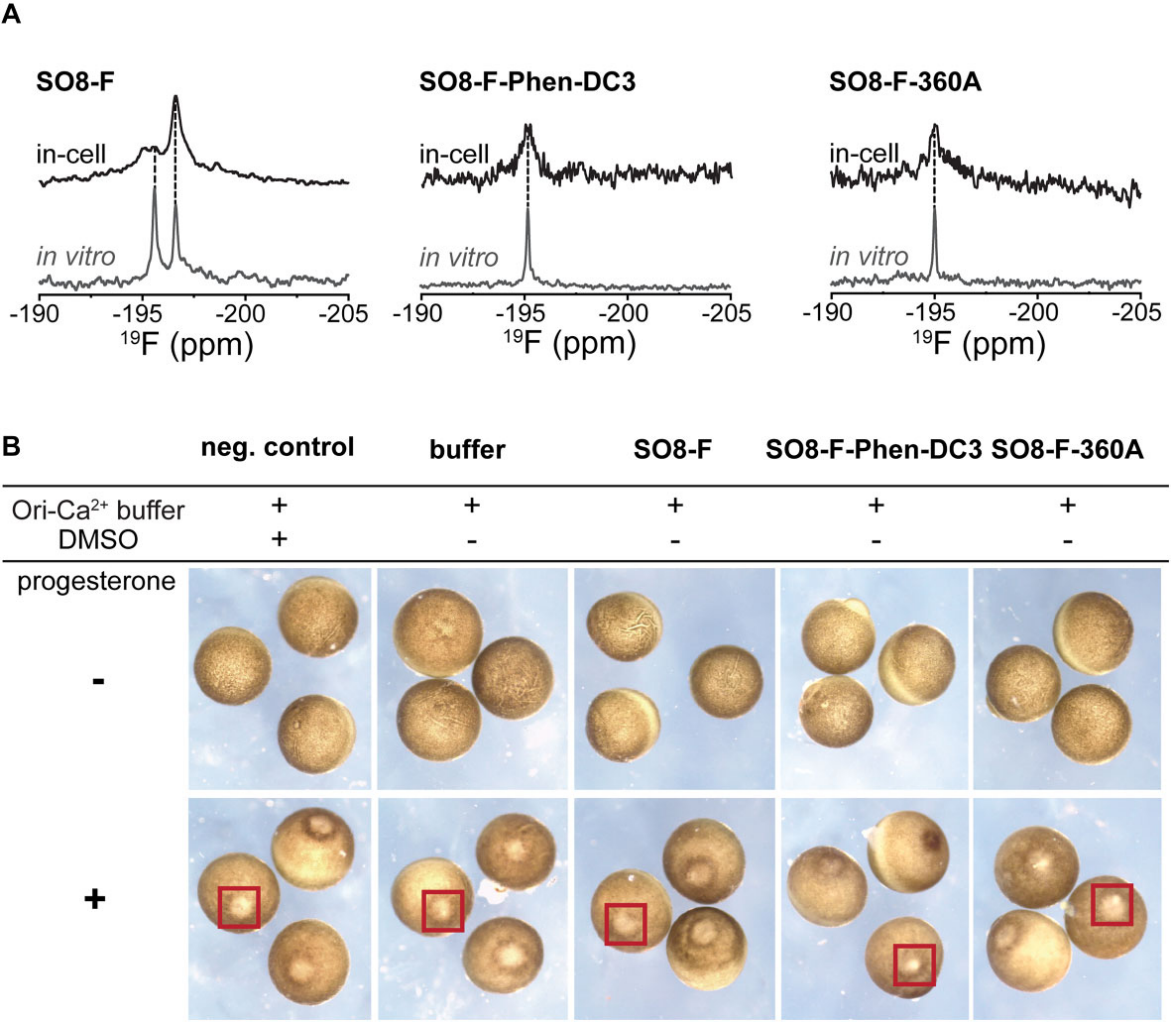
(**A**) Comparison of *in vitro* (gray) and in-cell (black) ^19^F NMR spectra of SO8-F and 1:1 SO8-F-Phen-DC3 and SO8-F-360A complexes, respectively. (**B**) Images of the *X. laevis* oocytes before and after progesterone treatment. Progesterone was dissolved in DMSO and added to the samples at a concentration of 1 μM. Negative control represents oocytes without DNA/DNA–ligand microinjection but with the addition of pure DMSO. The buffer sample contains Ori-Ca^2+^ buffer with the addition of DNA dissolving buffer. In the case of other samples, prefolded DNA and 1:1 DNA–ligand constructs were microinjected into ∼300 *X. laevis* oocytes. The evidence of maturation (white spot) is marked with red rectangles.

SO8-F, similar to SO8, exhibited polymorphism, forming both hybrid and antiparallel QDHs as evidenced by characteristic fingerprints in the imino region of the ^1^H NMR spectrum (Supplementary Fig. S30A). However, SO8-F showed a higher proportion of antiparallel QDH compared to SO8, with a hybrid-to-antiparallel ratio of ∼50:50 versus ∼80:20 for SO8. This was corroborated by the ^19^F NMR spectrum of SO8-F, which showed two signals of similar intensity at ∼−195.5 and −196.5 ppm (Fig. 6 and Supplementary Fig. S30A). By contrast, SO2-F, which adopts a single antiparallel conformation, displayed one sharp peak at ∼−197.3 ppm (Supplementary Fig. S30B).

^1^H and ^19^F NMR titrations show that Phen-DC3 and 360A shift the equilibrium of SO8-F from a hybrid/antiparallel QDH mixture toward a single, well-defined hybrid QDH–Phen-DC3/360A complexes at (sub-)stoichiometric ratios (0.1–1.0 ligand eq.) (Supplementary Figs S31–S32). At higher ligand excess (≥2 eq. for Phen-DC3 and ≥1.5 eq. for 360A), signal broadening and the appearance of multiple low-intensity peaks indicate non-specific binding and structural heterogeneity. This observation is corroborated by CD melting data obtained for the SO8-Phen-DC3 and SO8-360A constructs, which demonstrate that the presence of ligand in excess (≥2 eq.) induces non-specific binding and potentially leads to unfolding of the QDH structure (Supplementary Figs S10–S11). In contrast, the ^19^F NMR spectra of SO2-F showed minimal changes after ligand addition, with only a minor (∼20%) reduction of peak intensity, likely due to non-specific interactions (Supplementary Fig. S30B). These results confirmed the ligands’ strong preference for binding and stabilizing the hybrid QDH conformation over the antiparallel form.

The ^19^F NMR spectrum of free SO8-F microinjected into *X. laevis* oocytes revealed a dominant signal at ∼−196.5 ppm, corresponding to the antiparallel conformation, with a hybrid-to-antiparallel QDH ratio of ∼1:5 (Fig. 6A). A lower-intensity signal at −195.5 ppm was attributed to the hybrid form, while a minor signal at −195.0 ppm remained unassigned. Control experiments ruled out degradation or leakage of SO8-F and suggested that this additional signal likely corresponds to a low-population hybrid-like or protein-bound SO8-F species localized in cells (Supplementary Fig. S33). The conformational distribution of the hybrid and antiparallel QDH structures in the intraoocyte buffer (which mimics the electrolyte composition of *X. laevis* oocytes) remained unchanged relative to *in vitro* conditions (K^+^-based buffer) (Supplementary Fig. S34) [86]. These findings suggest that intracellular factors beyond ion composition and ionic strength, such as molecular crowding/confinement, viscoelasticity, osmolytes, metabolytes, and protein interactions, govern the conformational distribution *in vivo*. Investigating the influence of these intracellular factors on the conformational equilibria lies beyond the scope of this study.

Interestingly, the ^19^F NMR spectra of pre-formed 1:1 SO8-F-Phen-DC3 and SO8-F-360A complexes microinjected into oocytes closely resembled their *in vitro* counterparts, showing a single signal associated with the hybrid conformation (Fig. 6A and Supplementary Fig. S33). This demonstrates that both ligands maintain their binding specificity and stabilize the hybrid conformation even in a crowded intracellular environment. Furthermore, Phen-DC3 and 360A shifted the equilibrium toward the hybrid form despite the cellular preference for the antiparallel *PIM1* QDH and the higher tendency of SO8-F to form an antiparallel structure compared to SO8. Control of degradation and leakage control experiments confirmed that the observed in-cell ^19^F NMR signals were due to intact SO8-F complexes within the cells and not from degradation products or leakage into the extracellular medium (Supplementary Fig. S33). Importantly, oocyte integrity was verified by progesterone-induced maturation assays (Fig. 6B). These results demonstrate that the oocytes remained viable throughout the entire procedure, including the injection process and the NMR measurement.

The in-cell NMR data demonstrated that the introduced complexes retain their native folding inside cells, highlighting the ability of Phen-DC3 and 360A to selectively bind and stabilize the hybrid *PIM1* QDH conformation in the cellular context.

## Discussion

Phen-DC3 is a well-established ligand for stabilizing G-quadruplex (G4) structures, with recent studies unveiling its preferential binding to the Q-D interface rather than to the exposed G-quartets [25, 26]. This specificity suggests that Phen-DC3 and other bis-quinolinium derivatives (i.e. 360A) could serve as promising scaffolds for selectively targeting QDHs (Supplementary Fig. S35). Previous research demonstrated Phen-DC3’s ability to intercalate at the Q-D junction in hybrid and parallel QDHs derived from non-native telomeric (TelQD) and c-Myc G4-forming sequences, respectively [25, 26]. Moreover, Phen-DC3 induced refolding of antiparallel TelQD into hybrid QDH conformation [26]. However, limited high-resolution structural data have left the molecular determinants of (bis-quinolinium-based) ligand specificity and the factors governing their interaction with various QDH sequences and topologies largely unexplored.

In this study, we investigated interactions between a polymorphic QDH-forming sequence and its two monomorphic variants derived from the *PIM1* gene promoter (*PIM1* QDH) and two bis-quinolinium ligands, Phen-DC3 and 360A. Under both *in vitro* and cell-like conditions, the ligands demonstrated high specificity for the hybrid conformation of the polymorphic *PIM1* QDH, with negligible binding observed to the co-existing antiparallel form or evidence of ligand-induced refolding to the hybrid form. High-resolution structural analysis revealed that Phen-DC3 intercalates into the interface formed by the exposed 3′ G-quartet and a coaxially stacked Watson-Crick G-C base pair, as recently observed for c-Myc and TelQD QDHs [25, 26]. Although 360A binds the same pocket, the structural comparison revealed notable differences in ligand behavior.

Phen-DC3, with its larger phenanthroline ring, achieves optimal stacking between the G9–G20 edge of G5•G9•G20•G27 quartet and G19–C10 base pair, adopting a single, well-defined orientation within the binding pocket. In contrast, 360A, featuring a smaller pyridine group, exhibits suboptimal stacking, leading to dynamic reorientation within the pocket and reduced thermodynamic stability of the hybrid *PIM1* QDH-360A complex. These findings emphasize the importance of stacking interactions and ligand rigidity for high-affinity binding, offering valuable insights for designing hybridized ligands that combine G4-specific and duplex-binding moieties [17–22, 87–90].

Unlike previous reports of ligand-induced refolding of antiparallel QDHs into hybrid forms, almost no binding or refolding of the antiparallel *PIM1* QDH was observed with either ligand at 1:1 DNA:ligand ratios, even at prolonged incubation periods [26]. This difference likely arises from the variations in Q-D interface structures. In TelQD, a T-A:G-C pseudo-quartet caps the 3′ G-quartet, obstructing ligand access and requiring cap disruption for Phen-DC3 binding. While this disruption incurs an energetic penalty, it also facilitates refolding to a hybrid topology [26]. In contrast, our results suggest that the 5′-GC overhang in SO8 facilitates the ligand-induced conformational transition from an antiparallel to a hybrid topology. This effect is likely mediated by the formation of a stabilizing GCC triad, as supported by restrained molecular dynamics simulations (Supplementary Fig. S22). The absence of this overhang in SO2 appears to impose a higher energetic penalty for the ligand-induced transition [36].

Our results highlight the critical role of the Q-D junction as an optimal binding site for Phen-DC3 and 360A. The exposed 3′ G-quartet, in combination with a coaxially stacked stem-loop domain, facilitates ligand binding in hybrid QDHs with single-nucleotide propeller loops. In the antiparallel QDH construct, lacking the 5′-GC overhang, no comparable ligand-induced refolding is observed, underscoring the unique contribution of this capping element in modulating structural plasticity and ligand responsiveness (Table 1).

Although high-resolution structural data were obtained under physiologically relevant potassium-rich conditions using complementary experimental and computational methods, they do not fully account for the complex intracellular environment that may influence DNA folding and ligand binding [81–83, 86]. Nevertheless, in-cell NMR experiments conducted in *X. leavis* oocytes confirmed that both ligands’ selectivity and binding modes with hybrid *PIM1* QDH were preserved under physiological conditions, supporting the *in vitro* findings. Notably, the preferential formation of the antiparallel *PIM1* QDH conformation in cells without ligand compared to *in vitro* conditions suggests that the intracellular environment actively modulates *PIM1* QDH structural polymorphism. Although this phenomenon lies beyond the primary scope of this study, it warrants further investigation.

## Conclusion

This work provides atomically resolved insights into the binding epitopes of two bis-quinolinium-based ligands, Phen-DC3 and 360A, within the hybrid QDH structure formed by a native sequence from the promoter region of the *PIM1* (onco)gene. Our data revealed the critical role of 5′-GC overhang and the capping structures at the 3′ G-tetrad of the Q-D interface in modulating the QDH refolding upon ligand binding and the importance of the ligand's aromatic scaffold for effective interaction. These results are vital for developing ligands that selectively target various QDH structures. This study contributes to a deeper understanding of the dynamic structural equilibrium of non-canonical G-rich sequences under physiological conditions in the presence of potent small molecules. Furthermore, the ability to modulate refolding between diverse QDH topologies through rational design of capping structures and ligands opens new possibilities for creating DNA nanodevices and molecular switches. From a methodological perspective, our in-cell ^19^F NMR data demonstrated the effectiveness of 2′-FANA-modified DNA targets as valuable tools for drug screening *in vitro* and in-cell-like environments.

## Supplementary Material

gkaf894_Supplemental_Files

## Data Availability

The NMR-derived atomic coordinates and chemical shifts for 1:1 SO7–Phen-DC3 (PDB ID: 9GVI, BMRB ID: 34 960) and SO7–360A (PDB ID: 9GVV, BMRB ID: 34 961) complexes were deposited in the PDB and the BMRB. The starting and output structures from the MD simulation for SO7–Phen-DC3 and SO7–360A complex, as PDB coordinates, are attached in the Supplementary Data. Due to their large size, MD trajectories can be obtained from the corresponding authors upon reasonable request.
