# Supplementary material for: Structural basis of bis-quinolinium ligands binding to quadruplex–duplex hybrids from PIM1 oncogene
Source: Nucleic Acids Res. 2025 Sep 12;53(17):gkaf894. doi: 10.1093/nar/gkaf894 (PMC12445686; doi:10.1093/nar/gkaf894)
Supplement: gkaf894_Supplemental_Files [file gkaf894_supplemental_files.zip › NAR-SO7-MS_SI_Rev_master_020825.pdf]

Supporting Information

Structural basis of Bis-quinolinium ligands binding to Quadruplex-duplex hybrids from *PIM1* oncogene

Anirban Ghosh[1,*], Jakub Harnos[2], Petr Stadlbauer[3, 4], Jiri Sponer[3, 5], Martina Lenarcic Zivkovic[6,*], and Lukas Trantirek[1,*]

[1] Central European Institute of Technology (CEITEC), Masaryk University, Brno 62500, Czech Republic

[2] Department of Experimental Biology, Faculty of Science, Masaryk University, Brno 62500, Czech Republic

[3] Institute of Biophysics, Czech Academy of Sciences, Brno 61200, Czech Republic

[4] Department of Physical Chemistry, Faculty of Science, Palacký University Olomouc, Olomouc 779 00, Czech Republic

[5] Regional Center of Advanced Technologies and Materials, The Czech Advanced Technology and Research Institute (CATRIN), Palacký University Olomouc, Olomouc 78 371, Czech Republic

[6] Slovenian NMR Centre, National Institute of Chemistry, Ljubljana 1000, Slovenia

* To whom correspondence should be addressed. Tel: +420 549496476; Email: lukas.trantirek@ceitec.muni.cz

Correspondence may also be addressed to martina.lenarcic@ki.si and anirban.ghosh@ceitec.muni.cz

The complete assignment of SO7-Phen-DC3 and SO7-360A 1:1 complexes by 2D NMR.

The ^{15}N- and ^{13}C-filtered HSQC NMR spectra enabled the assignment of all imino (H1) and aromatic protons (H8) of guanines involved in the formation of three G-quartet planes and three stem-loop base pairs (Supplementary Figure S12). The three G-quartet planes were assigned based on the intra-quartet guanine (H1)-guanine (H8) cross-peaks in 2D ^{1}H-^{1}H NOESY (τ_m = 300 ms) for both complexes (Figure 2). In contrast, three pairs of sequential guanine imino (H1)-cytosine amino (H41/H42 for hydrogen-bonded/non-hydrogen-bonded) cross-peaks revealed Watson-Crick hydrogen bonding signatures in the duplex stem-loop. The 2D NOESY NMR spectra (τ_m = 100 ms) of SO7-Phen-DC3 and SO7-360A 1:1 complexes exhibited strong intensities of intra-residue H8–H1' NOE cross-peaks for G1, G3, G7, G20, G21, and G25, indicating that these residues occupy *a syn* glycosidic conformation (Supplementary Figure S13). This is supported by the downfield ^{13}C-C8 chemical shift of these residues (Supplementary Figure S14) (1–3). All other residues (guanines, adenines, cytosines, and thymine) adopted an *anti*-glycosidic conformation, as evident from the weak H8–H1' NOE cross-peaks and upfield ^{13}C-C8 chemical shifts (Supplementary Figures S13-S14). The anomeric-aromatic region of the 2D NOESY spectra (τ_m = 300 ms) exhibited an NOE cross-peak pattern between H8(n)−H1'(n+1) and H1'(n)-H8(n+1) for G3/G4, G7/G8, G21/G22, and G25/G26, indicating *syn-anti* guanine stacking in the complexes (4). G4/G5, G8/G9, and G26/G27 pairs showed *anti-anti* guanine stacking, while a *syn-syn* stacking step was confirmed for the G20/G21 pair based on H1'(n)-H8(n+1) and H8(n)-H1' (n+1) cross peaks in NOESY spectra. The stereospecific assignment of the H2' and H2'' protons was performed by qualitatively evaluating the intensities of the H1′–H2′ and H1′–H2″ cross-peaks in the NOESY spectrum, where stronger intensities for H1'–H2'' signals were expected. These were then compared to H8-H2' and H8-H2" intensities, where, due to the closer proximity of H8 to H2' atoms, higher intensities of H8-H2' were observed. Based on the observed higher intensities of H1'-H2' cross-peaks (due to larger scalar coupling) than the H1'-H2" cross-peaks in DQF-COSY, all nucleotides in the complex embrace the south-type sugar pucker conformations (Supplementary Figure S15). Both ligands contain symmetry in their conformation, making a chemically equivalent set of protons in the free state, while DNA binding breaks the degeneracy of their chemical shifts. Consequently, upon binding to SO7, two sets of proton resonances were

observed for the chemically equivalent protons of both ligands. The imino, aromatic, and methyl protons of the Phen-DC3 and 360A were assigned using a combination of ^{1}H-^{13}C HSQC, 2D TOCSY, NOESY, and ROESY NMR spectra, as described previously (Supplementary Figures S17-S18, Supplementary Table S5) (5–8).

Table S1. Changes in thermal stabilities of *PIM1*-derived sequences in the presence of Phen-DC3 and 360A at a 1:2 DNA:ligand molar ratio, as evaluated from CD melting experiments. The reported values are the average ± s.d of three repeats.

Complex	T_m (°C)	ΔT_m (°C)[a]
**SO7**	61.6 ± 1.3	
**SO7-Phen-DC3**	79.5 ± 1.1	17.9 ± 1.7
**SO7-360A**	73.4 ± 1.0	11.8 ± 1.6
**SO2**	59.6 ± 0.8	
**SO2-Phen-DC3**	66.1 ± 0.8	6.5± 1.1
**SO2-360A**	63.5 ± 0.3	3.9 ± 0.9
**SO8[b]**	58.0 ± 1.0	
**SO8-Phen-DC3**	74.4 ± 1.1	16.4 ± 1.5
**SO8-360A**	73.1 ± 0.6	15.1 ± 1.2

[a]ΔT_m represents the difference in thermal melting temperature, $\Delta T_m = |T_{m(DNA\text{-}ligand)} - T_{m(DNA)}|$.
[b]T_m represents the global T_m owing to the polymorphic nature of SO8.

Table S2. Changes in thermal stabilities of *PIM1*-derived sequences in the presence of Phen-DC3 and 360A at a 1:3 DNA:ligand molar ratio, as evaluated from CD melting experiments. The reported values are the average ± s.d of three repeats.

	T_m (°C)	ΔT_m (°C)[a]
**SO7**	61.6 ± 1.3	
**SO7-Phen-DC3**	72.2 ± 0.5	10.6 ± 1.4
**SO7-360A**	68.1 ± 1.0	6.5 ± 1.6
**SO2**	59.6 ± 0.8	
**SO2-Phen-DC3**	68.5 ± 0.4	8.9 ± 0.9
**SO2-360A**	66.8 ± 0.5	7.2 ± 0.9
**SO8**[b]	58.0 ± 1.0	
**SO8-Phen-DC3**	69.6 ± 0.5	11.6 ± 1.1
**SO8-360A**	66.2 ± 0.4	8.2 ± 1.1

[a]ΔT_m represents the difference in thermal melting temperature, $\Delta T_m = |T_{m(DNA\text{-}ligand)} - T_{m(DNA)}|$.
[b]T_m represents the global T_m owing to the polymorphic nature of SO8.

Table S3. ^{1}H and ^{13}C chemical shifts (ppm) of SO7 in complex with Phen-DC3 at 1: 1 ratio.[a]

Residue/δ	H8/H6	H1	H2/H5/Me	H1'	H2'	H2''	H3'	H4'	C8/C6	C2/C5
G1	7.25			5.96	2.15	2.88	4.32	3.93	138.98	
C2	7.36		5.67	5.86	1.60	2.24	4.64	3.95	139.90	95.91
G3	7.50	11.74		6.08	3.57	3.00	5.11	4.38	139.43	
G4	7.89	11.08		5.95	2.56	2.90	5.10	4.41	135.80	
G5	7.70	10.39		6.27	2.20	2.40	4.79	4.35	134.67	
A6	8.59		8.34	6.69	2.89	3.07	5.20	4.37	139.72	152.79
G7	7.18	11.16		5.93	2.88	3.01	5.11	4.29	138.45	
G8	7.81	11.49		5.69	2.48	2.66	4.98	4.35	136.14	
G9	7.93	10.23		6.00	3.05	2.89	5.24	4.49	135.19	
C10	7.57		5.51	5.38	2.31	2.41	4.86	4.43	140.60	96.92
G11	7.78	12.44		5.87	2.50	2.64	5.00	4.40	135.17	
C12	7.00		5.10	5.89	1.65	2.24	4.78	4.10	139.23	95.13
G13	7.84			6.04	2.21	2.54	4.91	4.53	135.06	
C14	7.52		5.74	6.05	1.96	2.36	4.52	3.75	140.82	95.10
C15	7.52		5.68	5.73	1.73	1.82	4.38	3.78	140.96	95.67
A16	8.11		7.85	5.68	2.74	2.44	4.93	4.10	136.52	152.41
G17	7.76	12.81		5.07	2.35	2.13	4.70	3.82	135.31	
C18	6.77		4.86	5.60	1.23	1.97	4.48	3.96	139.46	95.56
G19	7.46	11.52		5.72	2.82	3.01	5.18	4.26	135.20	
G20	6.72	11.09		5.99	3.07	2.90	4.32	3.51	139.44	
G21	7.13	10.88		5.70	2.57	2.53	4.99	4.25	137.86	
G22	7.71	11.68		5.94	2.66	2.44	5.09	4.30	135.00	
T23	7.71		1.90	6.33	2.89	2.92	5.28	4.73	137.16	
C24	7.35		5.33	6.01	2.22	2.53	4.86	4.12	139.33	95.25
G25	7.26	11.49		6.10	3.53	2.99	5.06	4.50	139.25	
G26	7.85	11.34		5.97	2.50	2.86	5.06	4.38	134.91	
G27	7.54	10.93		6.22	2.76	2.81	4.86	4.38	135.73	

[a]At 298.2 K in 20 mM potassium phosphate buffer, 20 mM KCl, pH 7.1.

Table S4. ^{1}H and ^{13}C chemical shifts (ppm) of SO7 in a complex with 360A at 1: 1 ratio.[a]

Residue/ δ	H8/H6	H1	H2/H5/Me	H1'	H2'	H2''	H3'	H4'	C8/C6	C2/C5
G1	7.26			5.99	2.18	3.37	4.92	4.39	139.19	
C2	7.39		5.70	5.88	1.61	2.25	4.64	3.95	139.83	95.92
G3	7.51	11.80		6.08	3.62	3.00	4.90	4.28	139.43	
G4	7.89	11.13		5.91	2.50	2.91	5.09	4.12	136.41	
G5	7.38	10.84		6.07	2.24	2.57	4.91	4.36	134.94	
A6	8.58		8.33	6.69	3.05	2.89	5.20	4.38	139.74	152.80
G7	7.19	11.22		5.94	2.89	3.01	5.07	4.27	138.45	
G8	7.90	11.56		5.70	2.58	2.75	5.08	4.27	135.79	
G9	7.75	10.32		6.07	2.77	2.25	5.15	4.31	135.12	
C10	7.57		5.52	5.57	2.31	2.46	4.86	4.37	140.66	97.02
G11	7.82	12.58		5.89	2.53	2.66	4.99	4.39	135.35	
C12	7.03		5.13	5.93	1.67	2.26	4.97	4.12	139.25	95.14
G13	7.87			6.04	2.29	2.56	4.77	4.00	138.01	
C14	7.54		5.76	6.07	1.99	2.38	4.53	4.03	140.97	95.64
C15	7.53		5.68	5.74	1.75	1.83	4.40	3.80	140.74	95.91
A16	8.12		7.89	5.71	2.76	2.45	4.94	4.47	136.55	152.45
G17	7.81	12.88		5.15	2.39	2.18	4.74	4.51	135.67	
C18	6.87		4.95	5.63	1.33	1.97	4.59	4.01	139.70	95.73
G19	7.17	11.94		5.86	2.64	2.87	5.05	4.27	135.76	
G20	7.15	10.81		6.05	3.05	2.57	4.54	4.35	139.42	
G21	7.27	10.94		5.76	2.61	2.55	5.00	4.30	138.07	
G22	7.74	11.74		5.96	2.50	2.81	5.06	4.47	134.74	
T23	7.72		1.91	6.28	2.21	2.42	4.83	4.37	137.17	
C24	7.37		5.36	5.98	2.46	2.70	4.93	4.23	139.34	95.25
G25	7.26	11.54		6.10	3.54	3.00	4.93	4.31	139.21	
G26	7.84	11.48		5.98	2.70	2.48	4.35	3.94	135.05	
G27	7.73	10.89		6.44	2.78	2.90	4.89	4.35	135.86	

[a]At 298.2 K in 20 mM potassium phosphate buffer, 20 mM KCl, pH 7.1.

Table S5. ^{1}H chemical shifts (ppm) of Phen-DC3 and 360A in SO7-Phen-DC3 and SO7-360A 1: 1 complexes, respectively.[a]

Atom names	Phen-DC3	360A
	^{1}H (ppm)	
A	12.21	11.10
A'	11.80	11.34
B	9.07	8.86
B'	8.93	8.83
C	10.32	9.98
C'	10.31	9.22
D	7.87	7.45
D'	7.84	7.61
E	7.77	7.96
E'	7.84	7.24
F	7.89	7.45
F'	7.96	7.86
G	8.07	8.03
G'	7.96	8.08
I	4.72	4.42
I'	4.62	4.51
H	7.65	8.15
H'	7.63	-
J	8.31	7.67
J'	8.35	-
K	7.91	8.05
K'	7.92	-

[a]At 298.2 K in 20 mM potassium phosphate buffer, 20 mM KCl, pH 7.1.

Table S6. Intermolecular NOE-derived distance restraints between SO7 and Phen-DC3 in the 1:1 SO7-Phen-DC3 complex. NOE cross-peak intensities were classified as strong (s), medium (m), or weak (w) during structure calculations.

Phen-DC3		A	A'	B	B'	C	C'	D	D'	E	E'	F	F'	G	G'	I	I'	H	H'	J	J'	K	H'
**G5**	H1			w				w								w							
	H8											w		w									
	H1'											w		w									
**G9**	H8					w												w		w			
	H1'																	w		m		w	
	H2'					w												w				w	
	H2''					w												m		m		w	
	H3'																	w		w			
**C10**	H6																			w			
	H5																	w					
	H1'																			w		w	
	H3'																	w					
	H4'																			w		w	
	H5'																			w			
**G19**	H8						w										w						
	H1																		w		w		w
	H1'																		w		w		
	H2'						m												m		w		
	H2''						w												m		w		
	H3'						w												w				
**G20**	H8																		w		m		w
	H1			w	w		w		w								w						
	H1'																		w		w		
**G27**	H8						w					w		w			w						
	H1			w	w			w	m	w													
	H1'											w		w									
	H2'											w		w									
	H2''											m		m									
	H3'											w		w									

Table S7. Intermolecular NOE-derived distance restraints between SO7 and 360A in the 1:1 SO7-360A complex. NOE cross-peak intensities are classified as strong (s), medium (m), and weak (w) during structure calculations.

360A		A	A'	B	B'	C	C'	D	D'	E	E'	F	F'	G	G'	I	I'	H	J	K
**G5**	H1	w		w																
	H8												w				w			
	H1'										w		w		w					
	H2'												w		w					
	H2''												w		w					
**G9**	H1															w				
	H1'											w								
	H2'							w		m		m		w						
**C10**	H6											m								
	H5									m										
	H1'											w								
**G19**	H8			w														w	w	w
	H1													w		w				
	H2'																	w	w	
	H2''																	w	w	
	H3'																	w	w	
**G20**	H8																w			
	H1																			w
**G27**	H1		w		w												w			
	H1'																			w
	H2'																			w
	H2''																			w

Table S8. NMR restraints and structural statistics for the SO7-Phen-DC3 1:1 complex.

**NMR restraints statistics**		
NOE-derived distance restraints	non-exchangeable	exchangeable
Intramolecular DNA NOEs		
Intra-residual	293	0
Sequential	104	5
Long-range	21	28
Intramolecular ligand NOEs	3	0
Intermolecular DNA-ligand NOEs	57	16
Torsion angle restraints	27	
Hydrogen-bond restraints	33	
Planarity restrains	42	
**Structural statistics**		
Distance (NOE) restraint violation >0.2Å	0	
Maximum distance restraint (NOE) violation (Å)	0.082	
Average distance restraint (NOE) violation (Å)	0.077 ± 0.016	
No. of torsion angle restraint violation > 2.0°	0	
Maximum torsion angle restraint violation (°)	0	
Average torsion angle restraint violation (°)	0	
**Pairwise heavy atom RMSD (Å)**		
Overall (DNA + Ligand)		1.51 ± 0.57
G-quartet core		0.60 ± 0.15
Q–D interface		0.26 ± 0.08
Duplex stem-loop		1.44 ± 1.08
Duplex stem-loop without G13-A16		0.37 ± 0.20
Phen-DC3		0.20 ± 0.18

Table S9. NMR restraints and structural statistics for the SO7-360A 1:1 complex.

**NMR restraints statistics**		
NOE-derived distance restraints	non-exchangeable	exchangeable
Intramolecular DNA NOEs		
Intra-residual	277	0
Sequential	104	5
Long-range	14	28
Intramolecular ligand NOEs	7	0
Intermolecular DNA-ligand NOEs	31	9
Torsion angle restraints	27	
Hydrogen-bond restraints	33	
Planarity restrains	42	
**Structural statistics**		
Distance restraint (NOE) violations >0.2Å	0	
Maximum distance restraint (NOE) violation (Å)	0.098	
Average distance restraint (NOE) violation (Å)	0.079±0.013	
No. of torsion angle restraint violation > 2.0°	1	
Maximum torsion angle restraint violation (°)	5.26	
Average torsion angle restraint violation (°)	5.26 ± 0.0	
**Pairwise heavy atom RMSD (Å)**		
Overall (DNA + Ligand)	1.82 ± 0.71	
G-quartet core	0.57 ± 0.18	
Q–D interface	0.55 ± 0.21	
Duplex stem-loop	2.21 ± 1.03	
Duplex stem-loop without G13-A16	0.49 ± 0.17	
360A	0.20 ± 0.11	

Figure S1. NMR titration of SO7 with Phen-DC3 up to 1:3 molar ratio. Imino signals of free SO7 are marked by red asterisks, while green dots denote the imino peaks of the SO7-Phen-DC3 complex formed at 1:1 molar ratio. Signals in the imino region belonging to Phen-DC3 are marked with L.

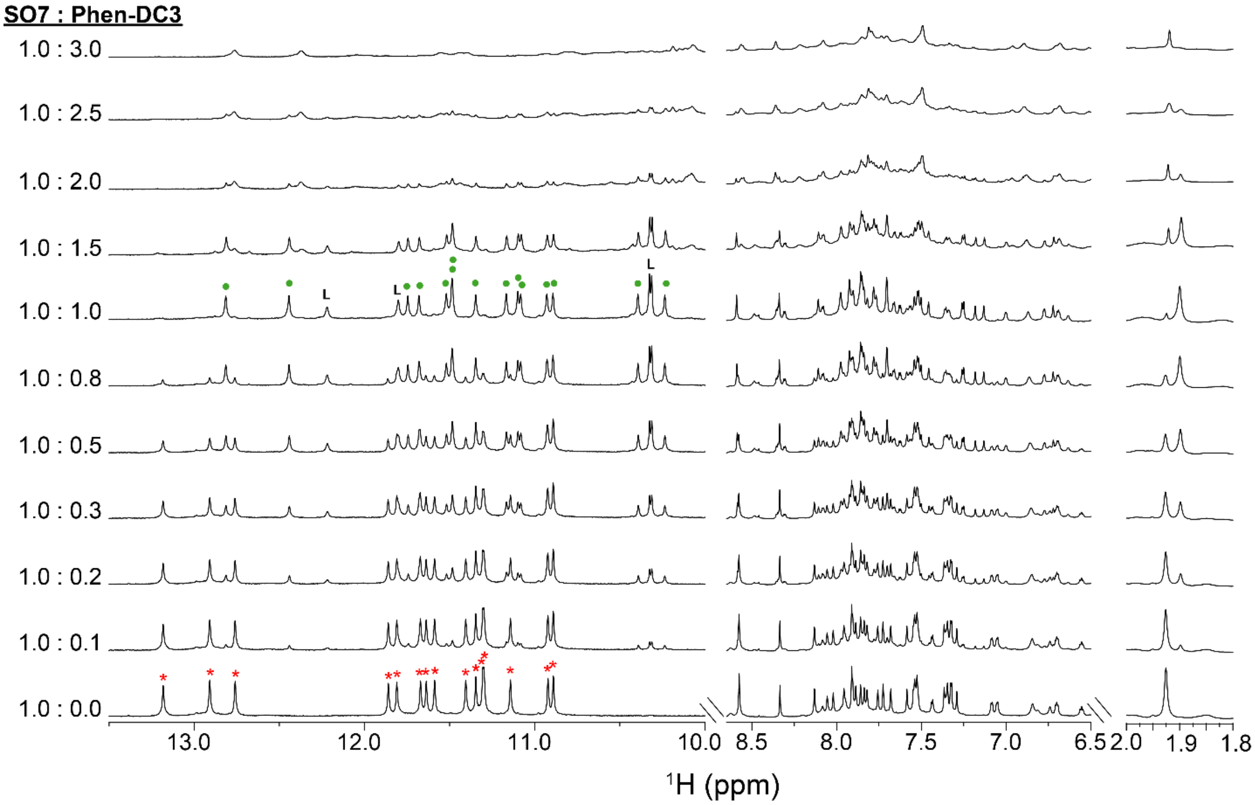

Figure S2. NMR titration of SO7 with 360A up to 1:3 molar ratio. Imino signals of free SO7 are marked by red asterisks, while blue dots denote the imino peaks of the SO7-360A complex formed at 1:1 molar ratio. Signals in the imino region belonging to 360A are marked with L.

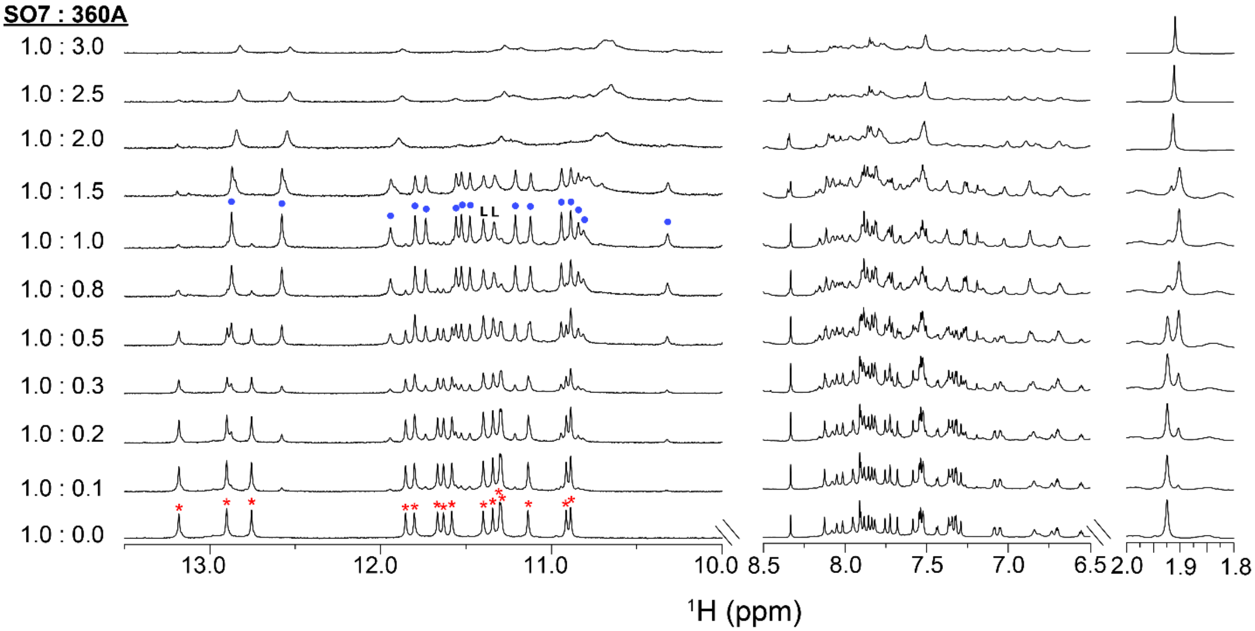

Figure S3. (A) Comparison of CD spectra and (B) normalized CD melting profiles of free SO7, SO2, and SO8, and in the equimolar presence of Phen-DC3 and 360A, respectively. CD spectra were performed in 20 mM potassium phosphate buffer, 20 mM KCl, pH 7.1, at 25 °C with 20 µM DNA and ligand concentrations.

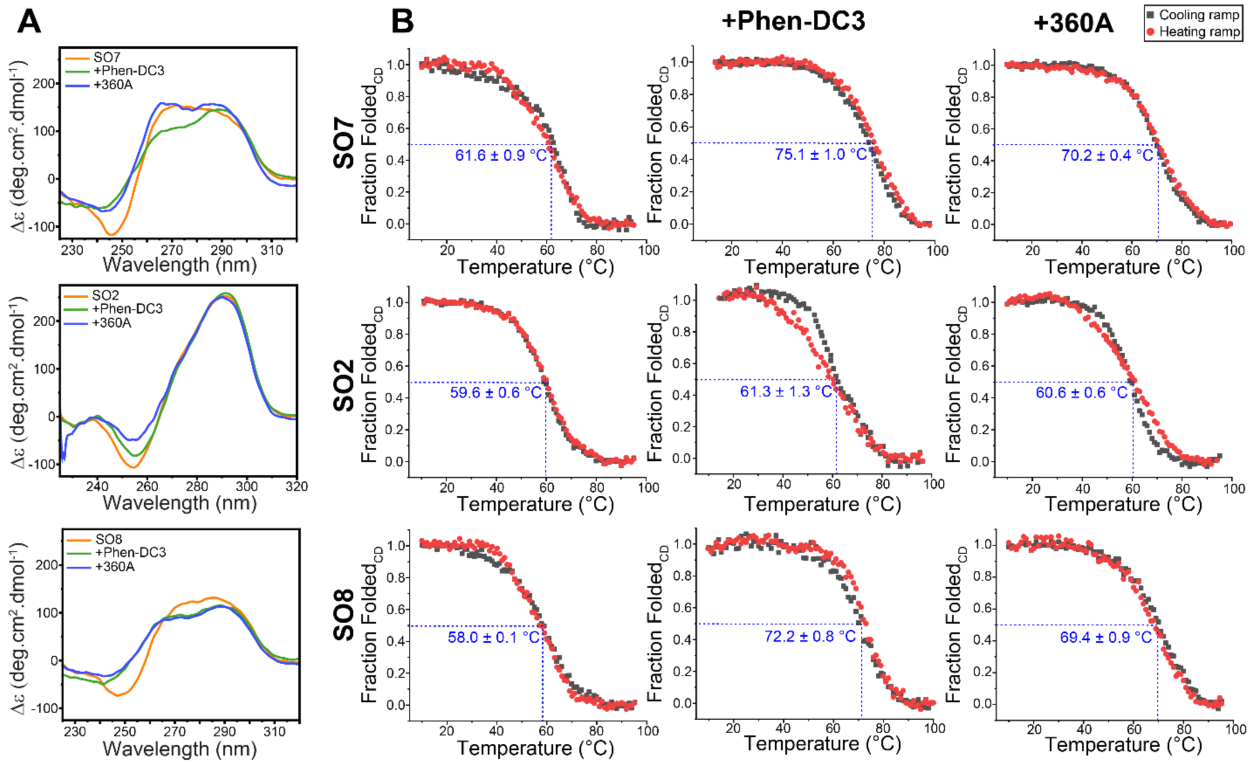

Figure S4. NMR titration of SO2 with (A) Phen-DC3 and (B) 360A up to a 1:3 molar ratio. Imino signals of free SO2 are marked by grey rectangles.

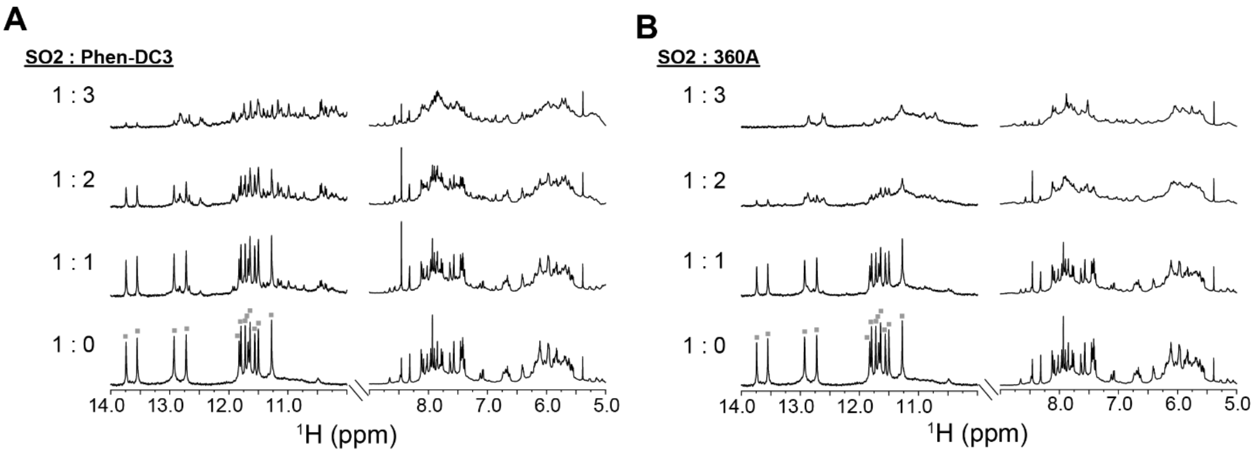

Figure S5. The imino region of the ^{1}H NMR spectra of free SO2 and SO2 incubated with Phen-DC3 (A) and 360A (B) at a 1:1 molar ratio for 1, 7, and 14 days. Grey rectangles and stars indicate non-overlapping imino signals (12–14 ppm) corresponding to free and ligand-bound species of SO2, respectively.

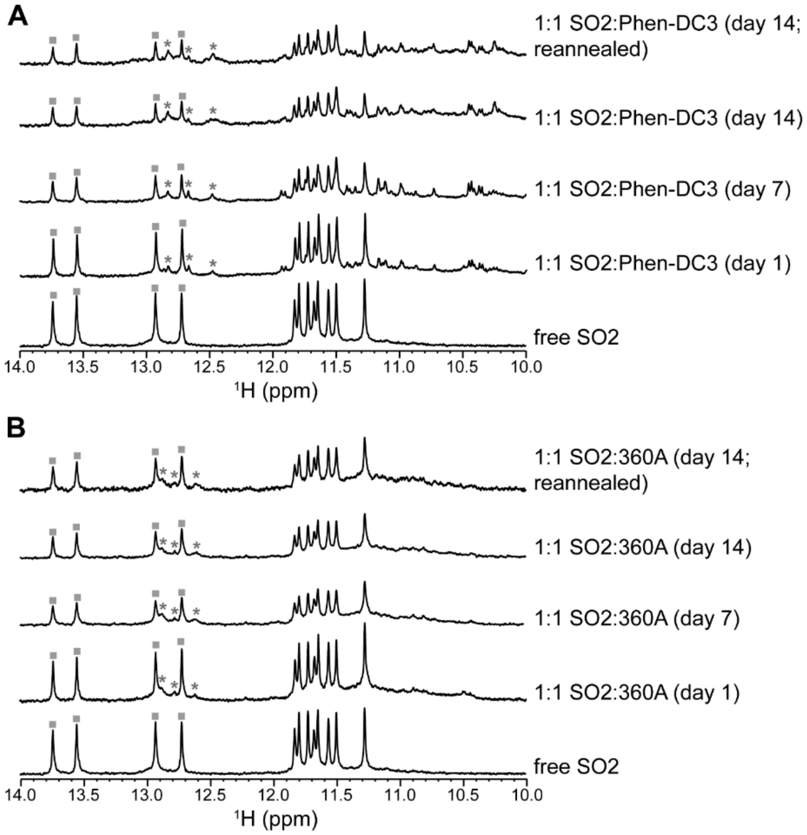

Figure S6. NMR titration of SO8 with Phen-DC3 up to 1:3 molar ratio. Red asterisks and grey rectangles indicate imino signals corresponding to hybrid (SO7-like) and antiparallel (SO2-like) QDH conformations of SO8, respectively. Imino signals belonging to a stable 1:1 SO8-Phen-DC3 complex are marked with green dots, while signals in the imino region corresponding to Phen-DC3 are labeled with L.

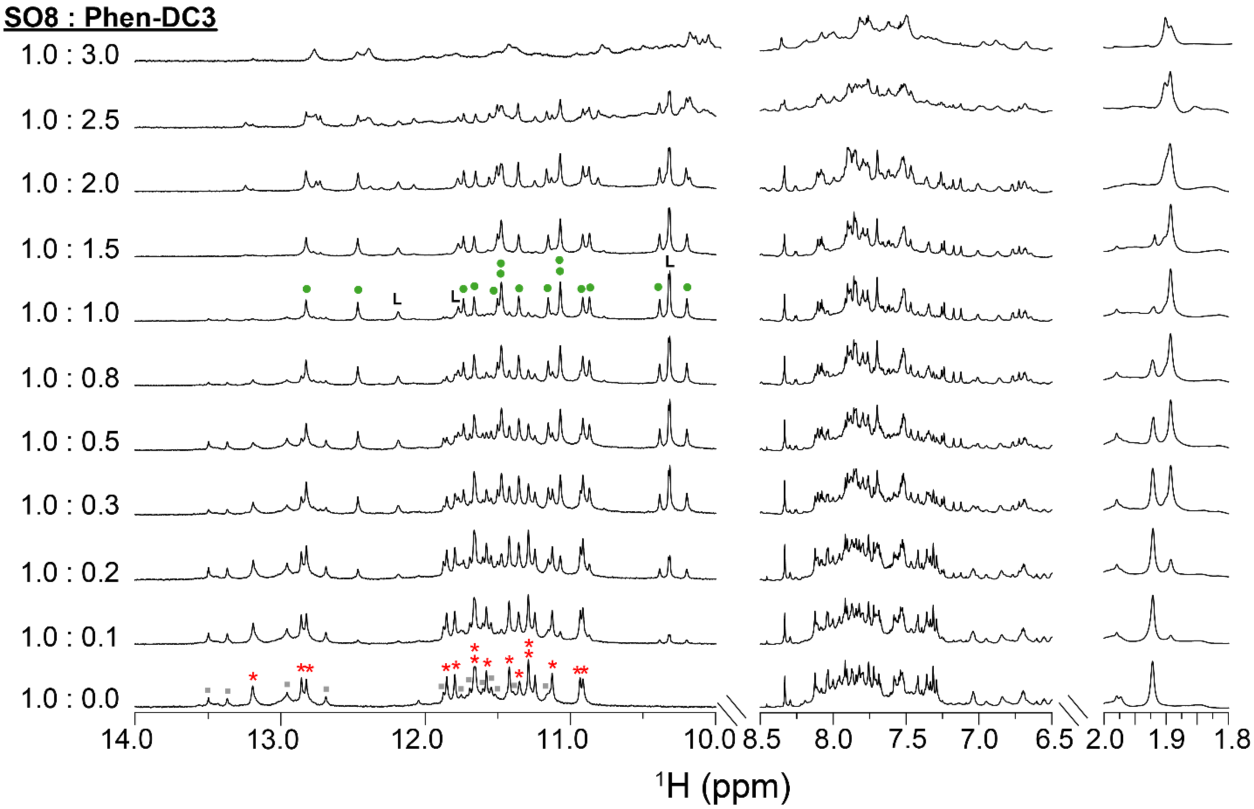

Figure S7. NMR titration of SO8 with 360A up to 1:3 molar ratio. Red asterisks and grey rectangles indicate imino signals corresponding to hybrid (SO7-like) and antiparallel (SO2-like) QDH conformations of SO8, respectively. Imino signals belonging to a 1:1 SO8-360A complex are marked with blue dots, while signals in the imino region corresponding to 360A are labeled with L.

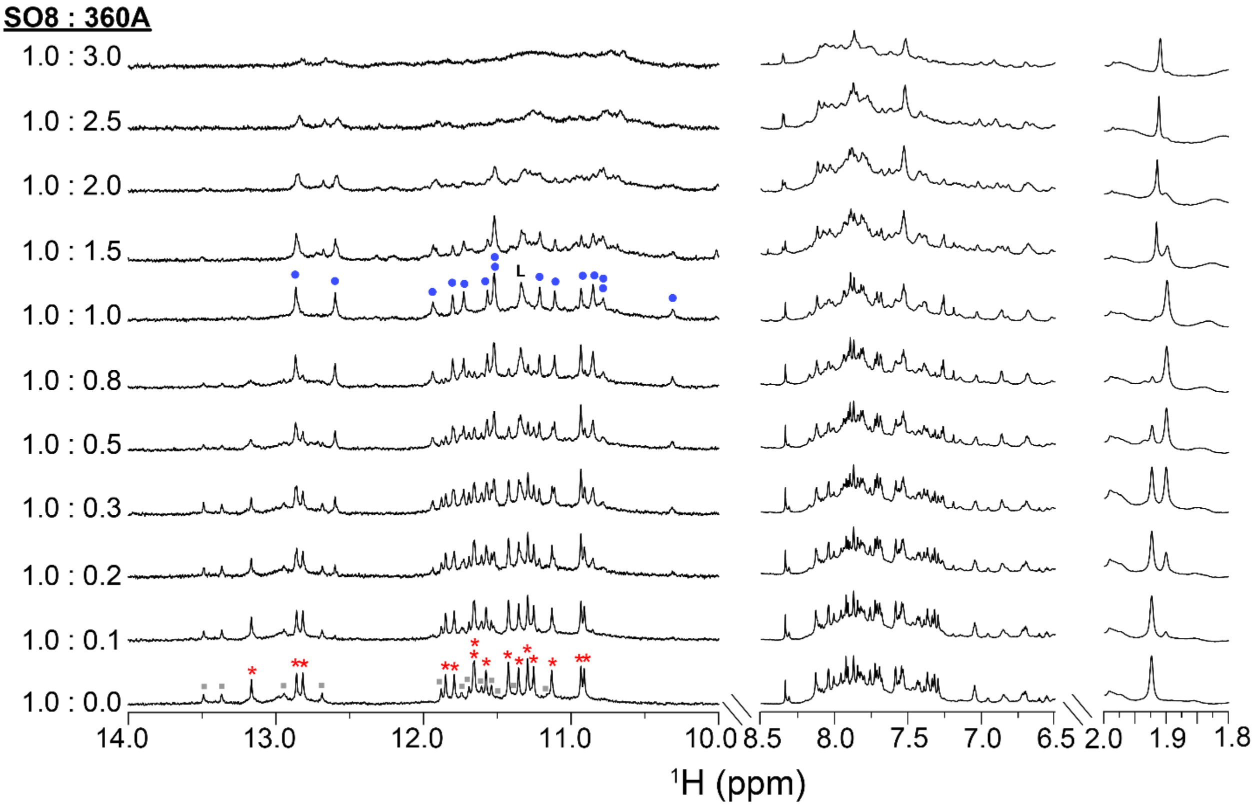

Figure S8. Superimposition of the regions of NOESY spectra (τ_m= 300 ms) of SO7 (blue) and SO8 (red) with (A) Phen-DC3 and (B) 360A at a 1:1 molar ratio. NOESY spectra were recorded in 20 mM potassium phosphate buffer, 20 mM KCl, pH 7.1, at 298.2 K with 0.3 mM DNA concentration.

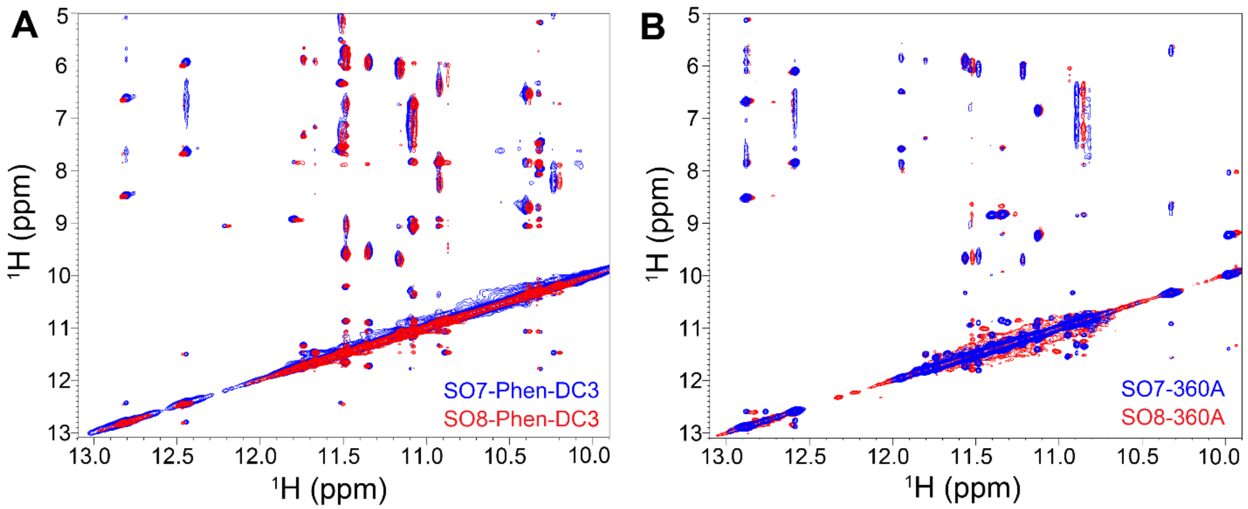

Figure S9. ^{1}H NMR competition experiment, performed on a 1:1 mixture of SO7 and SO2 with (A) Phen-DC3 and (B) 360A. In the topmost NMR spectra, only non-overlapping imino signals corresponding to different species were marked.

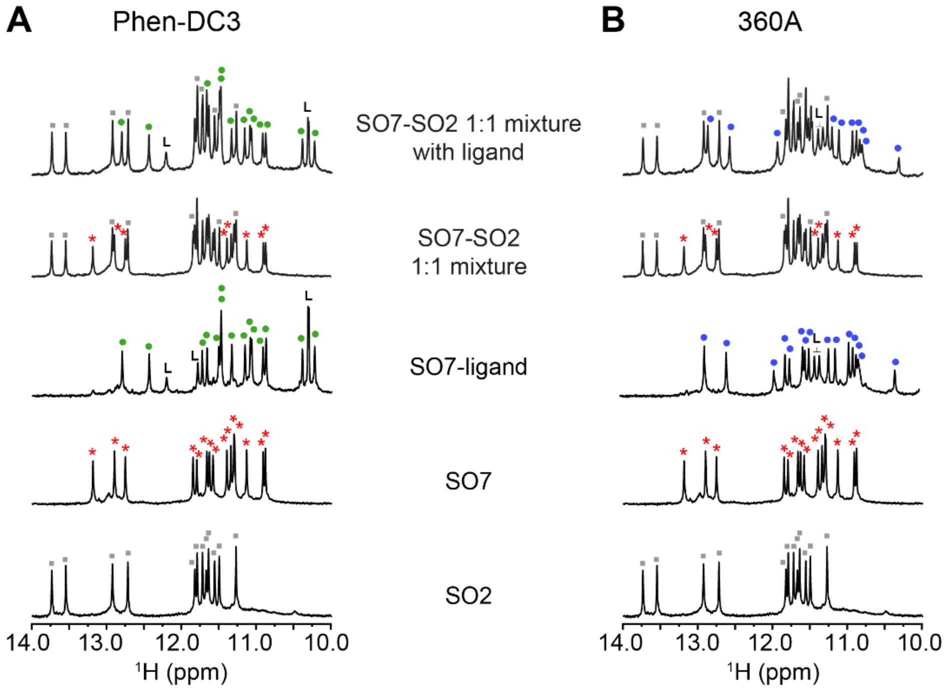

Figure S10. (A) Comparison of normalized CD melting profiles of SO7, SO2, and SO8 in the presence of two molar equivalents of (A) Phen-DC3 and (B) 360A, respectively. CD spectra were performed in 20 mM potassium phosphate buffer, 20 mM KCl, pH 7.1, at 25 °C with 20 μM DNA and 40 μM ligand concentrations.

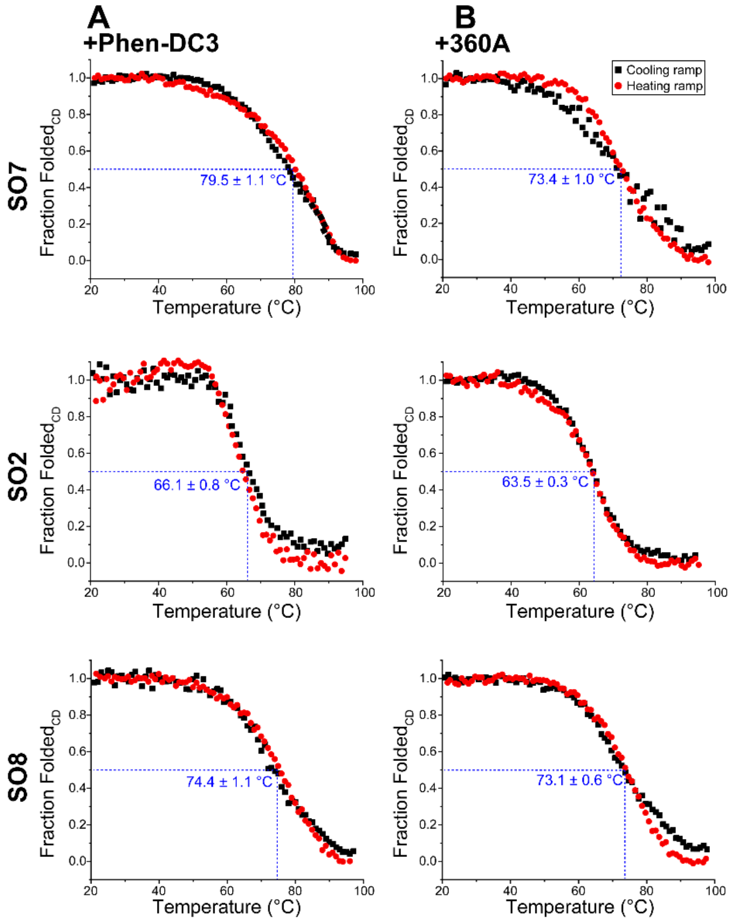

Figure S11. (A) Comparison of normalized CD melting profiles of SO7, SO2, and SO8 in the presence of three molar equivalents of (A) Phen-DC3 and (B) 360A, respectively. CD spectra were performed in 20 mM potassium phosphate buffer, 20 mM KCl, pH 7.1, at 25 °C with 20 µM DNA and 60 µM ligand concentrations.

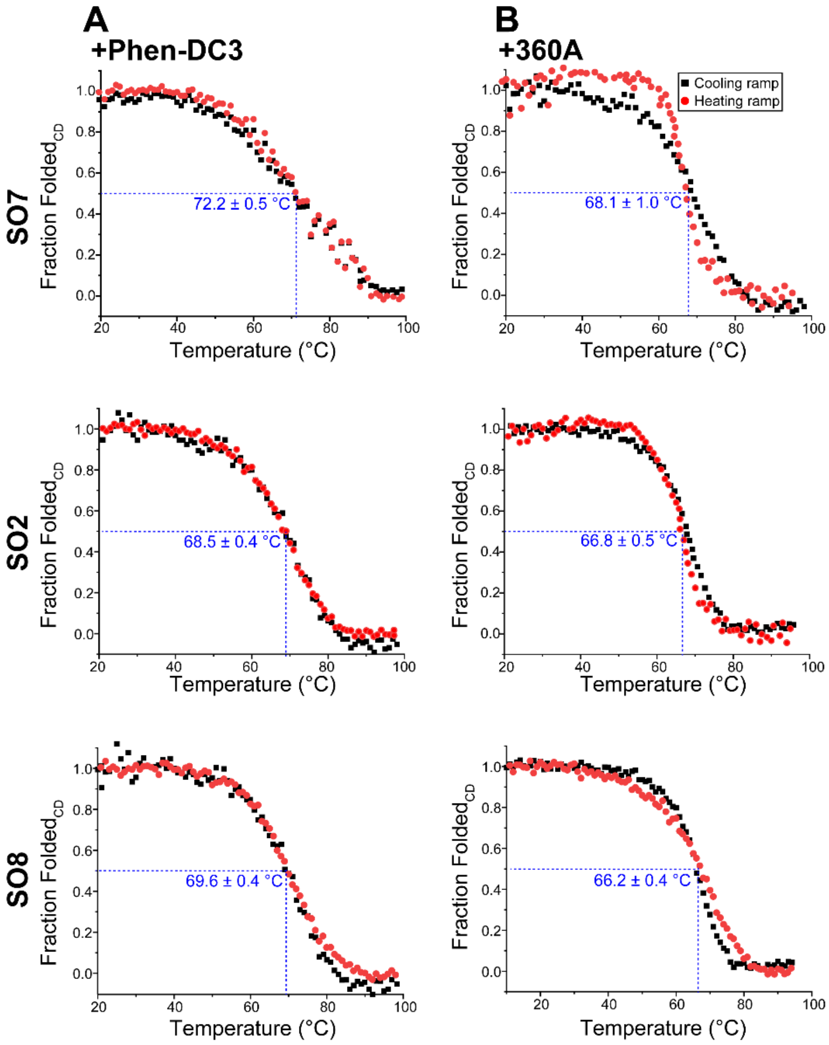

Figure S12. Unambiguous assignment of guanine imino (top) and aromatic (bottom) protons of (A) SO7-Phen-DC3 and (B) SO7-360A 1:1 complexes using ^{15}N- and ^{13}C-filtered HSQC NMR spectra acquired on site-specifically $^{13}C/^{15}N$-isotopically labeled SO7 oligonucleotides, respectively. All HSQC spectra were recorded in 20 mM potassium phosphate buffer with 20 mM KCl, pH 7.1, at 298.2 K with 0.3 mM DNA and ligand concentrations.

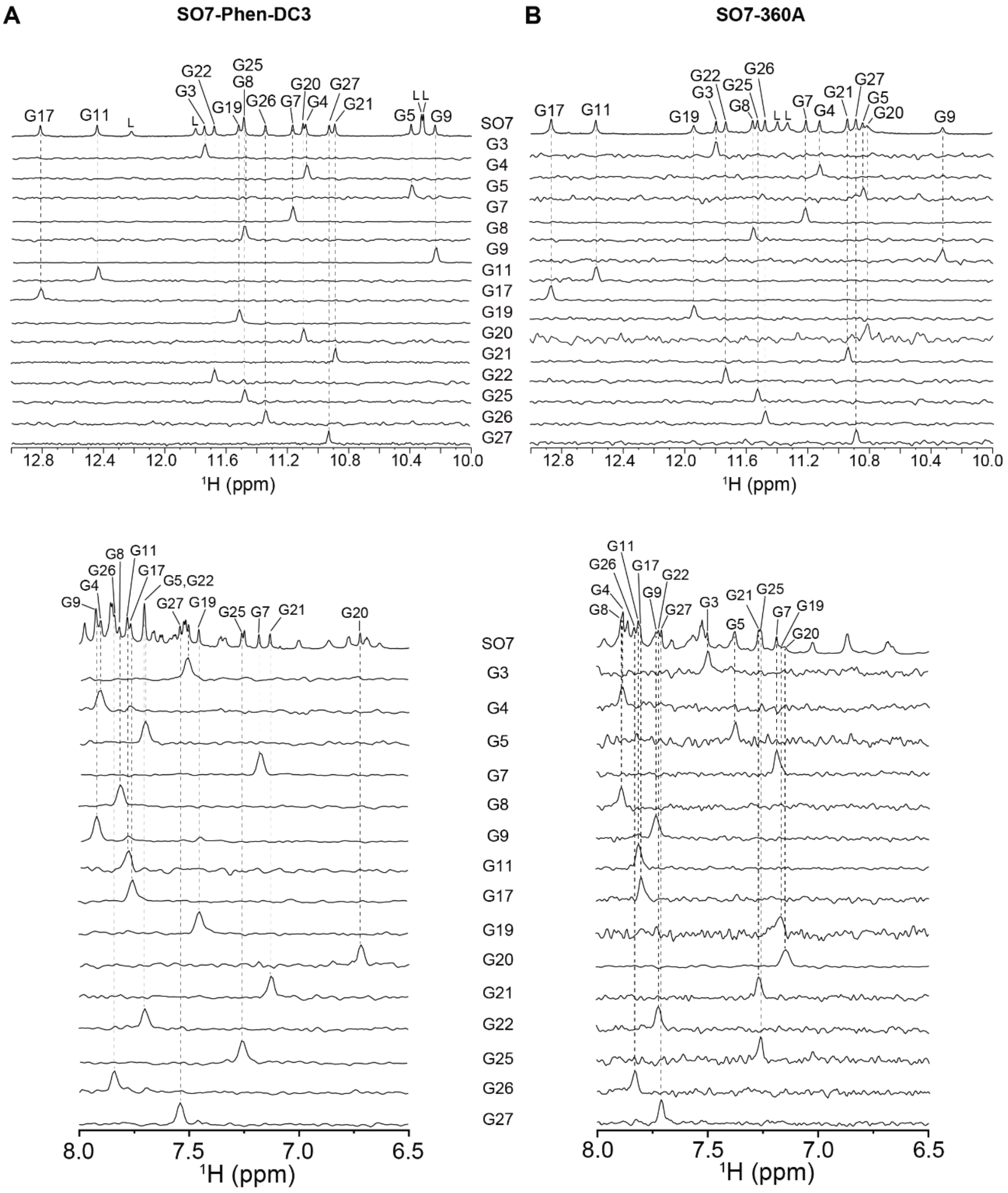

Figure S13. Aromatic-anomeric regions of 2D NOESY spectra (τ_m = 300ms) of the (A) SO7-Phen-DC3 and (B) SO7-360A 1:1 complexes. Guanines in *syn* glycosidic conformations are denoted in bold.

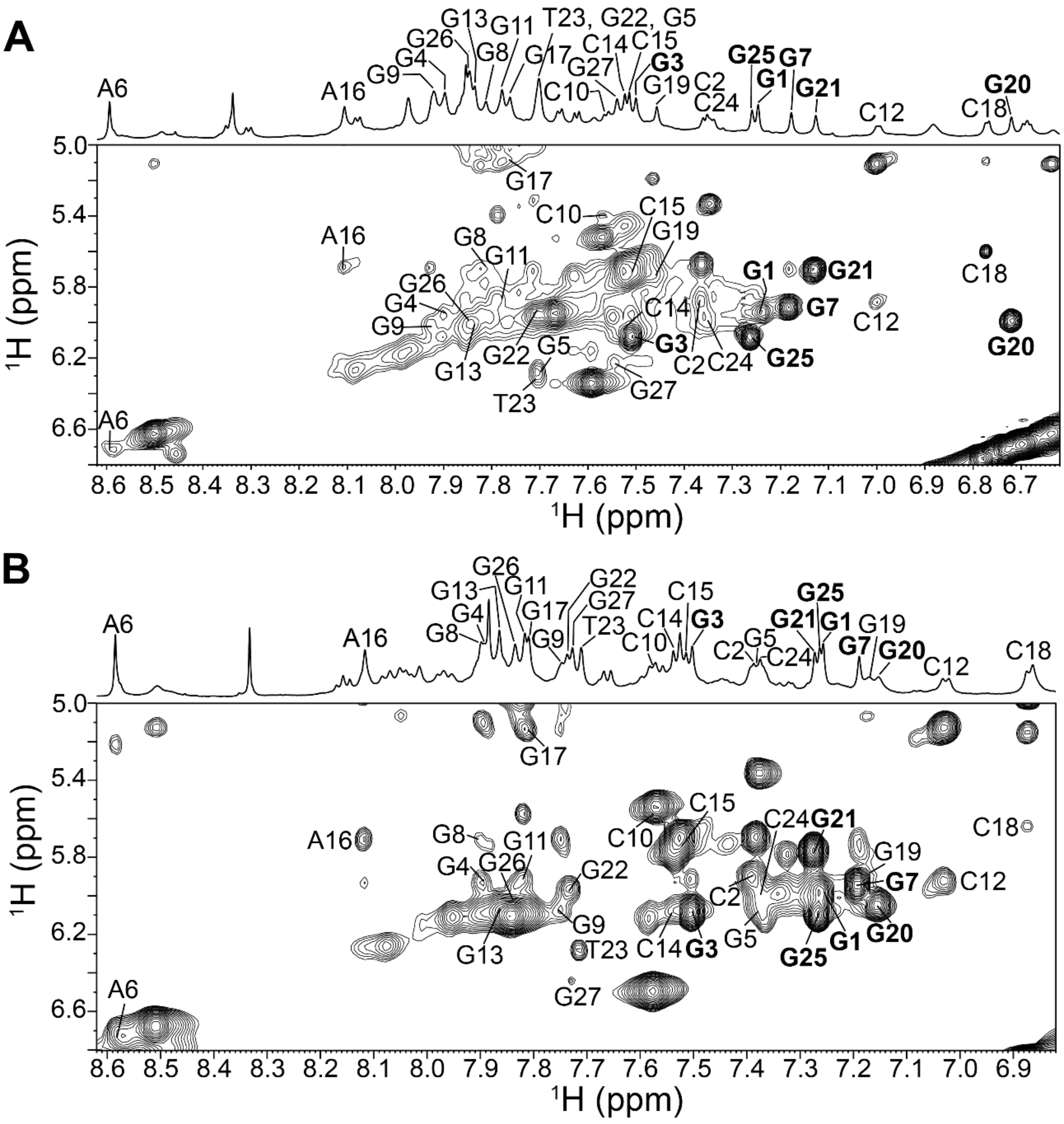

Figure S14. Aromatic regions of 2D ^{1}H-^{13}C HSQC NMR spectra of (A) SO7-Phen-DC3 and (B)SO7-360A 1:1 complexes. *Syn* guanines are denoted in bold. HSQC NMR experiments were recorded in 20 mM potassium phosphate buffer, 20 mM KCl, pH 7.1, at 298.2 K with a 0.5 mM SO7 concentration in 99.94% $^{2}H_2O$ (v/v).

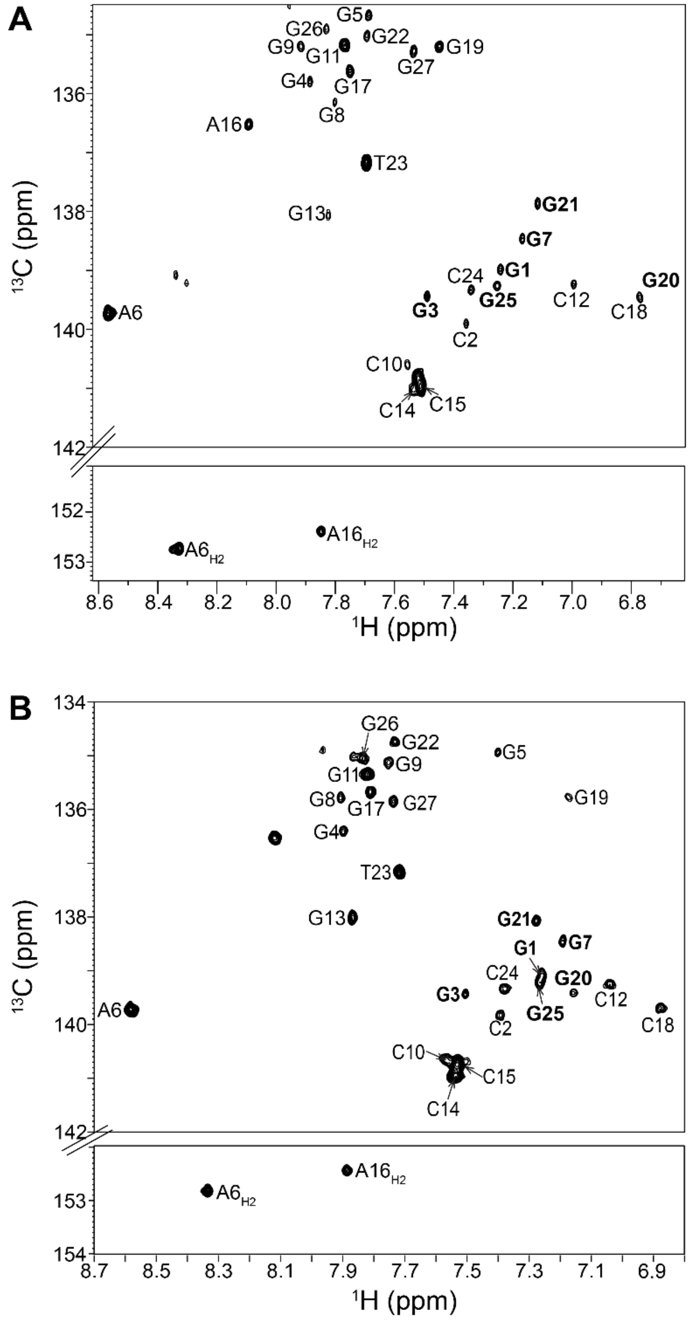

Figure S15. 2D DQF-COSY spectra of (A) SO7-Phen-DC3 and (B) SO7-360A 1:1 complexes showing H1'-H2'/H2'' correlations. Only the H1'/H2' cross peaks are marked for clarity. The * sign denotes residues with isochronous chemical shifts of H2' and H2''. DQF-COSY spectra were performed in 20 mM potassium phosphate buffer, 20 mM KCl, pH 7.1, at 298.2 K with 0.5 mM DNA and ligand concentrations in 99.94% $^{2}H_2O$ (v/v).

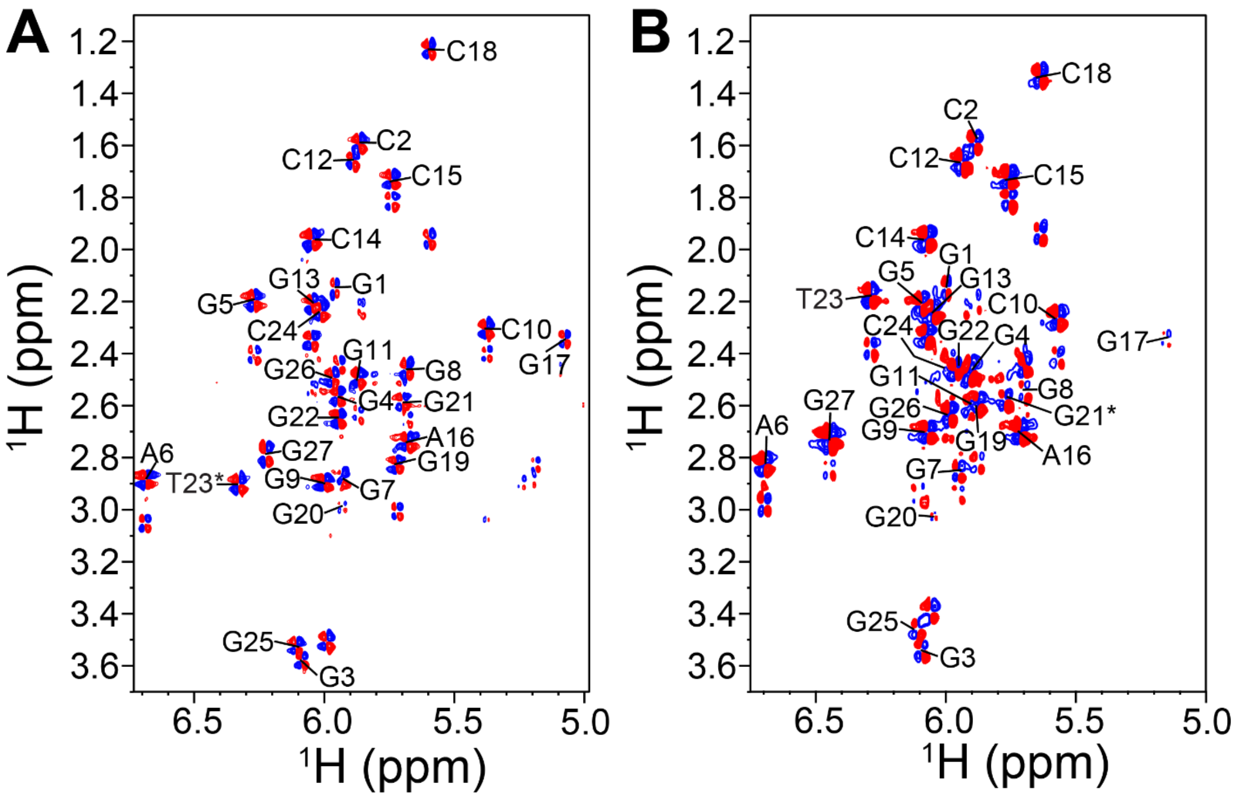

Figure S16. Imino chemical shift plot (CSP) for (A) SO7-Phen-DC3 and (B) SO7-360A 1:1 complexes, respectively. The chemical shift values of free SO7 were derived from the assignment of the free SO7, as reported in (9). The average CSP values are marked with black dotted lines. Guanines engaged in G-quartet and G-C base pair formation are colored grey and magenta, respectively. Guanines in *syn* glycosidic conformation are in bold.

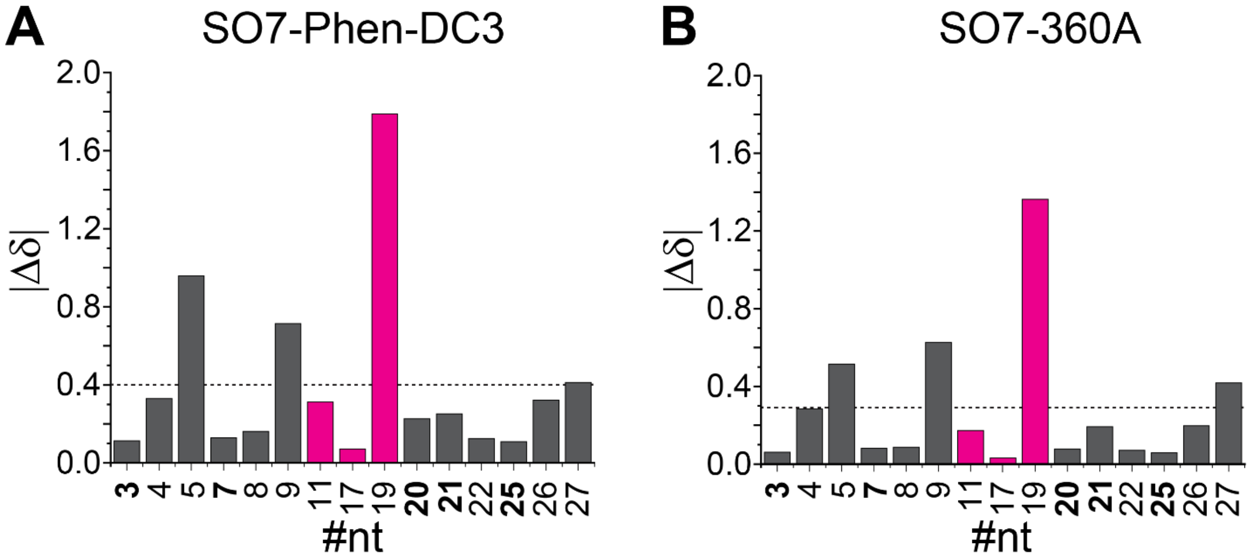

Figure S17. Assignment of Phen-DC3 in the SO7-Phen-DC3 1:1 complex using (A) 2D ^{1}H-^{13}C HSQC and (B) 2D TOCSY (τ_m = 50 ms) NMR spectra. ^{1}H chemical shifts (ppm) of Phen-DC3 are gathered in Table S5. HSQC and TOCSY spectra were performed in 20 mM potassium phosphate buffer, pH 7.1, and 20 mM KCl at 298.2 K with 0.5 mM SO7 and Phen-DC3 concentrations in 99.94% $^{2}H_2O$ (v/v).

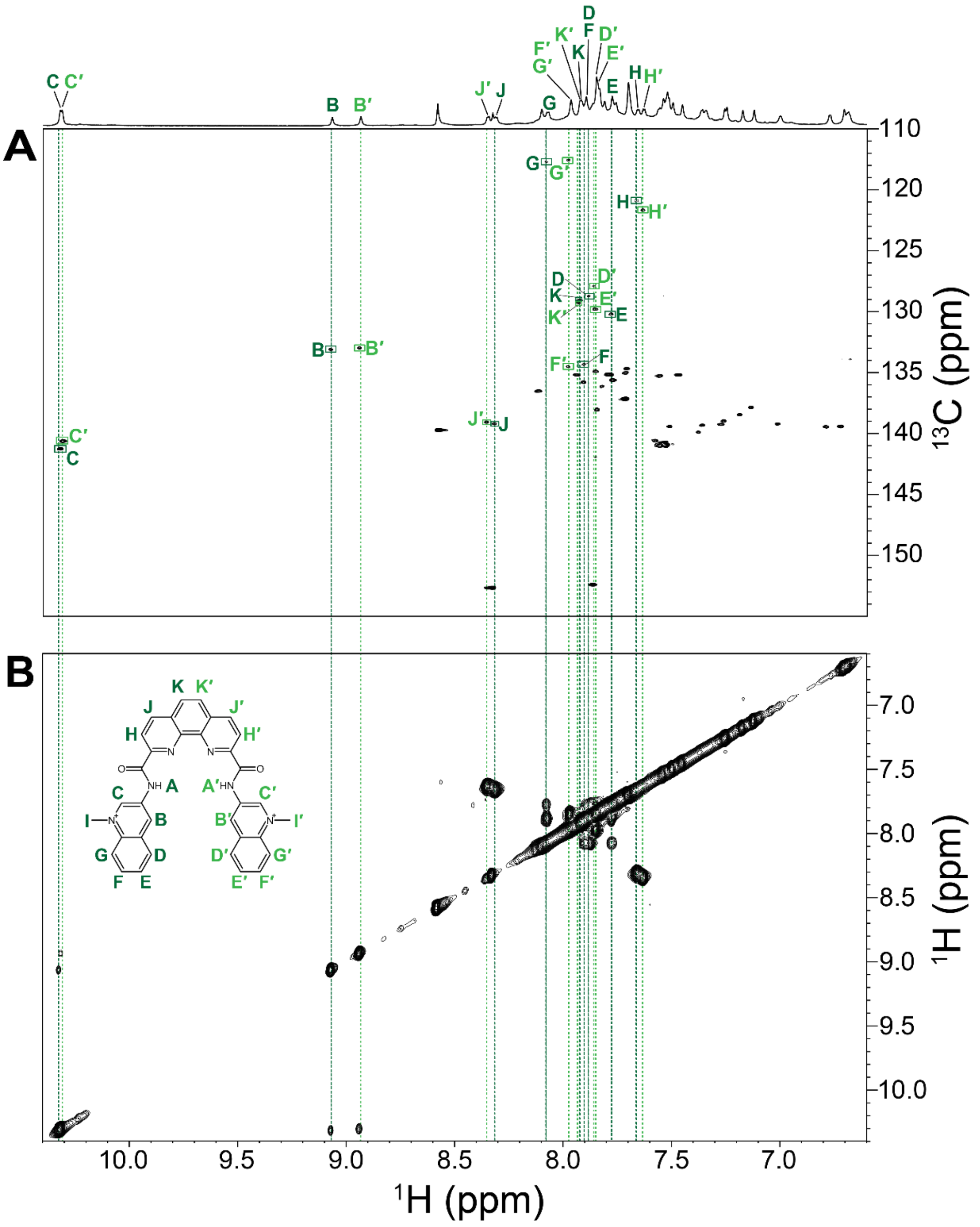

Figure S18. Assignment of 360A in the SO7-360A 1:1 complex using (A) 2D ^{1}H-^{13}C HSQC and (B) 2D TOCSY (τ_m = 50 ms) NMR spectra. ^{1}H chemical shifts (ppm) of 360A are gathered in Table S5. HSQC and TOCSY spectra were performed in 20 mM potassium phosphate buffer, pH 7.1, and 20 mM KCl at 298.2 K with 0.5 mM SO7 and 360A concentrations in 99.94% $^{2}H_2O$ (v/v).

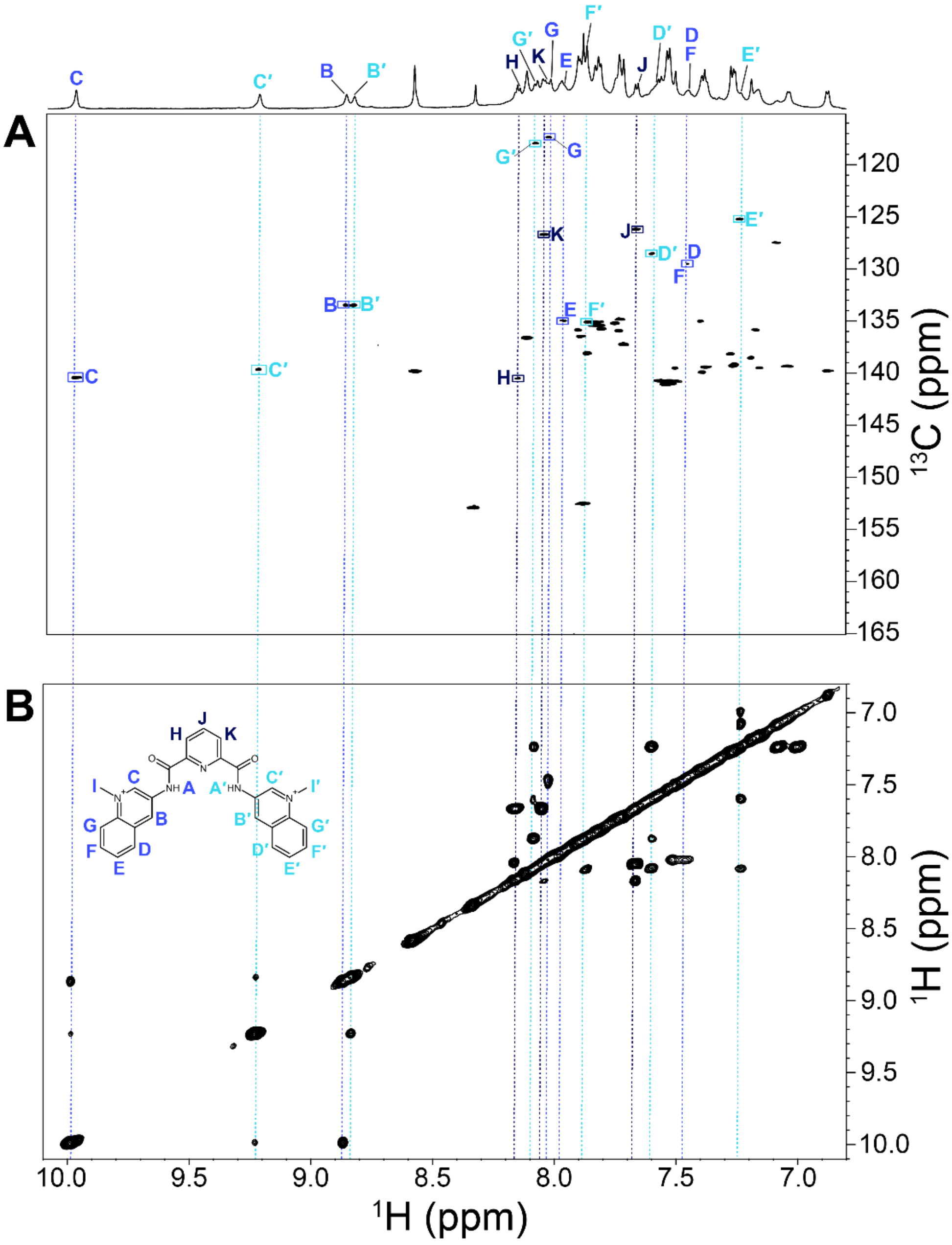

Figure S19. Regions of NOESY spectra (τ_m = 300 ms) showing intermolecular NOE cross-peaks between the SO7 and (A) Phen-DC3 and (B) 360A protons at 1:1 DNA:ligand ratio. NOESY spectra were recorded in 20 mM potassium phosphate buffer, pH 7.1, 20 mM KCl at 298.2 K with 0.5 mM SO7 and ligand concentrations. Phen-DC3 and 360A atoms are marked and colored according to the labels in Figures 3 and 4, respectively.

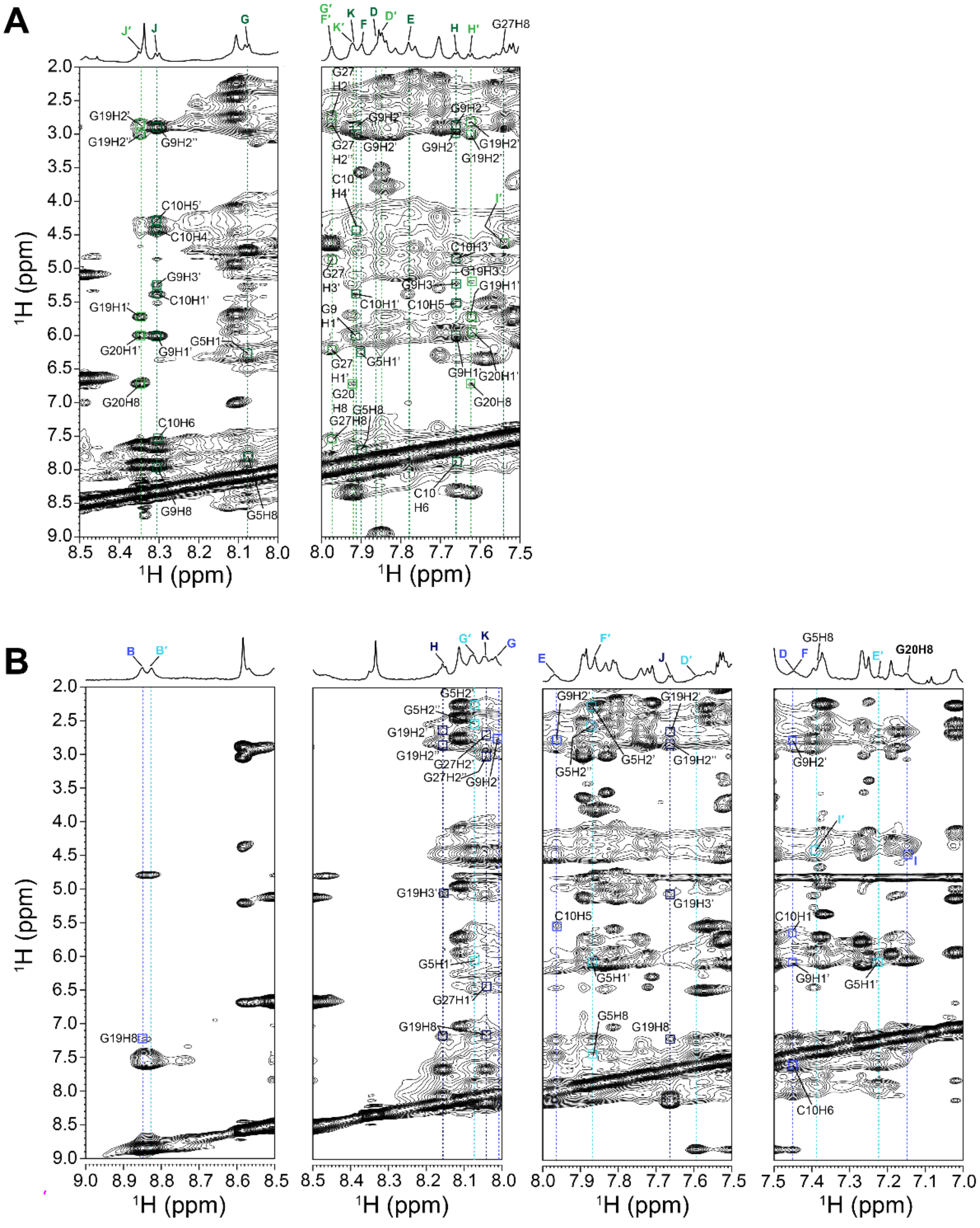

Figure S20. Superposition of the NOESY (τ_m= 300 ms, black color) and EASY-ROESY (τ_m= 200 ms) spectral regions of the (A) SO7-Phen-DC3 and (B) SO7-360A 1:1 complexes, respectively. The ROESY exchange cross-peaks and correlation cross-peaks of the ligand are marked by red and green, respectively. Assignments of ligand protons are shown along the axis. ROESY and NOESY spectra were obtained in 20 mM potassium phosphate buffer, 20 mM KCl, pH 7.1 at 298.2 K with a 0.5 mM DNA and ligand concentrations.

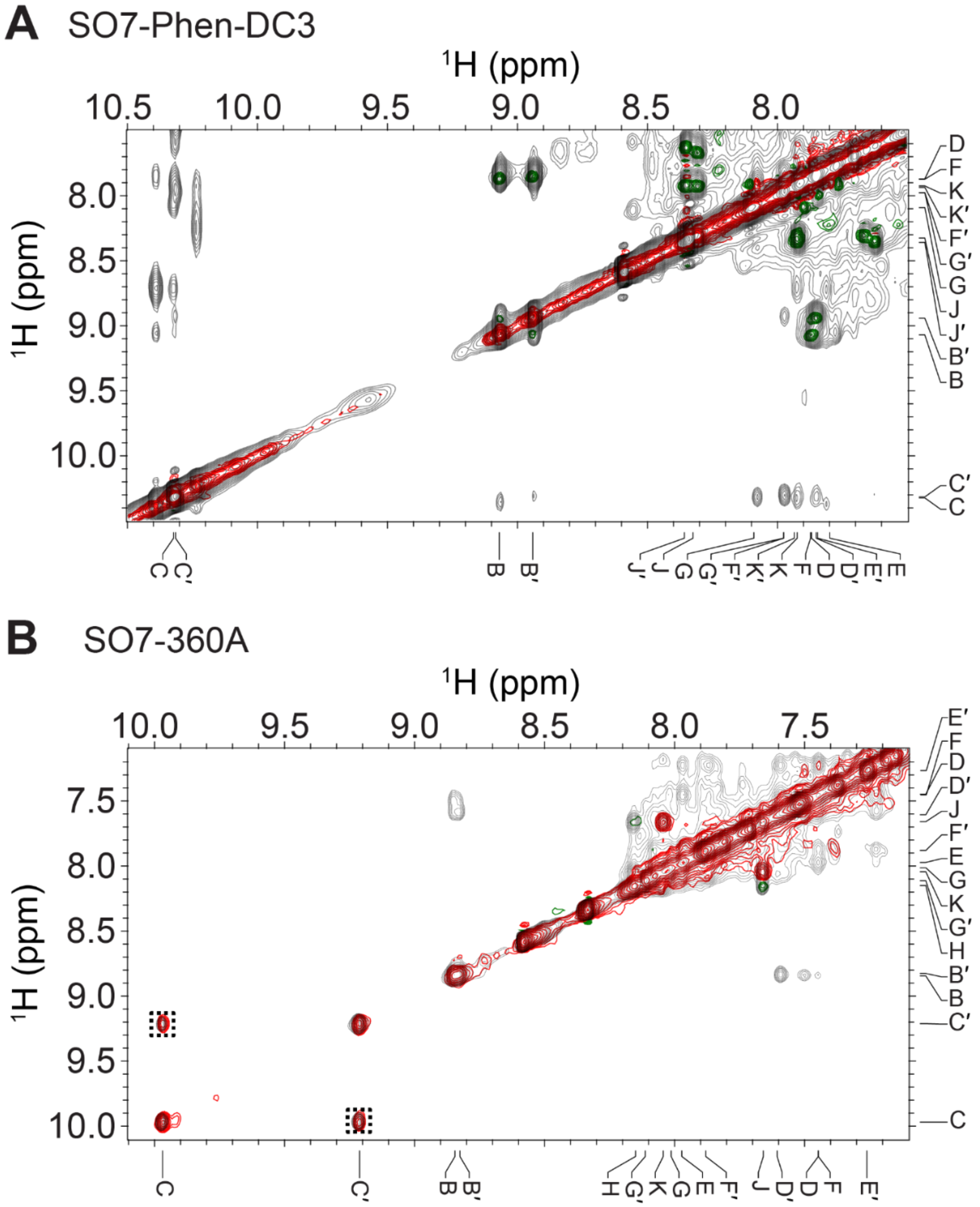

Figure S21. Superposition of the 10 lowest-energy solution structures of (A) SO7-Phen-DC3 and (B) SO7-360A 1:1 complexes, respectively. G-quartet-forming guanines and residues forming G-C base pairs in the stem-loop are marked in grey and magenta, respectively. Phen-DC3 is colored green, while 360A is blue. All the other residues are depicted in light brown.

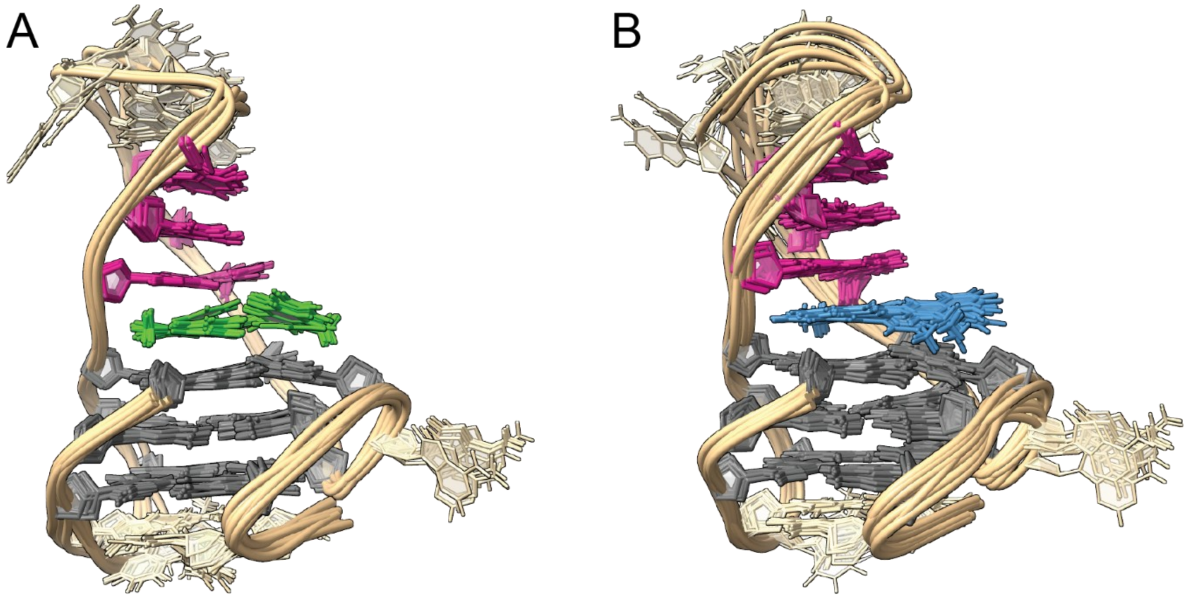

Figure S22. Comparison of stem-loop arrangement, Q-D junction, and stacking interactions at 5′-end of the sequence of (A) SO7 (PDB: 7CV3), (B) SO7-Phen-DC3, and (C) SO7-360A, respectively.

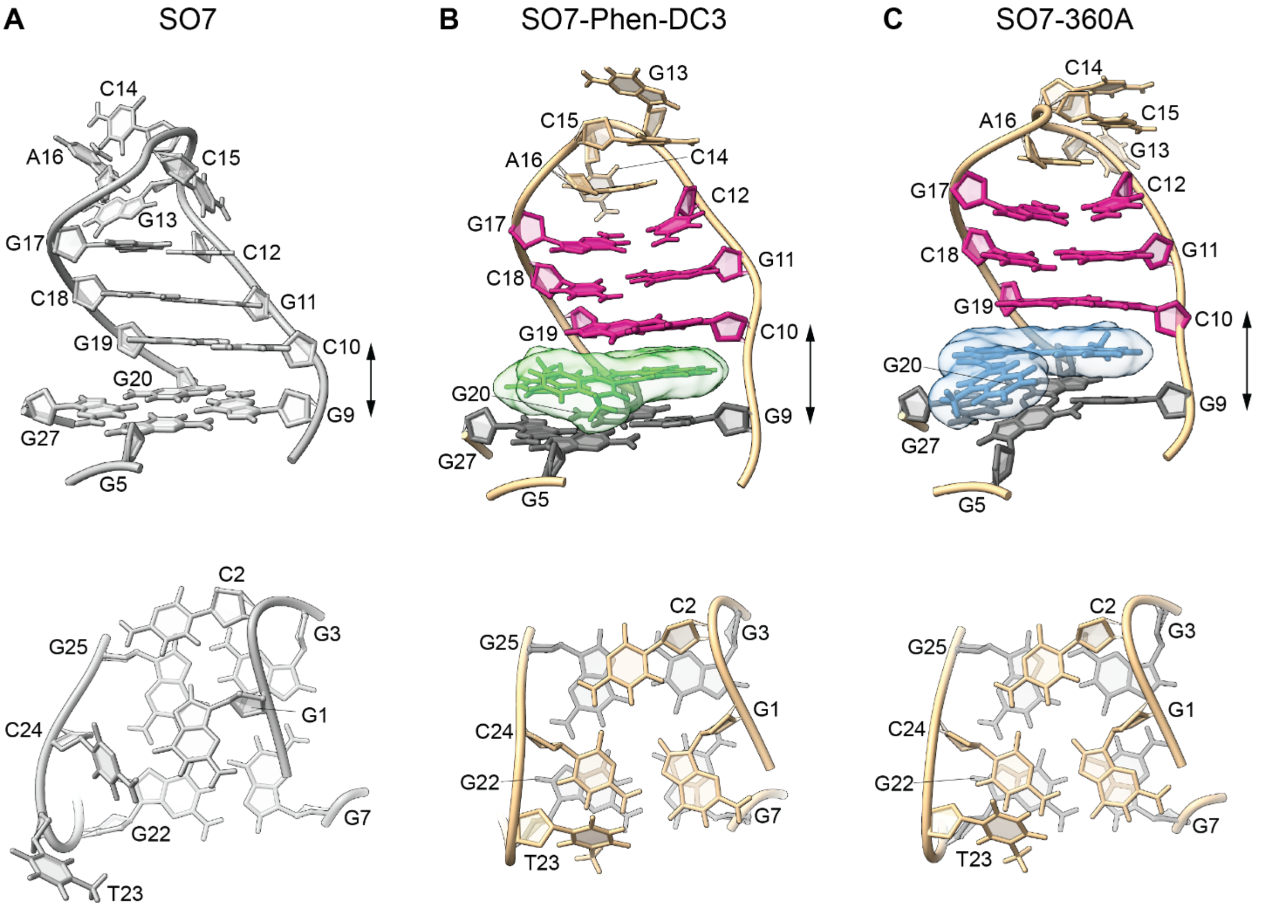

Figure S23. (A-C) The bar plots show groove widths between G-G edges of individual G-quartets of the SO7, SO7-Phen-DC3, and SO7-360A 1:1 complexes. (D) P-P phosphodiester backbone distances between G-C base pairs from the duplex stem-loop. Groove widths and P-P distances were extracted using WEB 3 DNA 2.0 suite and averaged over ten lowest-energy structures. PDB deposition id 7CV3 was used for the analysis of SO7.

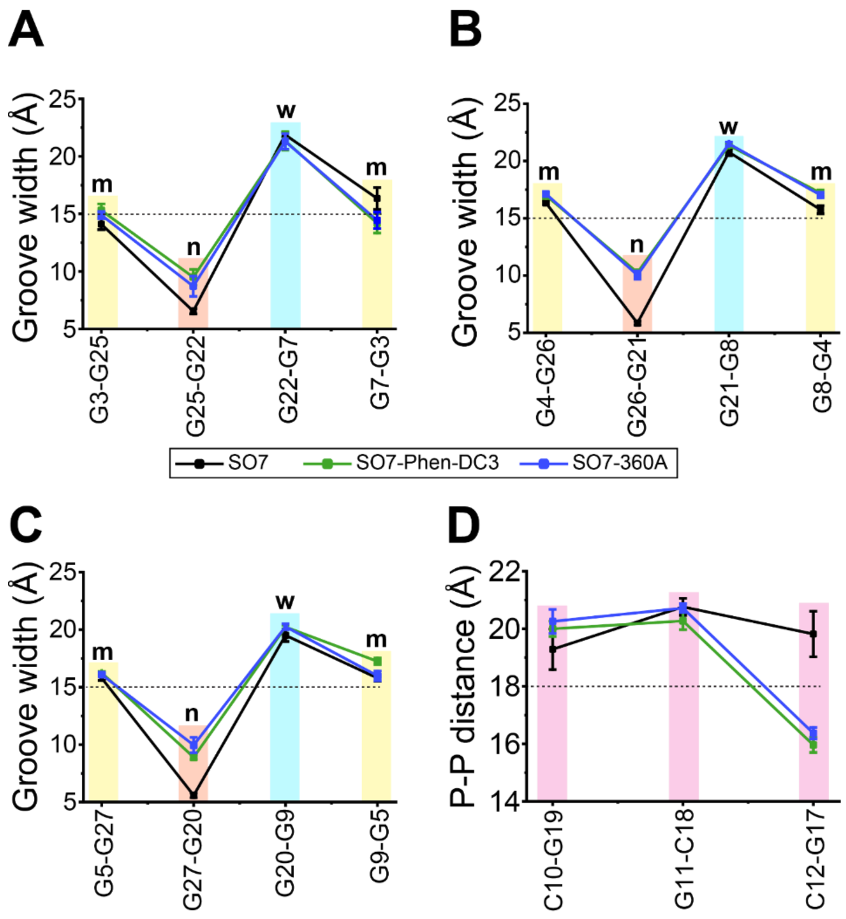

Figure S24. Angular position, used as a collective coordinate in the wt-metaD simulations, describes the twisting motion of the (A) Phen-DC3 and (B) 360A with respect to the DNA. In the close-ups on the left, the pseudotorsion angle is marked by the black dashed line, and its four defining points are shown as big spheres. Each is a geometric center of a group of atoms shown as smaller spheres of the same color. The only exception is the yellow center, the center of the three nearby blue atoms. The ligands are shown in red, the hairpin part of DNA is green, and the G-quartet is ice-blue. The chemical formulas on the right highlight the ligand atoms used to define angular phase, with circle colors corresponding to the sphere colors in the structures on the left.

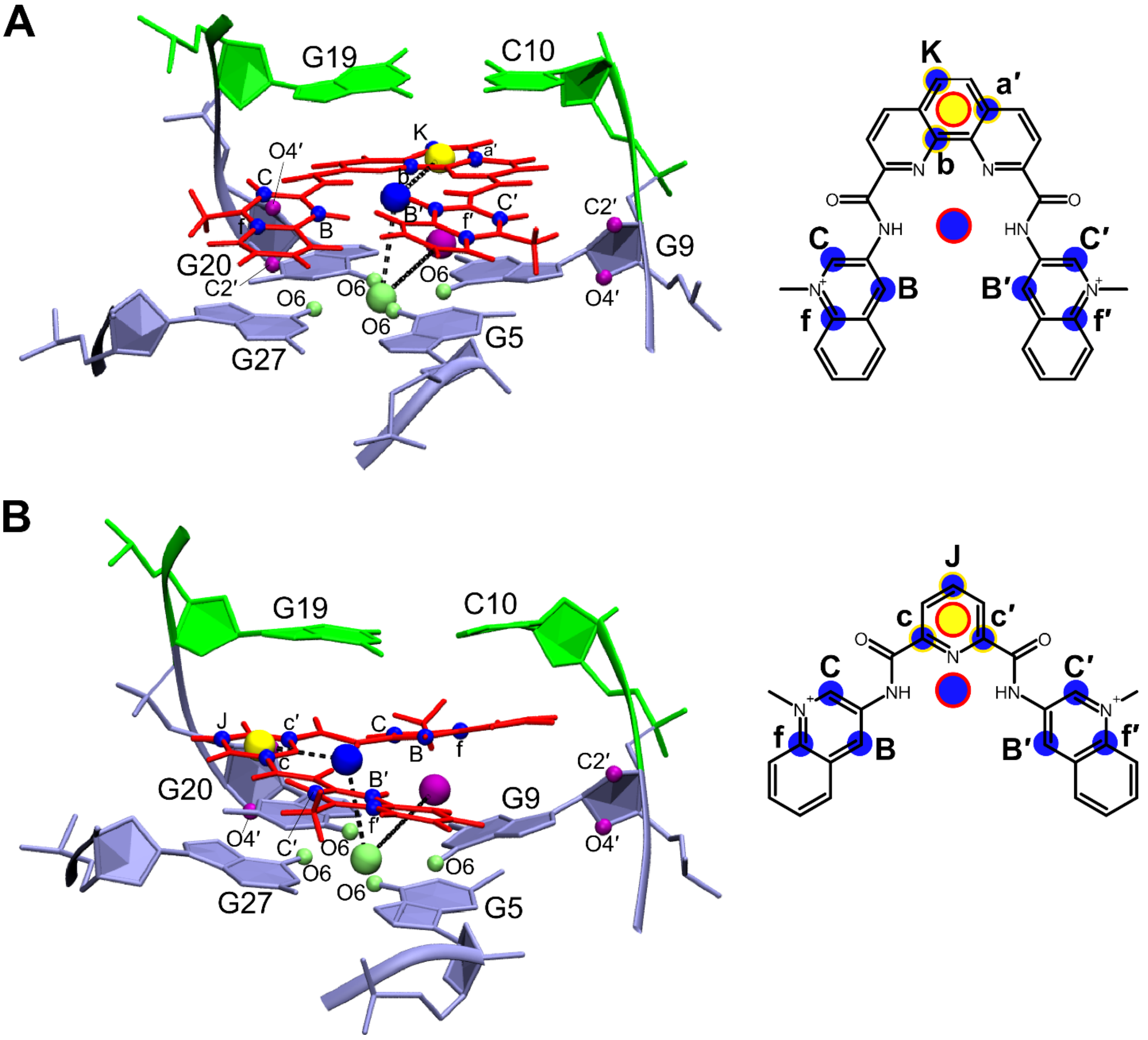

Figure S25. Intercalation of (A) Phen-DC3 and (B) 360A to the Q-D junction of SO7 as observed with MD simulations. While a single binding mode was observed for Phen-DC3 (A and C), 360A occupied two positions with roughly similar populations (B and D).

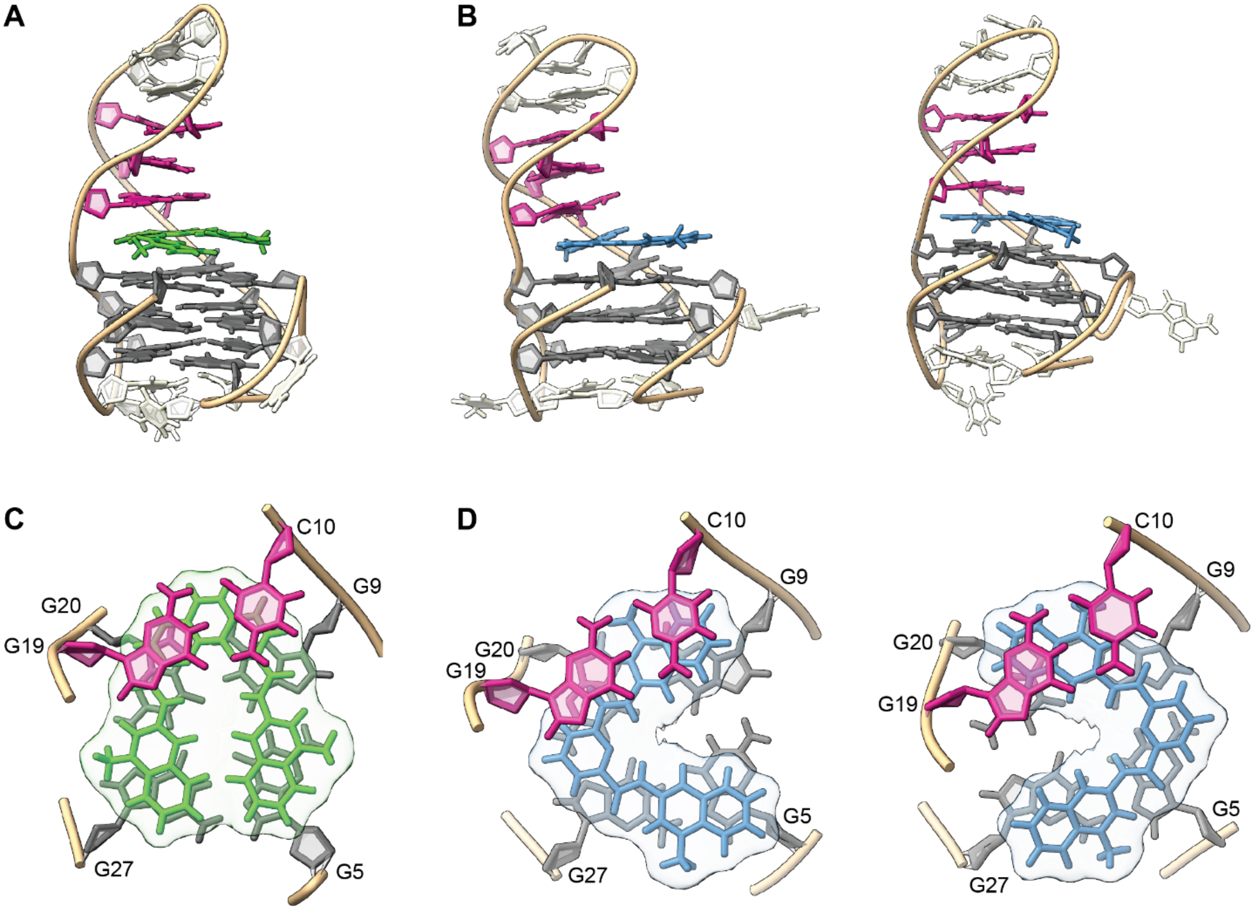

Figure S26. Free-energy plots along the ligand's angular position. (A) Phen-DC3 has a single significant minimum, whereas (B) 360A displays two major minima. The graphs show data from five independent wt-metaD simulations (marked from 1 to 5) and their averaged result (marked as All). Note that the minima in 360A are not as sharply located as in Phen-DC3; this is caused by slightly increased horizontal sliding in 360A, which resulted in a less accurate description of the free-energy profile by the angular position CV. See the Methods or Figure S24 for the definition of the angular position.

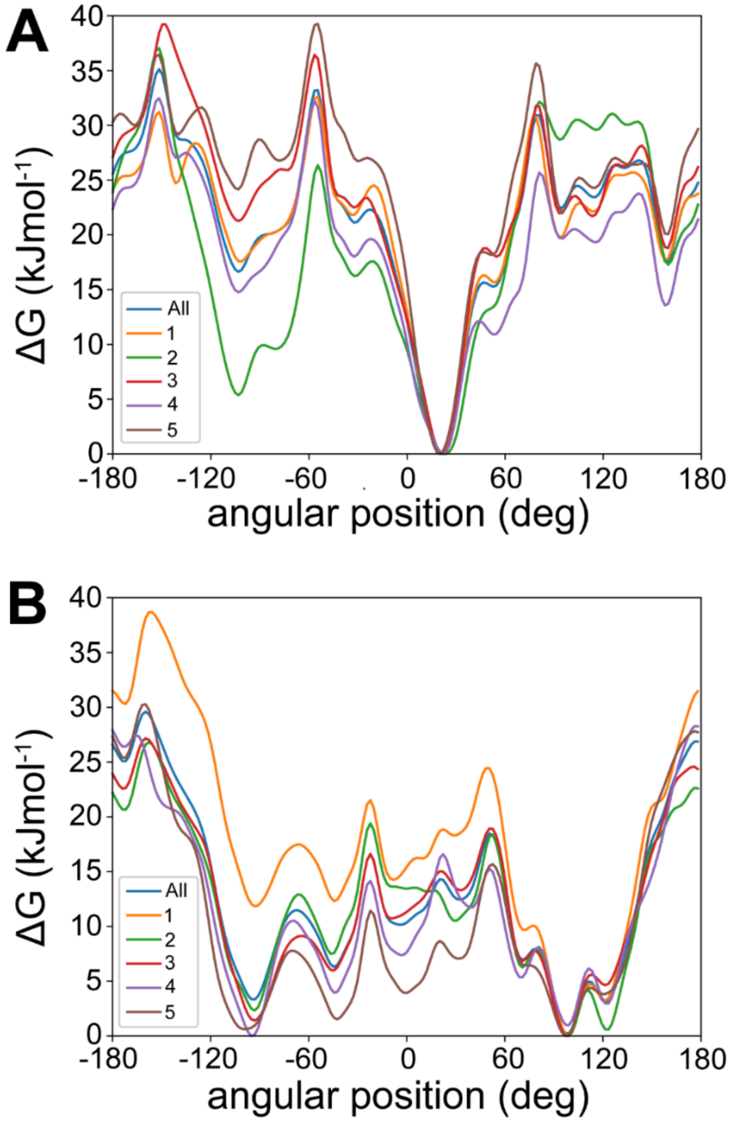

Figure S27. Binding of (A) Phen-DC3 and (B) 360A in the Q-D junction in standard MD simulations, revealing differences in binding modes of the ligands. The position of pivotal point **3** of the ligand (cf. Methods or the blue center in Figure S24) projected into the adjacent G-quartet plane is represented by probability density. The angular position of the ligand is shown by an arrow; the ligand rotation phase of 0 degrees (cf. Figure S26) roughly corresponds to an arrow aiming from the graph center to a virtual point between G9 and G20. The arrow size reflects population density and direction preference at that ligand position – the longer the arrow, the higher the density and the more uniform ligand direction. For clarity, only the positions of the quartet's G(N9) atoms are shown.

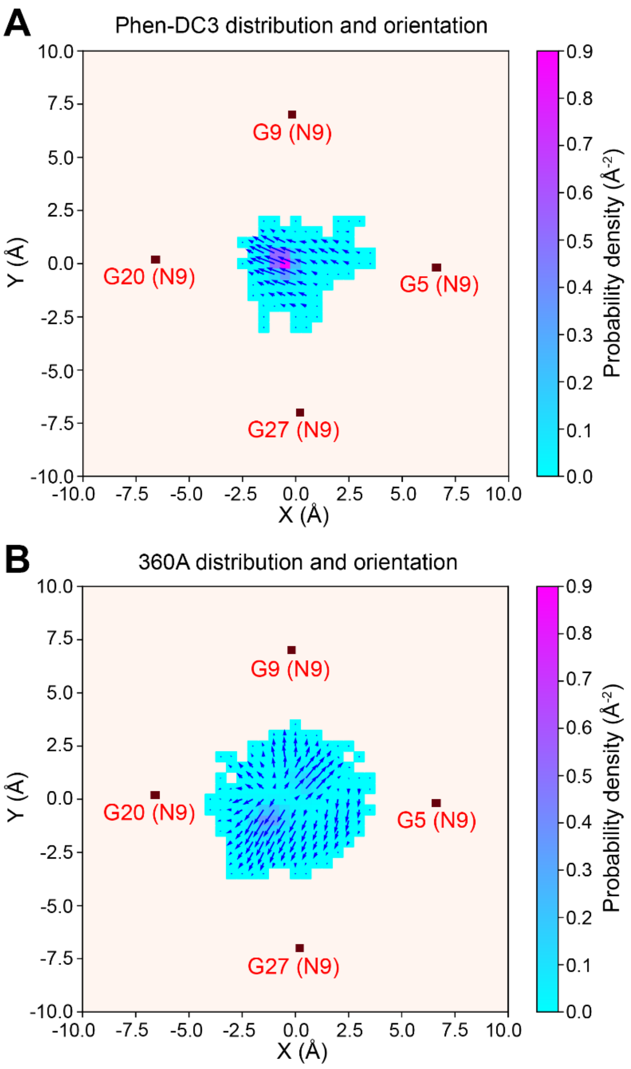

Figure S28. Influence of mutations at the Q-D junction on ligand binding. Constructs with (A) two G-C base pairs ($SO7_{2d}$), (B) one G-C base pair ($SO7_{1d}$), (C) two G-C base pair and a T-T mismatch ($SO7_{TT}$), and (D) no base pair ($SO7_{nd}$) in the stem-loop were tested. Ligands were added to the 1:1 DNA:ligand ratio. NMR spectra were recorded in 20 mM potassium phosphate buffer (20 mM KCl, pH 7.1) at 298.2 K with 0.1 mM DNA and ligand concentrations.

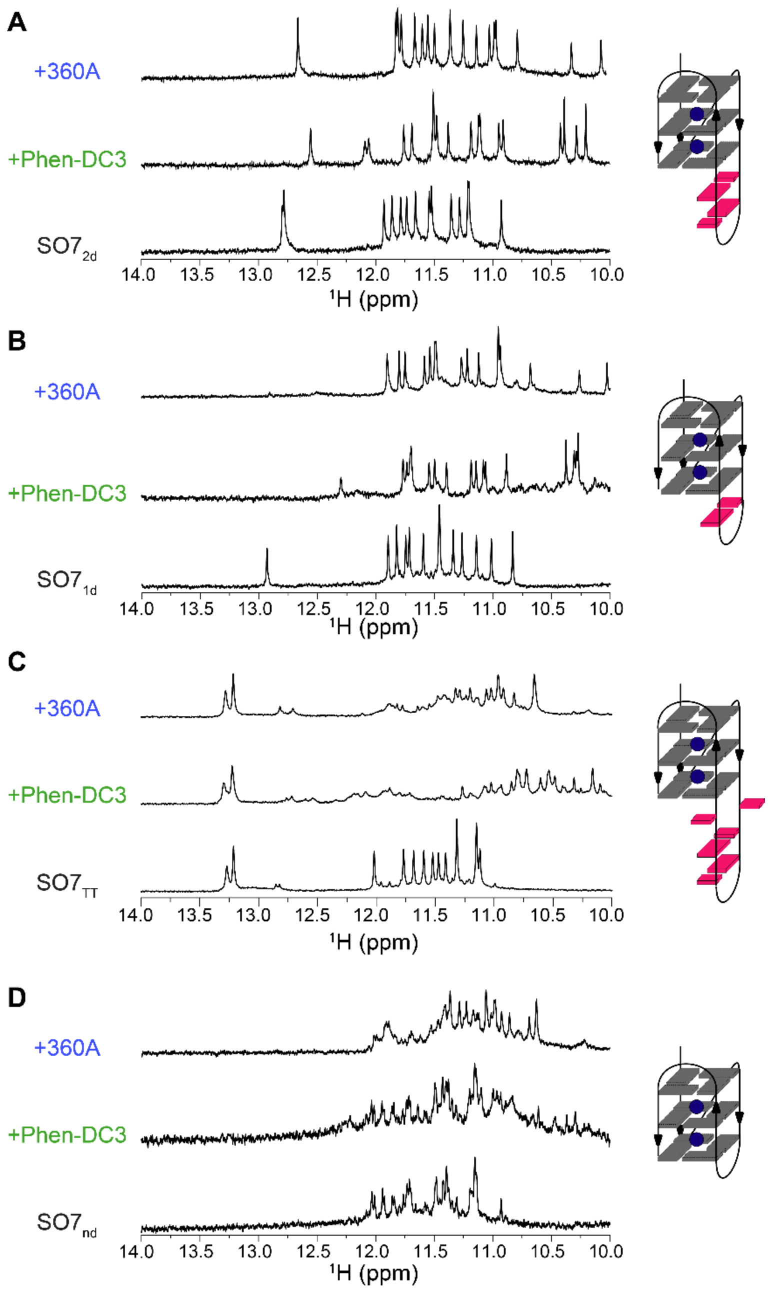

Figure S29. Influence of mutations at the Q-D junction on ligand binding. Constructs with (A) G10-C19 ($SO7_{G10\text{-}C19}$), (B) A10-T19 ($SO7_{A10\text{-}T19}$), and (C) T10-A19 ($SO7_{T10\text{-}A19}$) base pair at the Q-D junction were tested. Ligands were added to the 1:1 DNA:ligand ratio. NMR spectra were recorded in 20 mM potassium phosphate buffer (20 mM KCl, pH 7.1) at 298.2 K with 0.1 mM DNA and ligand concentrations.

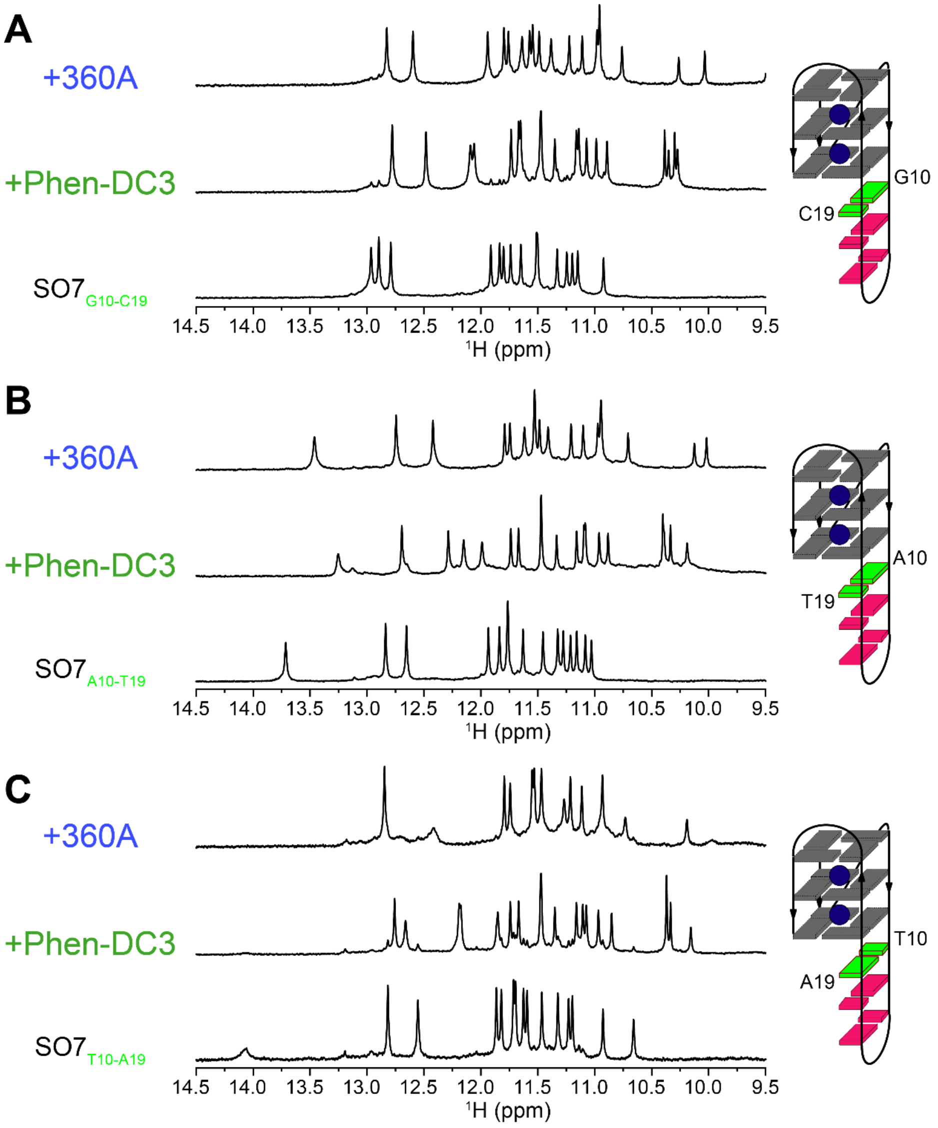

Figure S30. Comparison of *in vitro* ^{1}H and ^{19}F NMR spectra of (A) SO8-F and (B) SO2-F in complex with both ligands at 1:1 ratio, respectively. NMR experiments were performed in 20 mM potassium phosphate buffer, pH 7.1, and 20 mM KCl at 298.2 K with 0.1 mM DNA and ligands concentrations.

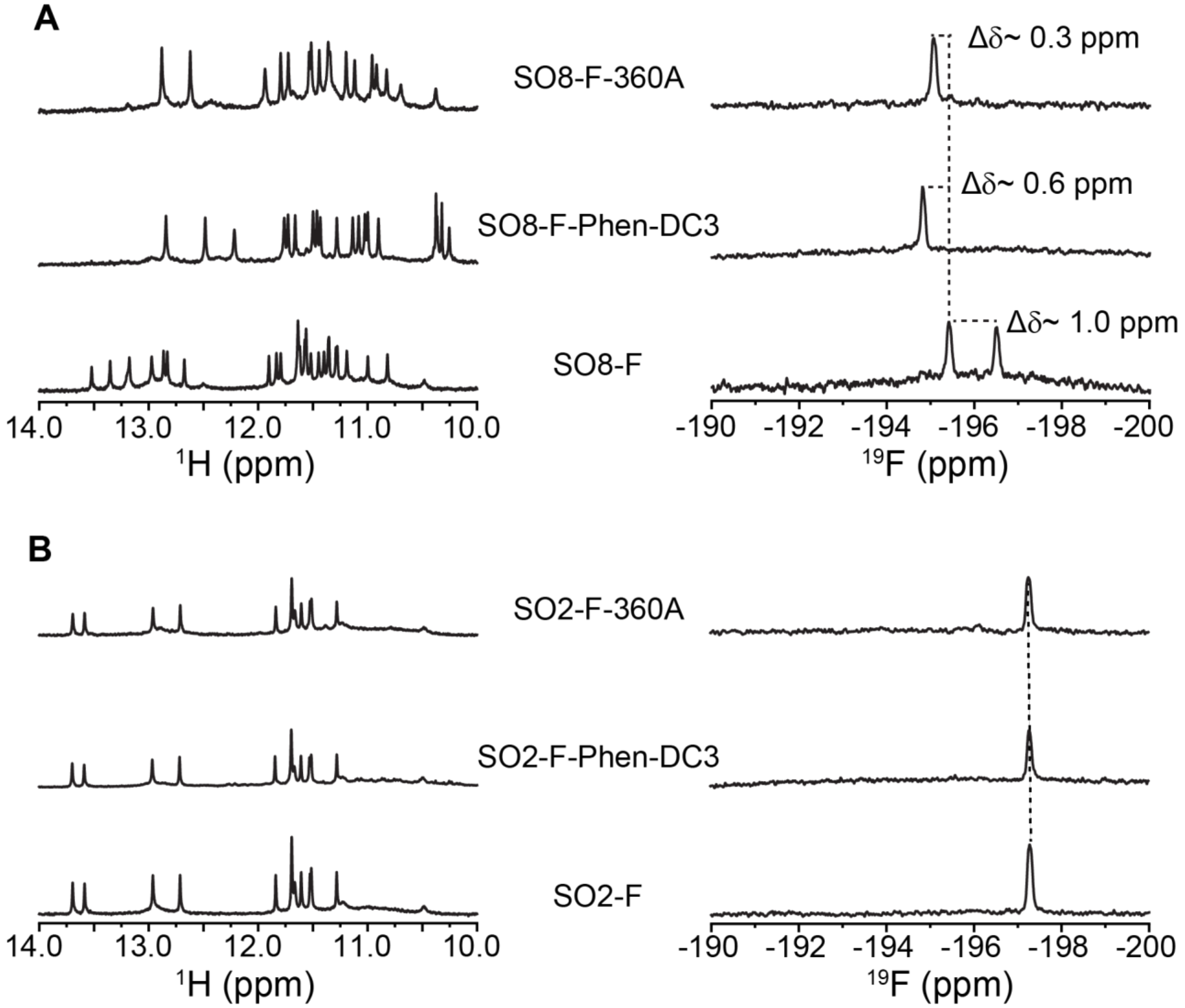

Figure S31: ^{1}H and ^{19}F NMR titration of SO8-F with Phen-DC3 up to 1:3 molar ratio. Red asterisks and grey rectangles indicate imino signals corresponding to hybrid and antiparallel QDH conformations of SO8-F, respectively. Imino signals belonging to the SO8-Phen-DC3 1:1 complex are marked with green dots. Only non-overlapping signals are marked for clarity. Signals in the imino region of ^{1}H NMR spectra belonging to Phen-DC3 are marked with L. The schematics on the right represent the equilibrium of free hybrid (H), free antiparallel (A), and hybrid QDH in complex with one ligand molecule (H+L) at different DNA:ligand ratios. Non-specific ligand binding at 1:2 and 1:3 DNA:ligand ratios is shown as the formation of different QDH complexes to which two or more ligand molecules bind (H+L_n; n≥2). At higher excesses of ligand, the ligand can induce the unfolding of QDH – one of the possibilities is shown.

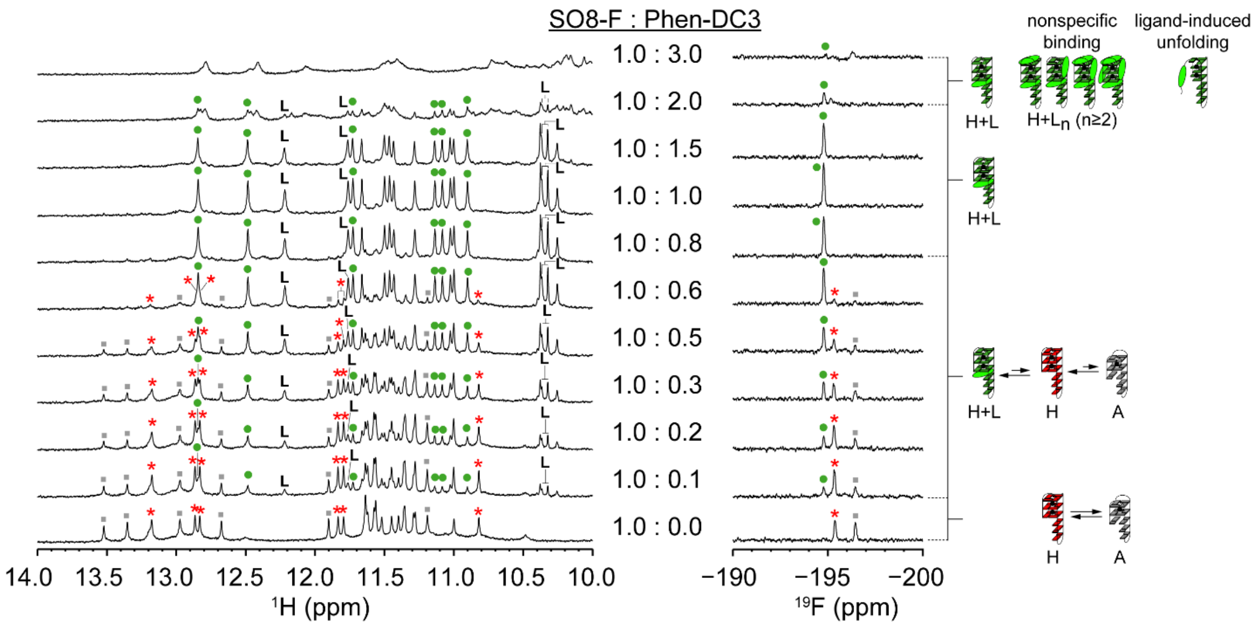

Figure S32: ^{1}H and ^{19}F NMR titration of SO8-F with 360A up to 1:3 molar ratio. Red asterisks and grey rectangles indicate imino signals corresponding to hybrid and antiparallel QDH conformations of SO8-F, respectively. Imino signals belonging to the SO8-360A 1:1 complex are marked with blue dots. Only non-overlapping signals are marked for better clarity. The schematics on the right represent the equilibrium of free hybrid (H), free antiparallel (A), and hybrid QDH in complex with one ligand molecule (H+L) at different DNA:ligand ratios. Non-specific ligand binding at 1:2 and 1:3 DNA:ligand ratios is shown as the formation of different QDH complexes to which two or more ligand molecules bind (H+L_n; n≥2). At higher excesses of ligand, the ligand can induce the unfolding of QDH – one of the possibilities is shown.

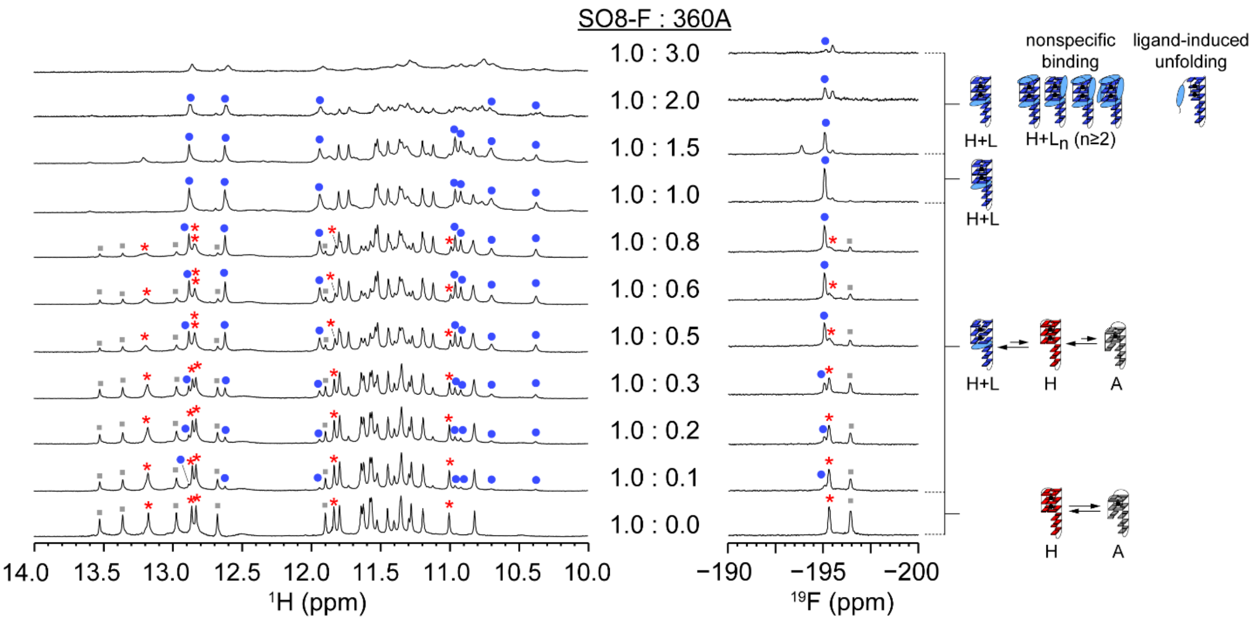

Figure S33. Comparison of *in vitro* and in-cell ^{19}F NMR spectra of SO8-F, SO8-F-Phen-DC3, and SO8-F-360A 1:1 complexes. To evaluate potential degradation due to nuclease activity in cells, we exposed constructs to nuclease benzonase. The effect of benzonase on the ^{19}F spectra is marked by grey rectangle bar and *. To exclude the possibility that the ^{19}F signals result from DNA leaked from cells *via* incisions left after microinjection, we acquired NMR spectra of a supernatant surrounding the cells after the completion of in-cell NMR data acquisition. *In vitro* ^{19}F NMR experiments were performed in 20 mM potassium phosphate buffer, pH 7.1 and 20 mM KCl, at 291.2 K with a 0.1 mM DNA and ligands concentrations.

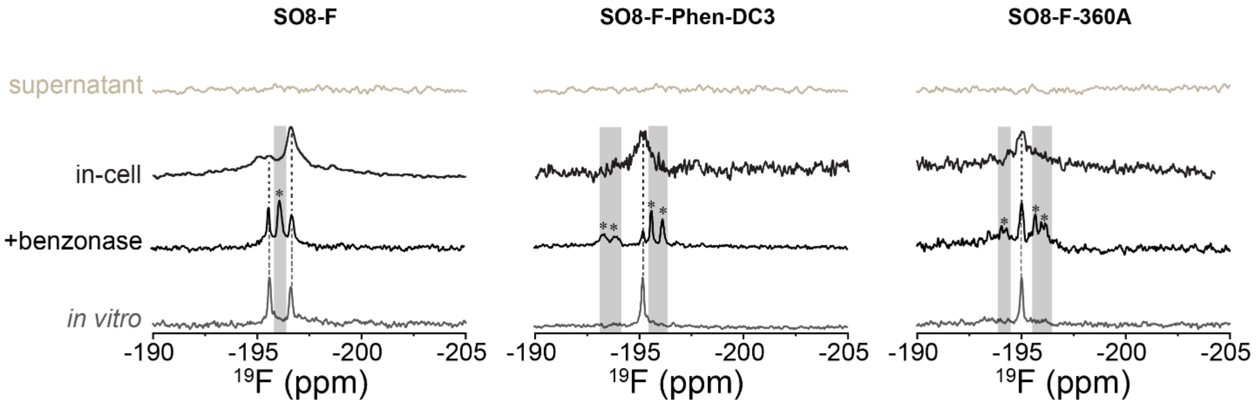

Figure S34. Comparison of (A) ^{1}H and (B) ^{19}F NMR spectra of SO8-F acquired in K^{+}-based (20 mM KPi, 20 mM KCl, pH 7.1; bottom panel) and intraoocyte buffer (top panel). The signals corresponding to the hybrid and antiparallel conformations of the polymorphic QDH construct are indicated with red stars and grey rectangles, respectively. In ^{1}H NMR spectra, only signals between 12.6 and 13.6 ppm were marked for clarity.

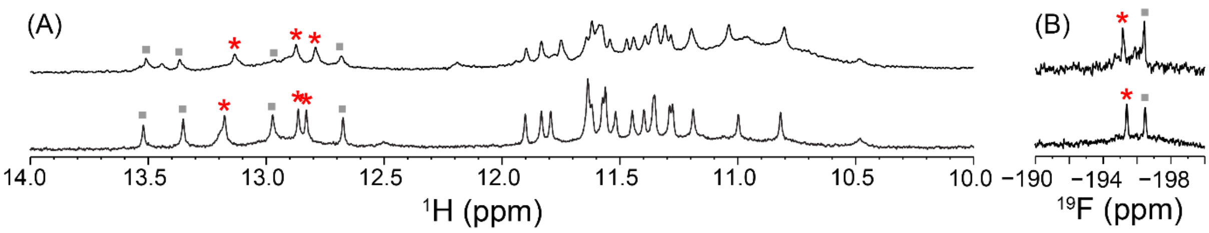

Figure S35. Comparison of ligand (Phen-DC3, 360A, and PDS derivatives) positions in different high-resolution NMR-derived G4 or QDH complex structures: (A) SO7-Phen-DC3 (this work, PDB: 9GVI), (B) Myc24-Phen-DC3 (PDB: 2MGN), (C) QD3-sbl-Phen-DC3 (PDB: 8ABD), (D) 23TAG-Phen-DC3 (PDB: 7Z9L), (E) SO7-360A (this work, PDB: 9GVV), (F) MYT1L-PDS (PDB: 7X3A), (G) MYT1L-PyPDS (PDB: 7X2Z), and (H) VK2-360A (PDB: 6SX3). G-quartet-forming guanines are colored grey, stem-loop-forming base pairs are in magenta, while adenine and cytosines in VK2-360A complex are depicted dark red and yellow, respectively. Phen-DC3 is shown in green, while 360A, PDS and derivates are shown in blue.

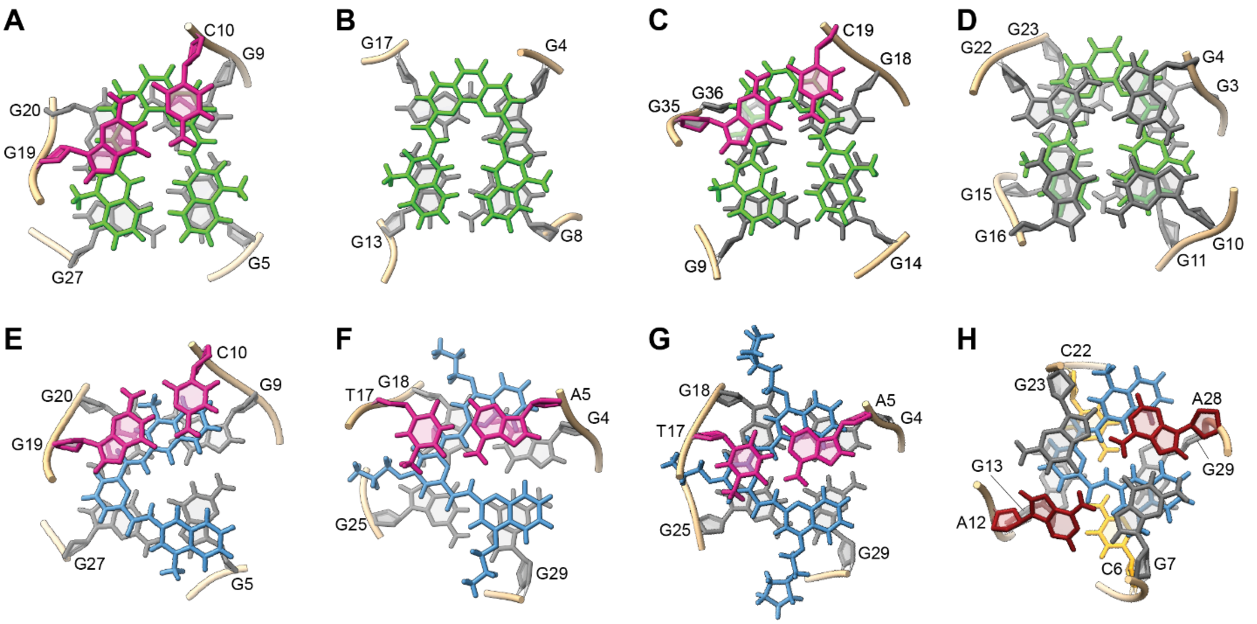

References

1. Dickerhoff,J., Jang,J. and Yang,D. (2024) Best method to determine DNA G-quadruplex folding: The 1H–13C HSQC NMR experiment. *Methods*, **221**, 35–41.

2. Fonville,J.M., Swart,M., Vokáčová,Z., Sychrovský,V., Šponer,J.E., Šponer,J., Hilbers,C.W., Bickelhaupt,F.M. and Wijmenga,S.S. (2012) Chemical Shifts in Nucleic Acids Studied by Density Functional Theory Calculations and Comparison with Experiment. *Chem. Eur. J.*, **18**, 12372–12387.

3. Greene,KarenL., Wang,Y. and Live,D. (1995) Influence of the glycosidic torsion angle on 13C and 15N shifts in guanosine nucleotides: Investigations of G-tetrad models with alternating syn and anti bases. *J. Biomol. NMR*, **5**.

4. Adrian,M., Heddi,B. and Phan,A.T. (2012) NMR spectroscopy of G-quadruplexes. *Methods*, **57**, 11–24.

5. Ghosh,A., Trajkovski,M., Teulade-Fichou,M., Gabelica,V. and Plavec,J. (2022) Phen-DC_3 Induces Refolding of Human Telomeric DNA into a Chair-Type Antiparallel G-Quadruplex through Ligand Intercalation. *Angew. Chem. Int. Ed.*, **61**, e202207384.

6. Chung,W.J., Heddi,B., Hamon,F., Teulade-Fichou,M. and Phan,A.T. (2014) Solution Structure of a G-quadruplex Bound to the Bisquinolinium Compound Phen-DC_3. *Angew. Chem. Int. Ed.*, **53**, 999–1002.

7. Kotar,A., Kocman,V. and Plavec,J. (2020) Intercalation of a Heterocyclic Ligand between Quartets in a G-Rich Tetrahelical Structure. *Chem. Eur. J.*, **26**, 814–817.

8. Vianney,Y.M. and Weisz,K. (2022) High-affinity binding at quadruplex–duplex junctions: rather the rule than the exception. *Nucleic Acids Res.*, **50**, 11948–11964.

9. Tan,D.J.Y., Winnerdy,F.R., Lim,K.W. and Phan,A.T. (2020) Coexistence of two quadruplex–duplex hybrids in the PIM1 gene. *Nucleic Acids Res.*, **48**, 11162–11171.
